# Genetic architecture of quantitative traits in beef cattle revealed by genome wide association studies of imputed whole genome sequence variants: II: carcass merit traits

**DOI:** 10.1186/s12864-019-6273-1

**Published:** 2020-01-13

**Authors:** Yining Wang, Feng Zhang, Robert Mukiibi, Liuhong Chen, Michael Vinsky, Graham Plastow, John Basarab, Paul Stothard, Changxi Li

**Affiliations:** 1 Lacombe Research and Development Centre, Agriculture and Agri-Food Canada, Lacombe, AB Canada; 2 Department of Agricultural, Food and Nutritional Science, University of Alberta, Edmonton, AB Canada; 3 State Key Laboratory for Swine Genetics, Breeding and Production Technology, Jiangxi Agricultural University, Nanchang, Jiangxi China; 4 Present Address: Institute of Translational Medicine, Nanchang University, Nanchang, Jiangxi China; 5 Alberta Agriculture and Forestry, Lacombe Research and Development Centre, 6000 C&E Trail, Lacombe, AB Canada

**Keywords:** Genetic architecture, Imputed whole genome sequence variants, Genome wide association studies, Carcass merit traits, Beef cattle

## Abstract

**Background:**

Genome wide association studies (GWAS) were conducted on 7,853,211 imputed whole genome sequence variants in a population of 3354 to 3984 animals from multiple beef cattle breeds for five carcass merit traits including hot carcass weight (HCW), average backfat thickness (AFAT), rib eye area (REA), lean meat yield (LMY) and carcass marbling score (CMAR). Based on the GWAS results, genetic architectures of the carcass merit traits in beef cattle were elucidated.

**Results:**

The distributions of DNA variant allele substitution effects approximated a bell-shaped distribution for all the traits while the distribution of additive genetic variances explained by single DNA variants conformed to a scaled inverse chi-squared distribution to a greater extent. At a threshold of *P*-value < 10^–5^, 51, 33, 46, 40, and 38 lead DNA variants on multiple chromosomes were significantly associated with HCW, AFAT, REA, LMY, and CMAR, respectively. In addition, lead DNA variants with potentially large pleiotropic effects on HCW, AFAT, REA, and LMY were found on chromosome 6. On average, missense variants, 3’UTR variants, 5’UTR variants, and other regulatory region variants exhibited larger allele substitution effects on the traits in comparison to other functional classes. The amounts of additive genetic variance explained per DNA variant were smaller for intergenic and intron variants on all the traits whereas synonymous variants, missense variants, 3’UTR variants, 5’UTR variants, downstream and upstream gene variants, and other regulatory region variants captured a greater amount of additive genetic variance per sequence variant for one or more carcass merit traits investigated. In total, 26 enriched cellular and molecular functions were identified with lipid metabolisms, small molecular biochemistry, and carbohydrate metabolism being the most significant for the carcass merit traits.

**Conclusions:**

The GWAS results have shown that the carcass merit traits are controlled by a few DNA variants with large effects and many DNA variants with small effects. Nucleotide polymorphisms in regulatory, synonymous, and missense functional classes have relatively larger impacts per sequence variant on the variation of carcass merit traits. The genetic architecture as revealed by the GWAS will improve our understanding on genetic controls of carcass merit traits in beef cattle.

## Background

Carcass merit traits are important to beef production as they directly determine carcass yield, grade, and consumer preferences for meat consumption, and therefore profitability. Genetic improvement of carcass merit traits has been made possible by recording pedigree and/or performance data to predict genetic merit of breeding candidates. However, carcass merit traits are expressed at later stages of animal production and are mostly assessed at slaughter, which sacrifices potential breeding stock although real-time ultrasound imaging technologies can be used to measure some carcass traits such as backfat thickness, longissimus dorsi muscle area, and marbling score on live animals [1]. With the discovery of DNA variants and development of a 50 K SNP panel that covers the whole genome for cattle [2], utilization of DNA markers in predicting genetic merit such as genomic selection holds great promise to accelerate the rate of genetic improvement by shortening the generation interval and/or by increasing the accuracy of genetic evaluation [3, 4]. However, the accuracy of genomic prediction for carcass traits in beef cattle still needs to be improved for wider industry application of genomic selection [5–7]. Although collection of more data on relevant animals to increase the reference population size will improve the genomic prediction accuracy, better understanding on genetic architecture underlying complex traits such as carcass merit traits will help develop a more effective genomic prediction strategy to further enhance feasibility of genomic selection in beef cattle [8, 9].

Early attempts to understanding the genetic control of quantitative traits in beef cattle were made with the detection of chromosomal regions or quantitative trait loci (QTL) [10, 11]. However, these QTLs are usually localized at relatively large chromosomal regions due to relatively low density DNA marker panels used at the time [8, 12, 13]. With the availability of the bovine 50 K SNP chips [2] and high density (HD) SNPs (Axiom™ Genome-Wide BOS 1 Bovine Array from Affymetrix©, USA, termed “HD” or “AffyHD” hereafter), identification of significant SNPs associated with carcass merit traits have led to better fine-mapped QTL regions. All these studies have resulted in multiple QTL candidates for carcass traits in beef cattle, and an extensive QTL database has been created and is available at the Cattle QTL database [14]. In addition, identification of causative mutations underlying the QTL regions has been attempted through association analyses between selected positional and functional candidate gene markers and the traits [10, 15–21]. These identified QTLs and candidate gene markers have improved our understanding on the genetic influence of DNA variants on carcass traits in beef cattle. However, the genetic architecture including causal DNA variants that control the carcass traits still remains largely unknown.

The recent discovery and functional annotation of tens of millions of DNA variants in cattle has offered new opportunities to investigate whole genome wide sequence variants associated with complex traits in beef cattle [22]. The whole genome sequence (WGS) variants represent the ideal DNA marker panel for genetic analyses as they theoretically contain all causative polymorphisms. Although whole genome sequencing on a large number of samples may be impractical and cost prohibitive at present, imputation of SNPs from genotyped lower-density DNA panels such as the 50 K SNP panel up to the WGS level may provide a valuable DNA marker panel for genetic analyses including GWAS due to its high DNA marker density. In a companion study, we imputed the bovine 50 K SNP genotypes to whole genome sequence (WGS) variants for 11,448 beef cattle of multiple Canadian beef cattle populations and retained 7,853,211 DNA variants for genetic/genomic analyses after data quality control of the imputed WGS variants [23]. We also reported the GWAS results for feed efficiency and its component traits based on the 7,853,211 DNA variants in a multibreed population of Canadian beef cattle [23]. The objective of this study was to further investigate the effects of the imputed 7,853,211 WGS DNA variants (or termed as 7.8 M DNA variants or 7.8 M SNPs in the text for simplicity) on carcass merit traits including hot carcass weight (HCW), average backfat thickness (AFAT), rib eye area (REA), lean meat yield (LMY), and carcass marbling score (CMAR).

## Results

### Descriptive statistics and heritability estimates for carcass merit traits

Means and standard deviations of raw phenotypic values for the five carcass merit traits in this study (Table 1) are in line with those previously reported in Canadian beef cattle populations [24, 25]. Heritability estimates of the five carcass merit traits based on the marker-based genomic relationship matrix (GRM) constructed with the 50 K SNP panel ranged from 0.28 ± 0.03 for AFAT to 0.40 ± 0.03 for HCW (Table 1). With the GRMs of the imputed 7.8 M DNA variants, we observed increased heritability estimates for all the five investigated traits, ranging from 0.33 ± 0.03 to 0.35 ± 0.04 (or 6.1% increase) for LMY and from 0.40 ± 0.03 to 0.49 ± 0.03 (or 22.5% increase) for HCW without considering their SE. These corresponded to an increase in additive genetic variances explained by the 7.8 M DNA variants from 5.7% for LMY to 24.0% for HCW, which indicated that the imputed 7.8 M DNA variants were able to capture more genetic variance than the 50 K SNP panel, with different scales of increment depending on the trait. DNA marker-based heritability estimates for all five traits using both 50 K SNPs and imputed 7.8 M DNA variants are slightly smaller than the pedigree based heritability estimates that were obtained from a subset of animals from the population [24], suggesting that neither the 50 K SNP panel nor the 7.8 M DNA variants may capture the full additive genetic variance.

**Table 1:** Descriptive statistics of phenotypic data, additive genetic variances and heritability estimates based on the 50 K SNP and the imputed 7.8 M whole genome sequence (WGS) variants in a beef cattle multibreed population for carcass merit traits

Traits^a^	n	mean (sd)	50 K ${\sigma}_a^2\pm SE$	50 K *h*^2^ ± *SE*	7.8 M ${\sigma}_a^2\pm SE$	7.8 M *h*^2^ ± *SE*
HCW	3984	337.26 (35.42)	335.77 ± 23.39	0.40 ± 0.03	416.26 ± 35.60	0.49 ± 0.03
AFAT	3354	11.11 (4.70)	3.15 ± 0.35	0.28 ± 0.03	3.52 ± 0.50	0.32 ± 0.04
REA	3979	85.46 (11.92)	28.15 ± 2.19	0.36 ± 0.03	32.96 ± 3.34	0.42 ± 0.03
LMY	3367	57.43 (5.02)	3.49 ± 0.34	0.33 ± 0.03	3.69 ± 0.49	0.35 ± 0.04
CMAR	3928	406 (89)	1136.98 ± 104.48	0.29 ± 0.03	1326.30 ± 156.30	0.34 ± 0.03

^a^*HCW* hot carcass weight in kg, *AFAT* average backfat thickness in mm, *REA* rib eye area in cm^2^, *LMY* lean meat yield in %, *CMAR* carcass marbling score from 100 (trace marbling) to 499 (more marbling). mean (SD) = mean of raw phenotypic values and standard deviation (SD), σ_a_^2^ ± SE = additive genetic variance ± standard error (SE), h^2^ ± SE = heritability estimate ± SE

### Comparison of GWAS results between 7.8 M and 50 K SNP panels

At the suggestive threshold of *P*-value < 0.005 as proposed by Benjamin et al. [26], the GWAS of the imputed 7.8 M SNPs detected a large number of SNPs in association with the traits, ranging from 42,446 SNPs for LMY to 45,303 SNPs for AFAT (Table 2). The numbers of additional or novel significant SNPs detected by the 7.8 M DNA panel in comparison to the 50 K SNP GWAS were presented in Table 2, ranging from 31,909 for REA to 34,227 for AFAT. The majority of the suggestive SNPs identified by the 50 K SNP panel GWAS for the five carcass merit traits (ranging from 85% for AFAT to 91% for CMAR) were also detected by the imputed 7.8 M SNP GWAS at the threshold of *P*-value < 0.005. Further investigation showed that all of these suggestive significant SNPs detected by the 50 K SNP panel GWAS were also significant by the 7.8 M SNP GWAS if the significance threshold was relaxed to *P*-value < 0.01, indicating that the imputed 7.8 M SNP panel GWAS was able to detect all the significant SNPs of the 50 K SNP panel. The small discrepancy in *P*-values of each SNP between the two DNA variant panels is likely due to the different genomic relationship matrices used. This result is expected as the 7.8 M DNA variant panel included all SNPs in the 50 K panel and this study used a single marker based model for GWAS. These additional or novel significant SNPs detected by the 7.8 M DNA marker panel corresponded to the increased amount of additive genetic variance captured by the 7.8 M DNA variants in comparison to the 50 K SNP panel, indicating that the imputed 7.8 M DNA variants improved the power of GWAS for the traits. Therefore, we will focus on the GWAS results of the 7.8 M DNA variants in subsequent result sections.

**Table 2:** A summary of number of significant DNA variants detected by the 7.8 M WGS variant GWAS for carcass merit traits in a beef cattle multibreed population

Trait^a^	HCW	AFAT	REA	LMY	CMAR
Suggestive (*p* < 0.005)	42,612 (32,240)	45,303 (34,227)	42,544 (31,909)	42,446 (33,305)	44,654 (33,211)
Lead Suggestive	3927 (3621)	3922 (3598)	3993 (3705)	3906 (3606)	4158 (3827)
Significant (*p* < 10^−5^)	1413 (374)	260 (162)	1171 (254)	312 (198)	256 (145)
Lead Significant	51 (27)	33 (23)	46 (25)	40 (31)	38 (28)
FDR (FDR < 0.10)	1997 (374)	183 (97)	1255 (254)	168 (86)	107 (59)
Lead FDR (FDR < 0.10)	51 (27)	15 (9)	46 (25)	16 (11)	12 (8)

^a^*HCW* hot carcass weight in kg, *AFAT* average backfat thickness in mm, *REA* rib eye area in cm^2^, *LMY* lean meat yield in %, *CMAR* carcass marbling score from 100 (trace marbling) to 499 (more marbling). FDR = genome-wise false discovery rate (FDR) calculated from the Benjamini-Hochberg procedure [27]. The numbers of additional or novel significant SNPs in comparison to the 50 K SNP panel were presented in the parentheses

### DNA marker effects and additive genetic variance related to functional classes

Plots of the allele substitution effects of imputed 7,853,211 WGS variants showed a bell-shaped distribution for all the traits (Additional file 1: Figure S1). Distributions of additive genetic variances explained by single DNA variants followed a scaled inverse chi-squared distribution for all the five traits to a greater extent (Additional file 1: Figure S1). When the DNA marker or SNP effects of the 9 functional classes were examined, differences in their average squared SNP allele substitution effects were observed as shown in Table 3. In general, missense variants, 3’UTR, 5’UTR, and other regulatory region variants exhibited a larger effect on all five carcass merit traits investigated in comparison to DNA variants in other functional classes. Intergenic variants and intron variants captured a greater amount of total additive genetic variance for all five carcass traits. However, the relative proportion of additive genetic variance explained per sequence variant by intergenic and intron variants was smaller than those of other functional classes. Relatively, missense variants captured a greater amount of additive genetic variance per sequence variant for REA, LMY, and CMAR while 3’UTR explained more additive genetic variance per DNA variant for HCW, AFAT, and REA. DNA variants in 5’UTR and other regulatory region variants also showed a greater amount of additive genetic variance explained per sequence variant for CMAR and for CMAR and REA, respectively. Although synonymous variants had relatively smaller averages of squared SNP allele substitution effects, a single DNA variant in the synonymous functional class accounted for more additive genetic variance for AFAT, REA, LMY and CMAR. In addition, both the downstream and upstream gene variants were found to capture more additive genetic variance per sequence variant for HCW (Table 3).

**Table 3:** A summary of SNP allele substitution effects and additive genetic variance for each class based on imputed 7.8 M WGS variant GWAS for carcass merit traits in a beef cattle multibreed population

Trait^a^	Class^b^	no_of_SNP^c^	class_mean^d^	Ratio^e^	Vgf ± SE^f^	Vgo ± SE^g^	Vg_total ± SE^h^	Vgf/SNP^i^	Vgf_Ratio^j^
HCW	Intergenic region variants	5,251,680	7.168859	1.0004	222.95 ± 49.14	188.13 ± 47.74	411.08 ± 48.44	4.24531	0.512854
	Downstream gene variants	253,163	7.520336	1.0495	60.97 ± 39.03	349.56 ± 49.21	410.53 ± 44.47	24.082334	2.909263
	Upstream gene variants	285,798	7.505758	1.0474	39.13 ± 38.31	370.24 ± 49.29	409.37 ± 44.22	13.691187	1.653962
	Synonymous variants	32,019	7.087438	0.9890	2.05 ± 32.98	406.81 ± 46.26	408.86 ± 40.3	6.411609	0.774554
	Intron variants	1,987,366	7.055907	0.9846	143.3 ± 45.55	266.95 ± 49.52	410.25 ± 47.59	7.210423	0.871054
	Missense variants	17,654	7.920537	1.1053	0.000836 ± 26.23	413.7 ± 42.31	413.7 ± 35.35	0.004735	0.000572
	3′ UTR variants	15,851	7.229731	1.0089	2.98 ± 21.86	406 ± 39.82	408.98 ± 32.28	18.816321	2.273103
	5′ UTR variants	3309	7.408689	1.0339	0.000836 ± 16.51	421.81 ± 37.35	421.81 ± 29.07	0.025264	0.003052
	Other regulatory regions	6371	7.792834	1.0875	0.000836 ± 23.25	411.98 ± 40.68	411.98 ± 33.29	0.013122	0.001585
AFAT	Intergenic region variants	5,251,680	0.022854	1.0049	2.75 ± 0.69	0.67 ± 0.64	3.42 ± 0.67	0.052299	0.155457
	Downstream gene variants	253,163	0.023221	1.0210	0.000011 ± 0.55	3.91 ± 0.7	3.91 ± 0.63	0.000004	0.000013
	Upstream gene variants	285,798	0.02344	1.0306	0.000011 ± 0.51	3.6 ± 0.67	3.60 ± 0.60	0.000004	0.000011
	Synonymous variants	32,019	0.022847	1.0046	0.47 ± 0.47	2.98 ± 0.63	3.45 ± 0.56	1.482551	4.406805
	Intron variants	1,987,366	0.022241	0.9779	0.65 ± 0.64	2.78 ± 0.7	3.43 ± 0.67	0.032884	0.097746
	Missense variants	17,654	0.025805	1.1346	0.06 ± 0.37	3.39 ± 0.57	3.44 ± 0.48	0.314206	0.933962
	3′ UTR variants	15,851	0.024082	1.0589	0.18 ± 0.34	3.27 ± 0.54	3.45 ± 0.45	1.145354	3.404504
	5′ UTR variants	3309	0.024767	1.0890	0.000011 ± 0.25	3.45 ± 0.5	3.45 ± 0.40	0.000332	0.000988
	Other regulatory regions	6371	0.024345	1.0704	0.000011 ± 0.36	3.54 ± 0.56	3.55 ± 0.47	0.000173	0.000513
REA	Intergenic region variants	5,251,680	0.138587	0.9972	13.81 ± 4.49	19.18 ± 4.42	32.99 ± 4.46	0.262918	0.032449
	Downstream gene variants	253,163	0.145748	1.0488	6.55 ± 3.73	26.32 ± 4.63	32.87 ± 4.21	2.587783	0.319378
	Upstream gene variants	285,798	0.144594	1.0405	6.7 ± 3.76	26.01 ± 4.66	32.71 ± 4.24	2.343388	0.289216
	Synonymous variants	32,019	0.13927	1.0022	3.89 ± 3.14	28.81 ± 4.34	32.7 ± 3.79	12.144655	1.498865
	Intron variants	1,987,366	0.138017	0.9931	14.76 ± 4.27	18.2 ± 4.57	32.96 ± 4.42	0.742811	0.091676
	Missense variants	17,654	0.160403	1.1542	2.12 ± 2.49	30.79 ± 3.88	32.91 ± 3.27	12.029908	1.484703
	3′ UTR variants	15,851	0.144689	1.0411	1.73 ± 2.1	30.97 ± 3.73	32.71 ± 3.03	10.944533	1.350749
	5′ UTR variants	3309	0.145881	1.0497	0.09 ± 1.48	32.7 ± 3.45	32.79 ± 2.66	2.69595	0.332728
	Other regulatory regions	6371	0.152593	1.0980	1.86 ± 2.28	31.04 ± 3.77	32.9 ± 3.12	29.171151	3.600236
LMY	Intergenic region variants	5,251,680	0.021969	1.0046	2.85 ± 0.68	0.8 ± 0.64	3.66 ± 0.66	0.054312	0.379217
	Downstream gene variants	253,163	0.022022	1.0070	0.00001 ± 0.55	4.09 ± 0.69	4.09 ± 0.62	0.000004	0.000028
	Upstream gene variants	285,798	0.022437	1.0260	0.00001 ± 0.51	3.72 ± 0.66	3.72 ± 0.59	0.000003	0.000024
	Synonymous variants	32,019	0.021771	0.9956	0.31 ± 0.46	3.39 ± 0.62	3.70 ± 0.54	0.95339	6.656776
	Intron variants	1,987,366	0.021469	0.9818	0.73 ± 0.63	2.94 ± 0.69	3.67 ± 0.66	0.036739	0.256519
	Missense variants	17,654	0.024785	1.1334	0.04 ± 0.35	3.65 ± 0.55	3.69 ± 0.46	0.244018	1.703789
	3′ UTR variants	15,851	0.02225	1.0175	0.00001 ± 0.33	3.81 ± 0.54	3.81 ± 0.45	0.000063	0.000440
	5′ UTR variants	3309	0.023045	1.0538	0.00001 ± 0.25	3.72 ± 0.5	3.72 ± 0.4	0.000302	0.002110
	Other regulatory regions	6371	0.022794	1.0423	0.00001 ± 0.36	3.84 ± 0.56	3.84 ± 0.47	0.000157	0.001096
CMAR	Intergenic region variants	5,251,680	6.845916	0.9963	667.29 ± 214.86	600.03 ± 205.38	1267.33 ± 210.16	12.706271	0.015578
	Downstream gene variants	253,163	7.285638	1.0603	192.91 ± 169.25	1081.56 ± 206.95	1274.47 ± 189.17	76.199958	0.093423
	Upstream gene variants	285,798	7.377171	1.0736	330.95 ± 170.69	927.5 ± 208.27	1258.45 ± 190.46	115.797731	0.141971
	Synonymous variants	32,019	7.213334	1.0498	363.28 ± 151.71	897.33 ± 193.21	1260.61 ± 173.75	1134.569787	1.391014
	Intron variants	1,987,366	6.79074	0.9883	385.01 ± 200.95	883.24 ± 220.43	1268.25 ± 210.94	19.372642	0.023751
	Missense variants	17,654	8.065681	1.1738	215.62 ± 124.41	1044.92 ± 177.69	1260.54 ± 153.51	1221.362297	1.497424
	3′ UTR variants	15,851	7.0756	1.0297	0.004 ± 99.99	1284.05 ± 166.53	1284.05 ± 137.72	0.024427	0.000030
	5′ UTR variants	3309	7.507205	1.0925	30.35 ± 72.08	1237.79 ± 152.58	1268.14 ± 119.70	917.210033	1.124525
	Other regulatory regions	6371	8.107115	1.1798	244.87 ± 110.14	1010.4 ± 168.07	1255.27 ± 142.21	3843.536337	4.712283

^a^*HCW* hot carcass weight in kg, *AFAT* average backfat thickness in mm, *REA* rib eye area in cm^2^, *LMY* lean meat yield in %, *CMAR* carcass marbling score from 100 (trace marbling) to 499 (more marbling).^b^Other regulatory regions consisted of splice regions in intron variants, disruptive in-frame deletion, splice region variants, etc. Detail functional class assignments of DNA variants can be found in (Additional file 3: Table S1). ^c^Number of DNA variants (or SNPs in text for simplicity). ^d^class_mean is the average of squared SNP allele substitution effects (class_mean) for the functional class. ^e^Ratio is ratio of the class_mean of the functional class over the weighted average of class_means of all functional classes. ^f^V_gf_ ± SE is additive genetic variance of the functional class ± standard error (SE). ^g^V_go_ ± SE is additive genetic variance of the rest of SNPs in other functional classes ± standard error (SE). ^h^Vg_total ± SE is total additive genetic variance of all 7.8 M WGS variants ± standard error (SE). ^i^V_gf_/SNP is additive genetic variance of the functional class per SNP × 10^5^. ^j^Vgf_Ratio is ratio of additive genetic variance of the functional class per SNP over the average of additive genetic variance per SNP of all functional classes based on the imputed 7.8 M WGS variant GWAS

### Top significant SNPs associated with carcass merit traits

**Fig. 1:**
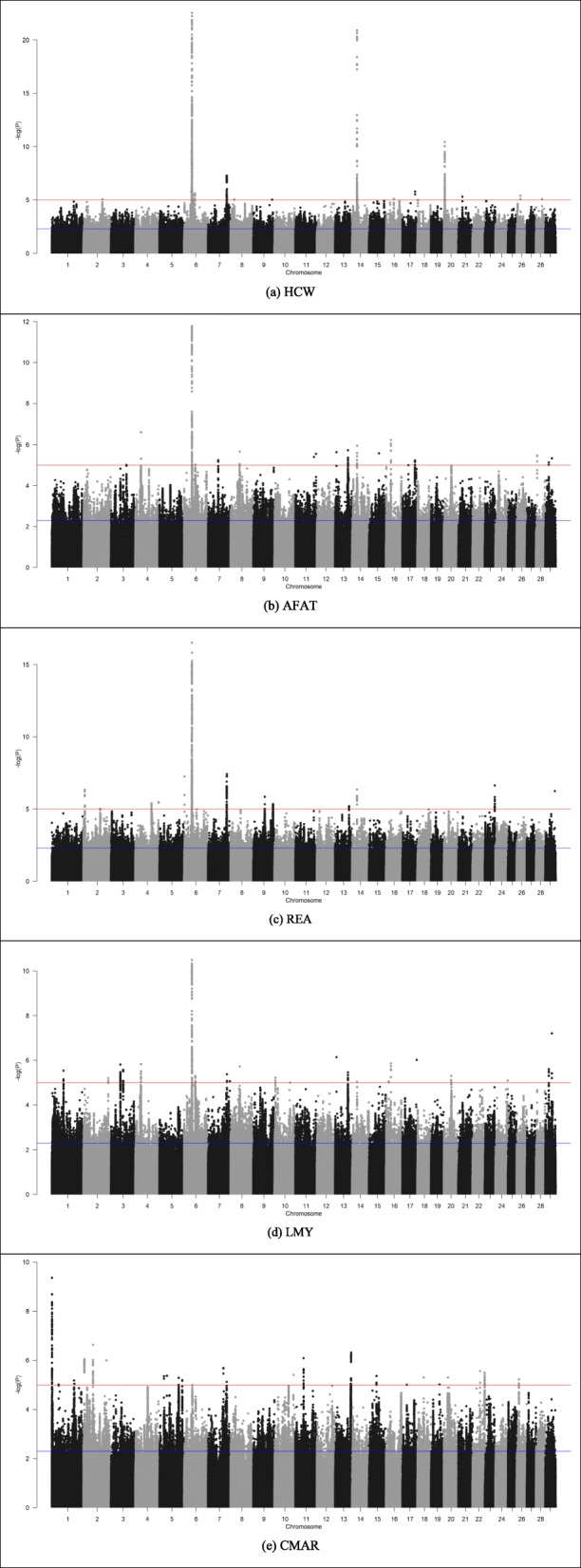
Manhattan plots of GWAS results based on the imputed 7.8 M DNA variant panel for (**a**) hot carcass weight (HCW), (**b**) average backfat thickness (AFAT), (**c**) rib eye area (REA), (**d**) lean meat yield (LMY), and (**e**) carcass marbling score (CMAR). The vertical axis reflects the –log_10_ (*P*) values and the horizontal axis depicts the chromosomal positions. The blue line indicates a threshold of *P*-value < 0.005 while the red line shows the threshold of *P*-value < 10^− 5^

The suggestive lead SNPs associated with HCW, AFAT, REA, LMY, and CMAR in Table 2 were distributed across all the autosomes as shown in the Manhattan plots of 7.8 M DNA variant GWAS (Fig. 1). The numbers of lead SNPs were dropped to 51, 33, 46, 40, and 38 for HCW, AFAT, REA, LMY, and CMAR, respectively, at a more stringent threshold of *P*-value < 10^− 5^, of which 51, 15, 46, 16, and 12 lead significant SNPs reached a FDR < 0.10 for HCW, AFAT, REA, LMY, and CMAR, respectively (Table 2).

**Fig. 2:**
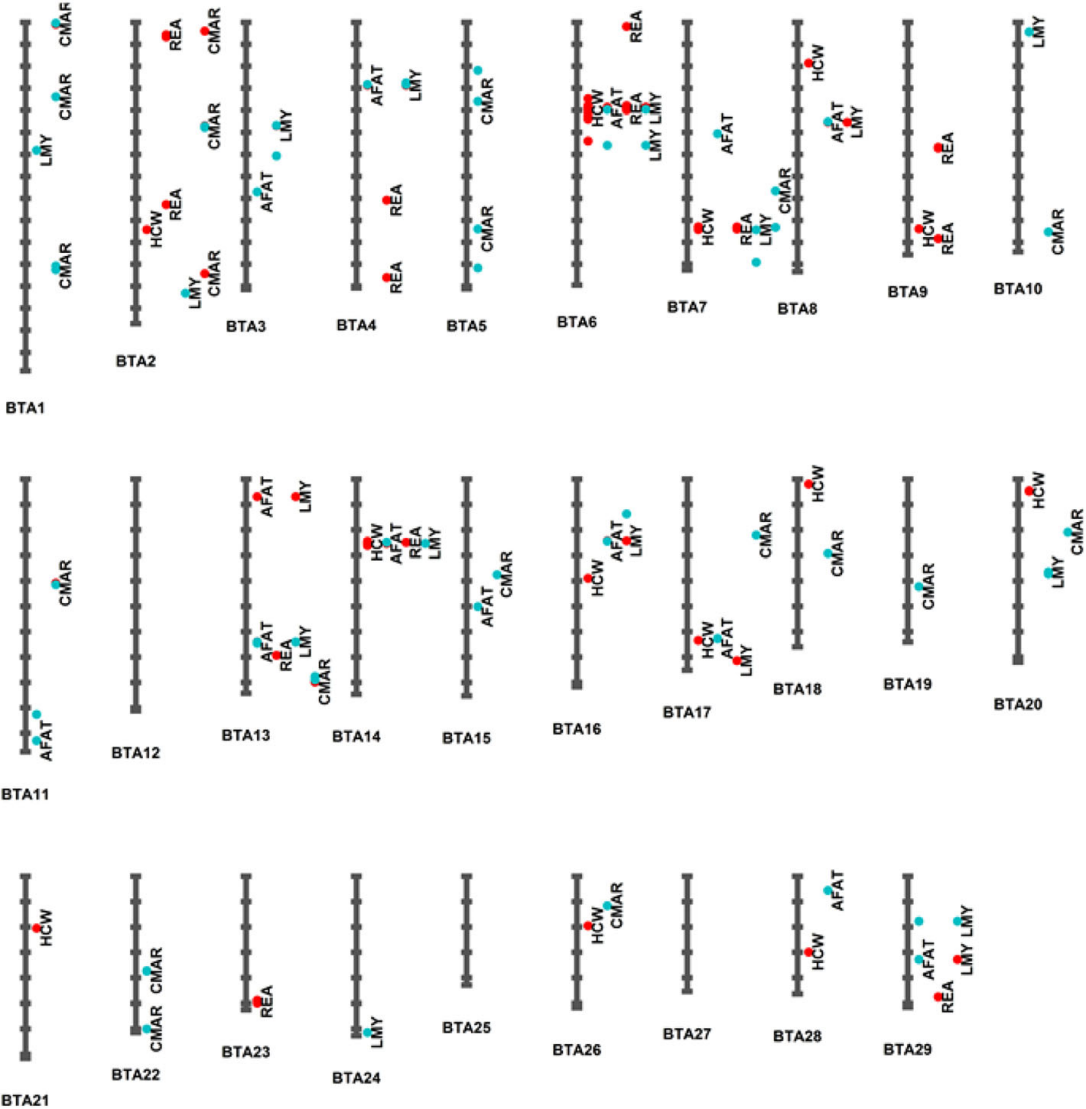
Distribution of lead SNPs at *P*-value < 10^− 5^ on *Bos taurus* autosomes (BTA) for hot carcass weight (HCW), average backfat thickness (AFAT), rib eye area (REA), lean meat yield (LMY), and carcass marbling score (CMAR). The blue dots indicate a threshold of *P-*value < 10^− 5^ while the red dots show the threshold of both *P-*value < 10^− 5^ and genome-wise false discovery rate (FDR) < 0.10

The lead significant SNPs at the nominal *P*-value < 10^− 5^ for the five carcass merit traits were distributed on multiple autosomes (Fig. 2). In general, SNP with larger effects were observed on BTA6 for HCW, AFAT, LMY, and REA. For CMAR, SNPs with relatively larger effects were located on BTA1 and BTA2 (Additional file 2). To show lead SNPs on each chromosome, Table 4 lists top significant lead SNPs with larger phenotypic variance explained on each chromosome. The top lead variant Chr6:39111019 for HCW on BTA6 was an INDEL located 118,907 bp from gene *LCORL* and explained 4.79% of the phenotypic variance. SNP *rs109658371* was another lead SNP on BTA6 and it explained 4.65% of phenotypic variance for HCW. Additionally, SNP *rs109658371* was located 102,547 bp upstream of the top SNP Chr6:39111019 and it is 221,454 bp away from the nearest gene *LCORL*. Outside BTA6, two other SNPs *rs109815800* and *rs41934045* also had relatively large effects on HCW, explaining 3.41 and 1.47% of phenotypic variance and are located on BTA14 and BTA20, respectively. SNPs *rs109815800* is 6344 bp away from gene *PLAG1* whereas SNP *rs41934045* is located in the intronic region of gene *ERGIC1*. For AFAT, two lead SNPs explaining more than 1% of phenotypic variance included SNP *rs110995268* and SNP *rs41594006*. SNP *rs110995268* is located in the intronic region of gene *LCORL* on BTA6, explaining 2.87% of phenotypic variance. SNP *rs41594006*, which explained 1.07% of phenotypic variance, is 133,040 bp away from gene *MACC1* on BTA4. SNPs *rs109658371* and *rs109901274* are the two lead SNPs on different chromosomes that explained more than 1% of phenotypic variance for REA. These two lead SNPs are located on BTA6 and BTA7, respectively. SNP *rs109658371* accounted for 3.32% of phenotypic variance for REA and is 221,454 bp away from gene *LCORL* while SNP *rs109901274* is a missense variant of gene *ARRDC3*, explaining 1.11% of phenotypic variance for REA. For LMY, SNPs *rs380838173* and *rs110302982* are the two lead SNPs with relatively larger effects. Both SNPs are located on BTA6, explaining 2.59 and 2.53% of phenotypic variance respectively. SNP *rs380838173* is 128,272 bp away from gene *LCORL* while SNP *rs110302982* is only 5080 bp away from gene *NCAPG*. For CMAR, two lead SNPs *rs211292205* and *rs441393071* on BTA1 explained 1.20 and 1.04% of phenotypic variance. SNP *rs211292205* is 50,986 bp away from gene *MRPS6* while SNP *rs441393071* was an intron SNP of gene *MRPS6.* The rest of the lead significant SNPs for CMAR accounted for less than 1% of phenotypic variance (Table 4).

**Table 4:** A summary of top lead SNPs of each chromosome in significant association with carcass merit traits based on imputed 7.8 M WGS variant GWAS with a threshold value of *P*-value < 10^−5^ in a beef cattle multibreed population

Trait^a^	Lead SNP	Num^b^	Chr	Pos (bp)	Nearest Gene^c^	Distance (bp)^d^	Annotation^e^	*P*-value	FDR^f^	b ± SE^g^	Var_Phe (%)^h^
HCW	rs467949024	3	2	94,243,607	ENSBTAG00000046783	41,253	intergenic_region	8.45E-06	4.95E-02	11.17 ± 2.51	0.62
HCW	Chr6:39111019	128	6	39,111,019	LCORL	118,907	intergenic_region	1.37E-22	1.74E-16	20.73 ± 2.12	4.79
HCW	rs109658371	185	6	39,213,566	LCORL	221,454	intergenic_region	2.98E-23	1.74E-16	20.2 ± 2.03	4.65
HCW	rs210782610	68	7	93,205,703	ARRDC3	34,716	intergenic_region	5.15E-08	7.80E-04	9.73 ± 1.79	1.10
HCW	rs380715719	27	8	18,612,164	TUSC1	42,366	intergenic_region	8.67E-06	5.03E-02	−7.54 ± 1.69	0.56
HCW	rs385024196	12	9	93,866,211	ENSBTAG00000035623	196,060	intergenic_region	9.20E-06	5.19E-02	11.06 ± 2.49	0.58
HCW	rs109815800	48	14	25,015,640	PLAG1	6344	intergenic_region	1.26E-21	5.82E-16	−21.9 ± 2.29	3.41
HCW	rs384702880	30	16	38,967,953	GORAB	43,016	intergenic_region	7.45E-06	4.53E-02	−8.73 ± 1.95	0.60
HCW	rs472775501	1	17	63,413,884	RASAL1	Within	intron_variant	1.59E-06	1.45E-02	18.73 ± 3.9	0.57
HCW	rs379088920	7	18	2,001,155	GLG1	Within	intron_variant	9.83E-06	5.49E-02	−12.84 ± 2.9	0.52
HCW	rs41934045	123	20	4,563,925	ERGIC1	Within	intron_variant	3.66E-11	1.23E-06	13.22 ± 2	1.47
HCW	Chr21:20514810	27	21	20,514,810	ENSBTAG00000001526	11,408	intergenic_region	4.92E-06	3.39E-02	−18.69 ± 4.09	0.60
HCW	rs110918739	26	26	19,498,886	HPS1	112,317	intergenic_region	3.91E-06	2.88E-02	−8.42 ± 1.83	0.65
HCW	rs452209056	0	28	29,895,281	CAMK2G	Within	intron_variant	8.09E-06	4.78E-02	20.57 ± 4.61	0.53
AFAT	rs42820451	14	3	77,060,742	DEPDC1	32,480	intergenic_region	9.75E-06	2.97E-01	−0.42 ± 0.09	0.72
AFAT	rs41594006	69	4	28,702,952	MACC1	133,040	intergenic_region	2.46E-07	1.77E-02	0.73 ± 0.14	1.07
AFAT	rs110995268	61	6	38,914,196	LCORL	Within	intron_variant	1.64E-12	9.39E-07	− 0.85 ± 0.12	2.87
AFAT	rs384948399	114	7	50,565,278	KLHL3	100,732	intergenic_region	5.70E-06	2.00E-01	0.67 ± 0.15	0.70
AFAT	rs380092738	12	8	45,378,038	PIP5K1B	23,538	intergenic_region	2.18E-06	9.46E-02	−0.6 ± 0.13	0.83
AFAT	rs209683528	96	8	45,076,648	FAM122A	100,581	intergenic_region	8.62E-06	2.75E-01	−0.72 ± 0.16	0.78
AFAT	rs470535700	0	11	102,954,409	SPACA9	Within	intron_variant	2.81E-06	1.15E-01	−0.62 ± 0.13	0.84
AFAT	rs209930593	84	13	63,970,531	RALY	Within	intron_variant	1.87E-06	8.29E-02	−0.64 ± 0.13	0.81
AFAT	rs134958846	142	14	24,894,463	LYN	Within	intron_variant	5.63E-06	2.00E-01	0.69 ± 0.15	0.82
AFAT	Chr15:50136986	11	15	50,136,986	ENSBTAG00000039298	3061	upstream_gene_variant	2.64E-06	1.09E-01	−1.15 ± 0.25	0.73
AFAT	rs381910687	26	16	24,333,881	RAB3GAP2	Within	downstream_gene_variant	5.89E-07	3.10E-02	−0.63 ± 0.13	0.98
AFAT	rs133531965	26	17	62,789,778	RBM19	96,955	intergenic_region	9.61E-06	2.95E-01	−0.44 ± 0.1	0.79
AFAT	rs464417711	8	28	5,603,922	U6	11,263	intergenic_region	3.43E-06	1.31E-01	1.96 ± 0.42	0.66
AFAT	rs714693579	62	29	32,805,714	KCNJ1	18,117	intergenic_region	4.63E-06	1.68E-01	−0.9 ± 0.2	0.70
REA	rs110874471	101	2	6,210,115	MSTN	3451	upstream_gene_variant	6.05E-07	5.04E-03	1.33 ± 0.27	0.80
REA	rs109890976	184	4	80,815,147	SUGCT	51,830	intergenic_region	3.95E-06	2.87E-02	1.63 ± 0.35	0.66
REA	rs109658371	164	6	39,213,566	LCORL	221,454	intergenic_region	3.05E-17	2.39E-10	2.37 ± 0.28	3.32
REA	rs109901274	78	7	93,244,933	ARRDC3	Within	missense_variant	5.28E-08	9.39E-04	1.36 ± 0.25	1.11
REA	rs109777279	33	9	57,219,389	ENSBTAG00000003743	296,654	intergenic_region	1.42E-06	1.09E-02	1.98 ± 0.41	0.74
REA	rs381345179	54	13	69,321,377	ENSBTAG00000045562	285,750	intergenic_region	6.28E-06	4.40E-02	1.72 ± 0.38	0.59
REA	rs135551190	24	14	24,977,053	MOS	105	upstream_gene_variant	4.40E-07	3.98E-03	−1.76 ± 0.35	0.83
REA	rs208370128	6	23	49,370,473	LYRM4	Within	intron_variant	2.30E-07	2.60E-03	2.3 ± 0.44	0.83
REA	Chr29:47488685	5	29	47,488,685	CCND1	55,695	intergenic_region	5.66E-07	4.84E-03	1.62 ± 0.32	0.70
LMY	rs383507504	64	1	58,167,076	GTPBP8	492	downstream_gene_variant	2.91E-06	1.27E-01	0.49 ± 0.1	0.79
LMY	rs136199724	22	2	123,199,149	PUM1	2620	downstream_gene_variant	6.29E-06	2.00E-01	0.72 ± 0.16	0.67
LMY	Chr3:46944817	50	3	46,944,817	PTBP2	34,161	intergenic_region	1.56E-06	7.69E-02	−0.5 ± 0.1	0.97
LMY	rs41594006	82	4	28,702,952	MACC1	133,040	intergenic_region	1.48E-06	7.37E-02	−0.67 ± 0.14	0.94
LMY	rs380838173	65	6	39,120,384	LCORL	128,272	intergenic_region	3.25E-11	2.92E-05	0.79 ± 0.12	2.59
LMY	rs110302982	339	6	38,760,889	NCAPG	5080	intergenic_region	4.71E-11	2.92E-05	0.78 ± 0.12	2.53
LMY	rs109722048	26	7	94,363,721	7SK	163,407	intergenic_region	4.27E-06	1.56E-01	0.47 ± 0.1	0.75
LMY	rs380092738	0	8	45,378,038	PIP5K1B	23,538	intergenic_region	1.90E-06	9.01E-02	0.6 ± 0.12	0.84
LMY	rs381625716	32	10	4,416,146	TMED7	Within	intron_variant	6.03E-06	1.98E-01	0.75 ± 0.17	0.68
LMY	rs41704822	21	13	64,131,777	EIF2S2	62,477	intergenic_region	7.72E-06	2.22E-01	−0.44 ± 0.1	0.90
LMY	rs379496842	24	14	25,350,856	PENK	127,865	intergenic_region	9.22E-06	2.40E-01	−0.6 ± 0.14	0.63
LMY	rs381910687	24	16	24,333,881	RAB3GAP2	Within	downstream_gene_variant	1.38E-06	7.08E-02	0.6 ± 0.12	0.92
LMY	rs446854454	0	17	71,415,918	LIF	Within	intron_variant	9.66E-07	5.38E-02	1.58 ± 0.32	0.68
LMY	rs209255508	3	20	36,664,583	GDNF	8055	intergenic_region	5.03E-06	1.75E-01	−0.82 ± 0.18	0.77
LMY	rs207913354	32	24	61,430,674	TNFRSF11A	155,480	intergenic_region	8.01E-06	2.22E-01	−0.43 ± 0.1	0.68
LMY	rs714693579	70	29	32,805,714	KCNJ1	18,117	intergenic_region	6.22E-08	6.03E-03	1.03 ± 0.19	0.96
CMAR	rs211292205	32	1	618,934	MRPS6	50,986	intergenic_region	4.40E-10	3.22E-03	10.63 ± 1.7	1.20
CMAR	rs441393071	62	1	724,086	MRPS6	Within	intron_variant	4.89E-09	5.12E-03	17.04 ± 2.9	1.04
CMAR	rs439430086	23	2	47,341,262	KIF5C	Within	intron_variant	2.35E-07	3.93E-02	−15.87 ± 3.07	0.96
CMAR	rs378618208	146	5	93,931,571	MGST1	Within	intron_variant	5.17E-06	2.17E-01	9.84 ± 2.16	0.74
CMAR	rs440019287	5	7	76,590,855	5S_rRNA	561,482	intergenic_region	2.04E-06	1.32E-01	−16.96 ± 3.57	0.65
CMAR	rs137214938	50	7	93,217,990	ARRDC3	21,912	intergenic_region	7.56E-06	2.79E-01	8.09 ± 1.81	0.57
CMAR	rs483021344	0	10	95,272,095	ENSBTAG00000018039	158,321	intergenic_region	3.90E-06	1.89E-01	−16.78 ± 3.63	0.60
CMAR	rs472692192	30	11	40,899,992	FANCL	155,573	intergenic_region	8.25E-07	9.54E-02	18.32 ± 3.72	0.73
CMAR	rs207650107	59	13	79,174,479	PTPN1	89,039	intergenic_region	7.17E-07	8.79E-02	10.1 ± 2.04	0.78
CMAR	rs382677800	119	15	37,550,837	ENSBTAG00000048131	40,352	intergenic_region	4.38E-06	2.06E-01	−8.9 ± 1.94	0.67
CMAR	rs454770498	45	17	22,076,614	SNORA25	8202	intergenic_region	9.92E-06	3.05E-01	9.33 ± 2.11	0.61
CMAR	rs137224539	35	18	29,266,132	ENSBTAG00000045255	Within	splice_region_variant&non_coding_exon_variant	5.05E-06	2.15E-01	15.06 ± 3.3	0.62
CMAR	rs43729456	10	19	42,229,279	KRT34	Within	intron_variant	9.66E-06	3.04E-01	11.88 ± 2.68	0.57
CMAR	rs379945647	6	20	20,934,339	U6	33,805	intergenic_region	5.04E-06	2.15E-01	−10.96 ± 2.4	0.72
CMAR	rs719382346	175	22	59,998,321	GATA2	18,664	intergenic_region	3.15E-06	1.73E-01	17.76 ± 3.81	0.74
CMAR	rs42610694	34	26	11,625,615	KIF20B	157,906	intergenic_region	6.04E-06	2.38E-01	14.7 ± 3.25	0.62

^a^*HCW* hot carcass weight in kg, *AFAT* average backfat thickness in mm, *REA* rib eye area in cm^2^, *LMY* lean meat yield in %, *CMAR* carcass marbling score from 100 (trace marbling) to 499 (more marbling). ^b^The number of significant support SNPs associated with a lead SNP within 70 k bps. ^c^The nearest annotated gene to the significant SNP. The annotated gene database was downloaded from https://www.ensembl.org/index.html. ^d^SNP designated as in a gene or distance (bp) from a gene region in the UMD3.1 bovine genome assembly. ^e^Functional annotation for the SNP. ^f^FDR = genome-wise false discovery rate (FDR) calculated from the Benjamini-Hochberg procedure [27]. ^g,h^The estimated allelic substitution effect (b) ± standard error (SE) and phenotypic variance explained by the significant SNP, respectively

### Enriched molecular and cellular and gene network

With a window of 70kbp extending upstream and downstream of each of the lead SNPs at FDR < 0.10, 319 candidate genes for HCW, 189 for AFAT, 575 for REA, 329 for LMY, and 198 for CMAR were identified based on annotated *Bos taurus* genes (23,431 genes on autosomes in total) that were downloaded from the Ensembl BioMart database (accessed on 8 November, 2018) (Additional file 1: Figure S4b). Of the identified candidate genes, 308, 180, 557, 318, and 188 genes were mapped to IPA knowledge base for HCW, AFAT, REA, LMY, and CMAR, respectively. In total, we identified 26 enriched molecular and cellular functions for AFAT, CMAR, and REA, and 25 functions for HWC and LMY at a *P*-value < 0.05 as presented in Additional file 1: Figure S2. Of all the five traits, lipid metabolism was among the top five molecular and cellular functions for AFAT, REA, LMY, and CMAR. For HCW, lipid metabolism was the sixth highest biological function involving 46 of the candidate genes. Across the five traits, the lipid related genes are primarily involved in the synthesis of lipid, metabolism of membrane lipid derivatives, concentration of lipid, and steroid metabolism processes as shown in the gene-biological process interaction networks (Additional file 1: Figure S3). Interestingly 18 genes involved in lipid synthesis including *ACSL6, CFTR, NGFR, ERLIN1, TFCP2L1, PLEKHA3, ST8SIA1, PPARGC1A, MAPK1, PARD3, PLA2G2A, AGMO, MOGAT2, PIGP, PIK3CB, NR5A1, CNTFR,* and *BMP7* are common for all the four traits. It is also worth noting that 18 (*AGMO, BID, BMP7, CFTR, CLEC11A, GNAI1, MOGAT2, MRAS, NGFR, NR5A1, P2RY13, PDK2, PIK3CB, PLA2G2A, PPARGC1A, PPARGC1B, PTHLH, and ST8SIA1*) of the 31 genes involved in lipid metabolism for AFAT have roles in lipid concentration.

Additionally, our results also revealed small molecular biochemistry and carbohydrate metabolism as other important molecular and cellular processes for AFAT, CMAR, HCW, and LMY (Additional file 1: Figure S3). Some of the major enriched subfunctions or biological processes related to carbohydrate metabolism included uptake of carbohydrate, synthesis of carbohydrate, and synthesis of phosphatidic acid as shown in the gene-biological process interaction networks (Additional file 1: Figure S3). For REA, cell morphology, cellular assembly and organization, cellular function and maintenance are the top enriched molecular processes in addition to lipid metabolism and molecular transport. The major enriched biological processes and subfunctions related within cell morphology function included transmembrane potential, transmembrane potential of mitochondria, morphology of epithelial cells, morphology of connective tissue cells, and axonogenesis as presented in (Additional file 1: Figure S3). For cellular function and maintenance, the genes are mainly involved in organization of cellular membrane, axonogenesis, the function of mitochondria, and transmembrane potential of the cellular membrane. The genes involved in these processes and subfunctions are also shown in Additional file 1: Figure S3. Table 5 lists all the genes involved in each of the top five enriched molecular processes for each trait while examples of the gene network for lipid metabolism and carbohydrate metabolism are presented in Additional file 1: Figure S3.

**Table 5:** Five topmost significantly enriched biological functions for carcass merit traits, and genes involved in the specific function

Trait^a^	Biological Function	Genes Involved in the biological function
HCW	Gene expression (23)	*BMP7, BTRC, CTCFL, DTX1, HIF3A, IRF9, KAT7, KDM8, LGALS1, MAPK1, MRAS, MS4A15, NFIA, NR5A1, PARD3, PCTP, PEG10, PPARGC1A, RNF4, RXRB, SIAH1, TADA3, TFCP2L1*
	Carbohydrate metabolism (26)	*AGMO, ALPI, BID, BMP7, CMAS, CYP2J2, FCGR2B, GRPR, KDM8, LGALS1, MAPK1, MRAS, NGFR, PARD3, PCTP, PDK2, PIGP, PIK3CB, PLA2G2A, PLEKHA3, PPARGC1A, PRKCB, PTHLH, ST8SIA1, UGT2B17, VDAC1*
	Nucleic acid metabolism (18)	*ADCY4, ATP5PF, BMP7, CFTR, CMAS, GART, GNAI1, GRPR, MAPK1, NUDT9, OLA1, PDK2, PPARGC1A, PRKCB, PTHLH, SLC25A5, ST8SIA1, VDAC1*
	Small molecule biochemistry (54)	*ACSL6, AGMO, AKR1C3, AKR1C4, ALPI, ANGPTL4, ATP5PF, BID, BMP7, CFTR, CLEC11A, CMAS, CNTFR, CYP2J2, DHRS4, ELOVL4, ERLIN1, FCGR2B, GBA3, GNAI1, GRPR, INHA, KCNE2, KCNE1B, LGALS1, MAPK1, MOGAT2, MRAS, NGFR, NR5A1, P2RY13, PARD3, PCCB, PCSK2, PCTP, PDK2, PIGP, PIK3CB, PLA2G2A, PLEKHA3, PPARGC1A, PPARGC1B, PRKCB, PTHLH, RXRB, SLC22A6, ST8SIA1* *TFCP2L1, TGM1, TTR, UGT2B11, UGT2B17, UPK2, VDAC1*
	Molecular transport (45)	*ACSL6, AGMO, ALPI, ANGPTL4, ATP10A, ATP6V1E1, ATP6V1G1, BID, BMP7, CA4, CCS, CFTR, CLEC11A, CLIC4, CNTFR, COQ7, FCGR2B, GNAI1, GRPR, HBA1/HBA2, INHA, KCNE2, KCNE1B, KCNK2, LGALS1, MAPK1, MOGAT2, MRAS, NGFR, NR5A1, P2RY13, PCTP, PDK2, PIK3CB, PLA2G2A, PPARGC1A, PPARGC1B, PRKCB, PTHLH, SLC20A2, SLC22A6, ST8SIA1, TTR, UPK2, VDAC1*
AFAT	Carbohydrate metabolism (22)	*AGMO, BID, BMP7, CMAS, GRPR, KDM8, LGALS1, MAPK1, MRAS, NGFR, PARD3, PDK2, PIK3CB, PLA2G2A, PPARGC1A, PPARGC1B, PTHLH, ST8SIA1, UGT2B17*
	Nucleic acid metabolism (10)	*BID, BMP7, CMAS, GART, GNAI1, GRPR, MAPK1, PDK2, ST8SIA1, UGT2B17*
	Small molecule biochemistry (36)	*ACSL6, AGMO, BID, BMP7, CFTR, CLEC11A, CMAS, CNTFR, DHRS4, ERLIN1, GART, GBA3, GNAI1, GRPR, KDM8, LGALS1, MAPK1, MOGAT2, MRAS, NGFR, NR5A1, P2RY13, PARD3, PDK2, PIGP, PIK3CB, PLA2G2A, PLEKHA3, PPARGC1A, PPARGC1B, PTHLH, SLC22A6, ST8SIA1, TFCP2L1, TGM1, UGT2B17*
	Lipid metabolism (31)	*ACSL6, AGMO, BID, BMP7, CFTR, CLEC11A, CNTFR, DHRS4, ERLIN1, GBA3, GNAI1, LGALS1, MAPK1, MOGAT2, MRAS, NGFR, NR5A1, P2RY13, PARD3, PDK2, PIGP, PIK3CB, PLA2G2A, PLEKHA3, PPARGC1A, PPARGC1B, PTHLH, ST8SIA1, TFCP2L1, TGM1, UGT2B17*
	Cell morphology (25)	*BID, BMP7, BTRC, CFTR, CLEC11A, CLIC4, CNTFR, FSCN1, GDF3, KCNK2, LGALS1, MAPK1, MRAS, NDUFAB1, NGFR, NR5A1, PCSK2, PLA2G2A, PLXNB2, PPARGC1A, PPARGC1B, PTHLH, SERPINA3, ST8SIA1, UPK2*
REA	Cell morphology (71)	*BID, CAMP, CCND1, CD4, CERS5, CFTR, CHL1, CLEC11A, CLIC4, CNTFR, CSTB, CUL3, DVL1, EPO, FGL1, GDF3, GSDMD, HAND1, HAUS4, HELLS, INHA, INTU, KCNK2, KIF11, KIFC1, LGALS1, LIF, LIMK2, MAPK1, MAPT, NDUFAB1, NEFH, NFIA, NGFR, NTRK2, OSMR, P2RY12, PALLD, PCTP, PEG10, PLXNB2, PPARGC1A, PPARGC1B, PTHLH, PTPN1, RNF4, SCYL1, SERPINA3, UCP1, UPK2*
	Cellular assembly and organization (58)	*AMPH, ARHGAP32, ARPC4, ATG4B, ATG4C, ATL1, BID, CAMP, CBLB, CCND1, CD4, CFTR, CLEC11A, CLIC4, CLTB, CSTB, CTDNEP1, DRP2, DVL1, EPO, EXO5, HAND1, IDE, KCNK2, KIF11, KIF13B, KIFC1, KLHDC8B, LANCL1, LGALS1, LIF, MAPT, NDUFAB1, NDUFS2, NEFH, NFIA, NGFR, NLGN1, NR5A1, NTRK2, OLA1, P2RY12, PALLD, PARD3, PLXNB2, POLG, PPARGC1A, PPARGC1B, REPS2, SERPINA3, SLC25A5, SNX9, SRCIN1, TP53INP1, TRAK2, TTR, UCP1, VDAC1*
	Cellular function and maintenance (51)	*ARHGAP32, ARMC4, ATL1, BID, CAMP, CCDC103, CCDC39, CCND1, CD4, CELSR2, CLEC11A, CLIC4, COQ7, CSTB, DVL1, EPO, FCGR2B, HAND1, IDE, IFNA2, KCNK2, KIF11, KIF13B, KIFC1, LANCL1, LGALS1, LIF, MAPT, NDUFAB1, NDUFS2, NEFH, NFIA, NGFR, NLGN1, NMNAT3, NTRK2, PARD3, PLXNB2, POLG, PPARGC1A, PPARGC1B, SCYL1, SERPINA3, SS18, ST8SIA1, TCF7L1, TFCP2L1, TP53INP1, TRAK2, UCP1, VDAC1*
	Lipid metabolism (77)	*ABHD3, ACSL6, AGMO, AKR1C3, AKR1C4, AKR1C1/AKR1C2, ALPI, ANGPTL4, ANGPTL6, ATP5PF, BID, BMP7, C3AR1, CAMP, CD4, CERS5, CFTR, CLDN16, CLEC11A, CNTFR, CTDNEP1, CYP2C18, CYP2J2, CYP7B1, DEGS2, DHRS4, ELOVL4, EPO, ERLIN1, FCGR2B, FGL1, GBA3, GNAI1, GPC3, IL1RN, INHA, KCNE1B, KIF13B, KLF15, LGALS1, LIF, MAPK1, MAPT, MOGAT2, MRAS, NGFR, NONO, NR5A1, NTRK2, OSMR, P2RY12, P2RY13, PARD3, PCTP, PDK2, PIGP, PIK3CB, PLA2G2A, PLEKHA3, POLG, PPARGC1A, PPARGC1B, PRKCB, PTHLH, PTPN1, RENBP, RGS2, RXRB, SERPINE2, ST8SIA1, TFCP2L1, TRHR, TTR, UCP1, UGT2B4, UGT2B11, UGT2B17*
	Molecular transport (105)	*ACSL6, AGMO, AKR1C4, AKR1C1/AKR1C2, ALPI, ANGPTL4, ANGPTL6, AOC3, APPBP2, ATP10A, ATP6V1E1, ATP6V1G1, BID, BMP7, C3AR1, CA4, CAMP, CBLB, CCS, CD4, CERS5, CFTR, CLDN16, CLEC11A, CLIC4, CNTFR, COQ7, CTDNEP1, DIO3, DUOXA2, DVL1, ELOVL4, EPO, FCGR2B, FGL1, GCNT4, GNAI1, GPC3, GRPR, HBA1/HBA2, IL1RN, INHA, IP6K1, KCNAB1, KCNE2, KCNE1B, KCNK2, KDM8, KIF13A, KIF13B, KLF15, LGALS1, LIF, MAPK1, MAPT, MOGAT2, MRAS, NDC1, NGFR, NONO, NR5A1, NTRK2, OGG1, OSMR, P2RY12, P2RY13, PCSK2, PCTP, PDK2, PIK3CB, PKN1, PLA2G2A, POLG, PPARGC1A, PPARGC1B, PRKCB, PTGER1, PTHLH, PTPN1, RENBP, RXRB, SCN9A, SLC16A4, SLC20A2, SLC22A6, SLC37A2, SLC39A7, SLC6A7, SLC8B1, SMG6, SNX9, SRCIN1, ST8SIA1, STRADA, STRADB, SVBP, SYNDIG1, TMED2, TP53INP1, TRAK2, TRHR, TTR, UCP1, VDAC1, ZFP36L1*
LMY	Gene expression (23)	*BMP7, BTRC, CTCFL, DTX1, HIF3A, IRF9, KAT7, KDM8, LGALS1, MAPK1, MRAS, MS4A15, NFIA, NR5A1, PARD3, PCTP, PEG10, PPARGC1A, RNF4, RXRB, SIAH1, TADA3, TFCP2L1*
	Lipid metabolism (47)	*ACSL6, AGMO, AKR1C3, AKR1C4, ALPI, ANGPTL4, ATP5PF, BID, BMP7, CFTR, CLEC11A, CNTFR, CYP2J2, DHRS4, ELOVL4, ERLIN1, FCGR2B, GBA3, GNAI1, INHA, KCNE1B, LGALS1, LIF, MAPK1, MOGAT2, MRAS, NGFR, NR5A1, P2RY13, PARD3, PCCB, PCTP, PDK2, PIGP, PIK3CB, PLA2G2A, PLEKHA3, PPARGC1A, PPARGC1B, PRKCB, PTHLH, RXRB, ST8SIA1, TFCP2L1, TTR, UGT2B11, UGT2B17*
	Small molecule biochemistry (55)	*ACSL6, AGMO, AKR1C3, AKR1C4, ALPI, ANGPTL4, ATP5PF, BID, BMP7, CFTR, CLEC11A, CMAS, CNTFR, CYP2J2, DHRS4, ELOVL4, ERLIN1, FCGR2B, GBA3, GNAI1, GRPR, INHA, KCNE1B, KCNE2, LGALS1, LIF, MAPK1, MOGAT2, MRAS, NGFR, NR5A1, P2RY13, PARD3, PCCB, PCSK2, PCTP, PDK2, PIGP, PIK3CB, PLA2G2A, PLEKHA3, PPARGC1A, PPARGC1B, PRKCB, PTHLH, RXRB, SLC22A6, ST8SIA1, TFCP2L1, TGM1, TTR, UGT2B11, UGT2B17, UPK2, VDAC1*
	Vitamin and mineral metabolism (17)	*AKR1C3, AKR1C4, BMP7, CFTR, CYP2J2, DHRS4, INHA, LIF, NR5A1, P2RY13, PCTP, PPARGC1A, ST8SIA1, TTR, UGT2B11, UGT2B17, VDAC1*
	Carbohydrate metabolism (26)	*AGMO, ALPI, BID, BMP7, CMAS, CYP2J2, FCGR2B, GRPR, KDM8, LGALS1, MAPK1, MRAS, NGFR, PARD3, PCTP, PDK2, PIGP, PIK3CB, PLA2G2A, PLEKHA3, PPARGC1A, PRKCB, PTHLH, ST8SIA1, UGT2B17, VDAC1*
CMAR	Carbohydrate Metabolism (23)	*AGMO, BID, BMP7, CMAS, GNAI1, GRPR, KDM8, LGALS1, MAPK1, MRAS, NGFR, PARD3, PCTP, PDK2, PIGP, PIK3CB, PLA2G2A, PLEKHA3, PPARGC1A, PPARGC1B, PTHLH, ST8SIA1, UGT2B17*
	Nucleic acid metabolism (10)	*BID, BMP7, CMAS, GART, GNAI1, GRPR, MAPK1, PDK2, ST8SIA1, UGT2B17*
	Small molecule biochemistry (40)	*ACSL6, AGMO, AKR1C3, AKR1C4, BID, BMP7, CFTR, CLEC11A, CMAS, CNTFR, DHRS4, ERLIN1, GART, GBA3, GNAI1, GRPR, KDM8, LGALS1, MAPK1, MOGAT2, MRAS, NGFR, NR5A1, P2RY13, PARD3, PCSK2, PCTP, PDK2, PIGP, PIK3CB, PLA2G2A, PLEKHA3, PPARGC1A, PPARGC1B, PTHLH, SLC22A6, ST8SIA1, TFCP2L1, TGM1, UGT2B17*
	Cellular development (24)	*AKR1C3, B9D1, BID, BMP7, CBLB, CLEC11A, CLIC4, FSCN1, ITGA11, ITIH4, KCNK2, LGALS1, MAPK1, MRAS, NASP, NGFR, NR5A1, PIK3CB, PPARGC1A, PTHLH, TGM1, UGT2B17, UPK2, ZFP36L1*
	Lipid metabolism (33)	*ACSL6, AGMO, AKR1C3, AKR1C4, BID, BMP7, CFTR, CLEC11A, CNTFR, DHRS4, ERLIN1, GBA3, GNAI1, LGALS1, MAPK1, MOGAT2, MRAS, NGFR, NR5A1, P2RY13, PARD3, PCTP, PDK2, PIGP, PIK3CB, PLA2G2A, PLEKHA3, PPARGC1A, PPARGC1B, PTHLH, ST8SIA1, TFCP2L1, UGT2B17*

^a^*HCW* hot carcass weight in kg, *AFAT* average backfat thickness in mm, *REA* rib eye area in cm^2^, *LMY* lean meat yield in %, *CMAR* carcass marbling score from 100 (trace marbling) to 499 (more marbling)

## Discussion

### The value of the imputed 7.8 M whole genome sequence variants on GWAS

With the 50 K SNPs (*N* = 30,155) as the base genotypes, a reference population of 4059 animals of multi-breeds genotyped with the Affymetrix HD panel, and a panel of 1570 animals with WGS variants from run 5 of the 1000 Bull Genomes Project, we achieved an average imputation accuracy of 96.41% on 381,318,974 whole genotype sequence variants using FImpute 2.2 [28]. This average imputation accuracy is comparable to the imputation accuracy previously obtained in beef cattle [29] but slightly lower than that in dairy cattle [30, 31]. However, the imputation accuracy over a validation dataset of 240 animals varied among individual DNA variants, with a range from 0.42 to 100% (data not shown). To ensure a higher quality of imputed WGS DNA variants, we removed imputed WGS DNA variants with an average imputation accuracy less than 95% of the 5-fold cross-valuation at each individual DNA variant, MAF < 0.5%, and deviation from HWE at *P*-value < 10^− 5^, leaving 7,853,211 DNA variants for GWAS. With this WGS DNA panel, we demonstrated that the additive genetic variance and corresponding heritability estimates increased by 6.1 to 22.5% for all the five carcass traits in comparison to the 50 K SNP panel (Table 1). Moreover, the 7,853,211 DNA variant based GWAS identified additional significant DNA variants for all five carcass merit traits in comparison to the 50 K SNPs. In a companion study, we also observed that the imputed 7.8 M DNA variants accounted for more additive genetic variance and led to identification of additional DNA variants that are associated with feed efficiency and growth traits in beef cattle [23], indicating that the imputed 7.8 M WGS variants can improve the power of GWAS analyses for beef cattle quantitative traits.

### DNA marker effect distributions

The distributions of DNA marker allele substitution effects and the amounts of additive genetic variances explained by single DNA markers support the assumptions of a normal distribution for SNP effects and a scaled inverse-chi squared distribution for SNP additive genetic variance used in previous studies [32, 33], although these DNA marker effect distributions may be biased as greater LD between DNA markers in the 7.8 M DNA variant panel is expected and a single DNA marker GWAS was used in this study. The 7.8 M DNA variant GWAS also demonstrated that the majority of the variants had zero or near zero effects on all the five carcass merit traits, and only a small fraction (< 0.1%) of the 7,853,211 WGS variants passed the suggestive threshold of *P*-value < 0.005. This seems to correspond well to a π value of approximately 99% that was commonly used as an assumption to shrink proportions of SNPs to no effects in genetic analyses with high density SNPs [34]. Another important aspect of quantitative trait genetic architecture is whether the trait is affected by many genes with small effects or by a few of genes with large and/or modest effects plus genes with small effects. The GWAS results based on the 7.8 M DNA variants showed that HCW, LMY, and REA are likely influenced by a few of genes with larger effects, explaining up to 4.79% phenotypic variance, and many genes with small effects. However, for AFAT and CMAR, a few of genes with modest effects and many genes with small effects likely contribute to the variation of the traits (Table 4).

### DNA marker effects related to SNP functional classes

Annotation of DNA variants into functional classes allows for further dissection of DNA marker effects on the trait to DNA variant functionality. The imputed 7.8 M DNA variants include a proportionally larger number of DNA polymorphisms in each of the functional classes, ranging from 3309 for 5’UTR variants to 5,251,680 for intergenic region in comparison to the lower density SNP panels such as the bovine 50 K SNPs, which was reported in the Additional files of Zhang et al. (2019) [23]. For convenience, the annotation information of various DNA variant panels has been provided in Additional file 3: Tables S1–S3 of this article. We used both the average squared allele substitution effects of each functional class and the additive genetic variance captured by a single DNA variant within the functional class to assess their relative importance in affecting the trait. For the average squared allele substitution effects, missense variants, 3’UTR, 5’UTR, and other regulatory region variants exhibited a relatively larger allele substitution effect on all five carcass merit traits in general in comparison to variants in other functional classes. Although the LD between DNA markers of different functional classes and the singe DNA marker GWAS used in this study may lead to biased estimates of the DNA marker effect on the traits, the results are in agreement with the expectation that missense variants alter the peptide sequence of a protein, and greater roles of 3’UTR, 5’UTR, and other regulatory variants play in influencing gene expression and gene translation [35–37].

To provide further insight into relative importance of each functional class, we fitted the GRM of the functional class and GRM constructed from DNA variants of all other functional classes simultaneously to estimate the additive genetic variance captured by each functional class. For each functional class, the sum of the additive genetic variances captured by the two GRMs (Table 3) was almost the same as the additive genetic variance obtained by the GRM with all the imputed 7.8 M DNA variants for all the traits (Table 1), indicating a reliable partition of additive genetic variance for each function class variants for the carcass merit traits. Although intergenic variants and intron variants captured a greater amount of total additive genetic variance for all five carcass merit traits, their relative proportion of additive genetic variance explained per sequence variant was smaller than other functional classes. These results concur with the report by Koufariotis et al. [38] that the intron and intergenic variants explained the lowest proportion of the genetic variance per SNP for milk and fertility traits in dairy cattle. Relatively smaller amount of additive genetic variance captured per sequence variant in intron and intergenic regions were also observed for feed efficiency related traits in beef cattle [23], which is likely due to much larger numbers of DNA variants in the class and the majority of them have small or zero effects on the traits. Of other functional classes, 3’UTR explained more additive genetic variance per DNA variant for HCW, AFAT, and REA while DNA variants in 5’UTR and other regulatory variants also showed a greater amount of additive genetic variance explained per sequence variant for CMAR and for CMAR and REA, respectively. It was found that missense variants captured a greater amount of additive genetic variance per sequence variant for REA, LMY, and CMAR. Although synonymous variants had relatively small average squared SNP allele substitution effects, a single DNA variant in the functional class accounted for more additive genetic variance for AFAT, REA, LMY, and CMAR. In addition, both the downstream and upstream gene variants were found to capture more additive genetic variance per sequence variant for HCW (Table 3). These results suggest that the relative contribution per DNA variant of the functional classes to the additive genetic variance might vary across different traits. Indeed, in a study by Koufariotis et al. [39], functional classes including splice sites, 3’UTR, 5’UTR, and synonymous variants explained relatively a larger proportion of genetic variance per sequence variant for milk production traits but not for fertility related traits.

It was observed that most top lead SNPs with larger effects are located between genes or located in intronic regions, although their average SNP effects or variances captured by individual DNA variants were relatively smaller than missense and regulatory DNA variants including 3’UTR and 5’UTR variants (Table 4). However, there were cases for each trait where support SNPs had either larger allele substitution effects or explained a larger percentage of phenotypic variance than those of their lead SNPs but with a larger *P*-value. For instance, a missense variant *rs42661323* at 4,916,731 bp on BTA20 had an allele substitution effect of 10.73 on HCW, which was larger than that (*b* = 10.14) of its nearby lead SNP *rs41574252* located at 4863507 bp. However, the *P*-value of the missense variant *rs42661323* was 8.10 × 10^− 8^ and was slightly larger than that (*P*-value = 4.85 × 10^− 8^, or 4.85E-08) of its lead SNP (Additional file 2). A similar instance was found for missense variant *rs379314731* of gene *ENSBTAG00000012585 (RAB3GAP2)* at 24,332,917 bp on BTA16 for AFAT. The missense variant *rs379314731* had an allele substitution effect of − 0.64 on AFAT with a *P*-value of 8.76 × 10^− 7^. However, its nearby downstream gene SNP *rs381910687* was selected as the lead SNP due to its lower *P*-value of 5.89 × 10^− 7^ although its allele substitution effect was slightly smaller (i.e. b = − 0.63). Therefore, support SNPs that are located in more important functional classes such as missense and regulatory variants are also worth further investigation. Additionally, as the imputed 7.8 M DNA variants represent a proportion of whole genome DNA polymorphisms, the intergenic or intronic SNPs with larger effects may also be in LD with the causative DNA variant(s) that are not present in the 7.8 M DNA variant panel. In this case, fine mapping of QTL in the region of lead SNPs is needed to identify the causative DNA variants for the trait.

### QTLs for carcass merit trait in beef cattle

Mapping QTLs via linkage or association analyses are subject to a false positive rate. Therefore, validation of QTL or DNA variants associated with a trait in independent studies provides confidence on the identified candidate QTLs or DNA variants. We compared our lead significant SNPs with the QTL regions reported in the Cattle QTL database (https://www.animalgenome.org/cgi-bin/QTLdb/index, accessed on 22 August 2018) [14]. With a window centered at the lead SNPs extending 70 kb upstream and downstream, 33, 17, 20, 3, and 0 were overlapped with reported QTL for HCW, AFAT, REA, LMY, and CMAR, respectively (Additional file 3: Table S4). With a window of 1 Mb, 41 of the 51 lead SNPs for HCW, 20 of the 33 lead SNPs for AFAT, 31 of the 46 lead SNPs for REA, 15 of the 40 lead SNPs for LMY, and 2 of the 39 lead SNPs for CMAR were found to be overlapped with the reported QTL in the Cattle QTL database (Additional file 3: Table S4). These overlapped lead SNPs provide additional evidence that the QTL regions may harbor causative DNA variants affecting the carcass merit traits. The non-overlapped lead SNPs, however, may suggest unique QTLs that were segregating in the investigated beef cattle population for the trait, in particular for the lead SNPs with multiple support SNPs (Table 4).

To investigate potential pleotropic effects of SNPs or QTL regions on the carcass merit traits, we also compared lead significant SNPs among the five carcass merit traits. It was found that CMAR did not share any lead significant SNPs with HCW, AFAT, REA, or LMY. HCW, AFAT, REA, and LMY, however, shared a common significant lead SNP “*rs109696064*”, which was a downstream gene variant that is 3164 bps away from the nearest gene *LCORL* on chromosome 6 (Additional file 2). AFAT and HCW also shared four lead significant SNPs located on chromosome 6, including one intronic variant (*rs109355965*) that is within gene *ENSBTAG00000005932 (FAM184B),* one intronic variant (*rs110995268*) of gene *LCORL*, one downstream gene variant (*rs109843602*) that was in proximity to genes *NCAPG* and *DCAF16*, and one downstream gene variant (*rs109696064*) located within 70 kb of genes *LCORL* and *NCAPG*. The region that harbors genes *NCAPG*-*LCORL* on BTA6 is likely to be a candidate QTL region with pleiotropic effects for carcass merit traits including HCW, AFAT, REA, and LMY. The lead significant SNPs located on BTA 6 in the region of 37.9 Mb to 39.9 Mb were also found to have relatively larger effects on HCW, AFAT, REA, and LMY as shown in the Manhattan plots (Fig. 1). The chromosome region (i.e 6_37 to 6_39) was previously reported to have large pleiotropic effects on traits including carcass weight, rib eye muscle area, and carcass fat thickness in multiple US cattle breeds [40]. In our 7.8 M DNA variant GWAS for feed efficiency related traits, this chromosomal region also showed the largest effects on DMI, ADG, and MWT, explaining from 3.04 to 5.80% phenotypic variance for the traits as reported by Zhang et al. in our companion paper [23]. All these results strengthen the evidence that there are likely causative DNA variants in the chromosomal region with major pleiotropic effects on beef cattle growth related traits [40]. Genes *NCAPG* and *LCORL* are the two major nearest genes to the chromosomal region. DNA markers within or in proximity to genes *NCAPG* (Non-SMC Condensin I Complex Subunit) and *LCORL* (ligand-dependent nuclear receptor co-repressor like) were found to have significant associations with feed intake and body weight gain in beef cattle [41]. In our study, the annotation of the imputed 7.8 M DNA marker panel identified a total of 185 WGS variants within *NCAPG* including 4 synonymous variants, 177 intronic variants, 2 missense variants, and 2 other regulatory region variants. Also a total of 409 WGS variants were within gene *LCORL*, including 404 intronic variants, 1 missense variant, and 4 3’UTR variants. At *P*-value less than 10^− 5^, 17 SNPs (including 15 intronic variants and 2 missense variants) within gene *NCAPG* were found to be in significant association with HCW but none of them were identified to be a lead SNP. The intronic SNP *rs110175987* of *NCAPG* was significantly associated with HCW (i.e. AC_000163.1:g.38783305C > T, *P*-value = 1.14 × 10^− 19^ and FDR = 1.51 × 10^− 15^), accounting for 4.18% of the phenotypic variance, and it was the largest proportion of phenotypic variance explained by a single DNA marker among the 17 within-gene variants (Additional file 2). This SNP was also significantly associated with AFAT (*P*-value = 5.42 × 10^− 12^ and FDR = 1.33 × 10^− 6^), REA (*P*-value = 1.34 × 10^− 15^ and FDR = 3.99 × 10^− 10^) and LMY (*P*-value = 1.20 × 10^− 10^ and FDR = 2.92 × 10^− 5^), explaining 2.72, 3.19, and 2.41% of the phenotypic variance, respectively (Additional file 2). A missense variant *rs109570900* at 38,777,311 bp on BTA6, which induces a Ile-442-Met substitution in amino acid within *NCAPG*, was also identified to be in significant association with HCW (*P*-value = 2.10 × 10^− 9^ and FDR = 4.65 × 10^− 5^) and REA (*P*-value = 5.09 × 10^− 8^ and FDR = 9.39 × 10^− 4^) accounting for 1.45 and 1.18% of phenotypic variance, respectively. Previous studies reported that this missense variant had strong association with fetal growth and birth weight in Holstein and Charolais crossbreed [42]. The missense variant was also in association with body frame size at puberty in Japanese black and Charolais × Holstein [43] and with carcass weight, longissimus muscle area, and subcutaneous fat thickness in Japanese Black and Brown cattle [44]. Sahana et al. [45] proposed the missense as a strong candidate responsible for calf size at birth and consequently calf birth survival. In our companion paper by Zhang et al. [23], the SNP within *NCAPG* was also found to be associated with ADG, DMI, and MWT, respectively.

For gene *LCORL*, the intronic SNP *rs110995268* at 38,914,196 bp was significantly associated with AFAT (*P*-value = 1.64 × 10^− 12^ and FDR = 9.39 × 10^− 7^), explaining 2.87% of the phenotypic variance (Table 4). The SNP was also significantly associated with HCW (*P*-value = 4.2 × 10^− 20^ and FDR = 6.76 × 10^− 15^), REA (*P*-value = 1.80 × 10^− 15^ and FDR = 4.12 × 10^− 10^), and LMY (*P*-value = 6.15 × 10^− 11^ and FDR = 2.92 × 10^− 5^), explaining 4.33, 3.19, and 2.50% of the phenotypic variance, respectively (Additional file 2). A total of 80, 15, 47, and 15 SNPs within gene *LCORL* were identified to be significantly associated with HCW, AFAT, REA, and LMY respectively. However, they were all intronic variants. The intronic SNP *rs110995268* belongs to a group of 15 common significant intronic variants within *LCORL* that had effects on HCW, AFAT, REA, and LMY. Out of the 15 within-gene intronic SNPs, proportions of phenotypic variance ranged from 4.30 to 4.37% for HCW, from 2.82 to 2.87% for AFAT, from 3.10 to 3.24% for REA, and 2.44 to 2.50% for LMY (Additional file 2).

The *NCAPG-LCORL* region also encompassed two additional interesting genes including *DCAF16* and *FAM184B*. In addition, gene SNPs under other lead significant QTL regions with relatively larger effects were also examined and some significant lead SNPs were found to be missense or located within regulatory regions (Table 4), which may suggest their roles as causative mutations due to the functional annotation. For instance, a missense variant *rs109901274* within gene *ENSBTAG00000007116* (*ARRDC3*) at 93,244,933 bp on chromosome 7 was a lead SNP in significant association (*P*-value = 5.28 × 10^− 8^) with REA, explaining 1.11% of phenotypic variance (Table 4). The SNP rs109901274 was also found to be a significant support SNP in association with HCW, with a *P*-value of 8.84 × 10^− 8^ and accounted for 1.07% of phenotypic variance (Additional file 2). Gene *ARRDC3*, which harbours SNP *rs109901274*, belongs to an arrestin superfamily and plays a role in regulating body mass in mice [46] and human males [47]. In our companion paper by Zhang et al. [23], SNP *rs109901274* was also reported to be a lead SNP in significant association with ADG and MWT. A previously study by Saatchi et al. reported that SNPs in proximity to *ARRDC3* were associated with birth weight, carcass weights, and body weights in US cattle breeds [40]. However, the physiological roles of *ARRDC3* in cattle remain unknown.

It was commonly observed that SNPs from the intronic region of the genes or between genes showed significant effects on the carcass merit traits as lead SNPs. For instance, one of the most significant lead SNPs (*rs109815800*, AC_000171.1:g.25015640G > T, *P*-value = 1.26 × 10^− 21^ and FDR = 5.82 × 10^− 16^) in association with HCW on chromosome 14 at 25015,640 bp was mapped to the intergenic region (6344 bp upstream) of *PLAG1* (Table 4). This SNP was previously reported as one of the eight candidate QTNs with major effects on bovine stature by Karim et al. [48]. The SNP was also the most significant DNA marker reported by Fink et al. [49] in expression QTL mapping of *PLAG1*, and the most significant SNP in meta-analysis of GWAS for cattle stature by Bouwman et al. [50]. This SNP (i.e. *rs109815800*) accounted for 3.41% of phenotypic variance of HCW in this study (Table 4). Additionally, SNP *rs109815800* was a support SNP in significant association with REA, reaching a *P*-value of 2.02 × 10^− 6^ and explained 0.84% of phenotypic variance (Additional file 2). These intronic DNA variants significantly associated with the traits may also warrant further investigation for their effects on the traits. In addition, the significant intronic and intergenic DNA variants may also in high LD with the causative DNA variant(s) that are not present in the imputed 7.8 M DNA variant panel. Therefore, further fine mapping of the QTL regions will lead to identification of causative variants for the carcass merit traits in cattle, in particular for QTL regions where lead SNPs are supported by multiple significant DNA markers.

### Genetic networks compared with RNAseq

The IPA analyses based on the candidate genes identified via a window of 70 k bp of the lead SNPs with FDR < 0.10 detected lipid metabolism was among the top 5 enriched molecular process for four of the carcass merit traits (AFAT, CMAR, LMY, and REA), and 6th for HCW, followed by carbohydrate metabolisms and small molecule biochemistry. In studies using RNAseq on bovine liver samples, lipid metabolism, and small molecule biochemistry were also among the top enriched molecular processes for marbling score in Charolais steers [51, 52]. In this study, all the animals with carcass data were finished for meat production. The goal of the fattening stage with a finishing diet is to allow beef cattle to grow muscle and to accumulate intramuscular fat, i.e. marbling, for better carcass quality. Therefore, genes involved in lipid metabolism and carbohydrate metabolism likely play a more important role in determining the carcass merit traits, as shown both in this and previous studies [51, 52]. The identification of top and other enriched molecular processes and their corresponding genes will not only improve our understanding on genetic mechanisms that influence the carcass traits but also help prioritize candidate genes for identification of causative gene polymorphisms responsible for the phenotypic variation.

## Conclusions

The imputed 7,853,211 DNA variants explained more genetic variance than the 50 K SNP panel and led to identification of additional QTL regions in associations with carcass merit traits in Canadian multi-breed beef cattle. The DNA marker allele substitution effects on the carcass traits based on the imputed 7,853,211 DNA variants approximated a bell-shaped distribution, and the additive genetic variances explained by single DNA variants followed a scaled inverse chi-squared distribution to a greater extent. On average, missense variants, 3’UTR variants, 5’UTR variants, and other regulatory region variants exhibited larger allele substitution effects in comparison to DNA variants that are located between genes and in intronic regions. Intergenic and intronic variants also accounted for a smaller amount of additive genetic variance per DNA variant for the carcass traits whereas single regulatory, synonymous, and missense variants had relatively larger impacts on the variation of carcass merit traits. The five carcass merit traits appear to be controlled by a few DNA variants with relatively larger or modest effects complementary by DNA variants with small effects. Lipid metabolism, small molecular biochemistry, and carbohydrate metabolism were the top biological processes for the carcass merit traits. The genetic architecture as revealed by the 7.8 M DNA variant GWAS will improve our understanding on the genetic control of carcass merit traits in beef cattle.

## Methods

### Animal populations and phenotype data

The populations used in this study, i.e., Angus, Charolais, Kinsella Composite, Elora crossbred, PG1, and TXX, were described previously [23, 53–56]. Briefly, Angus, Charolais, and Kinsella Composite herds are located at Roy Berg Kinsella Research Ranch, University of Alberta, with Angus and Charolais being maintained as purebreds while the Kinsella Composite herd had been influenced mainly by Angus, Charolais, Galloway, and Hereford. The Elora crossbred animals were from the Elora Beef Research Centre, University of Guelph and it was made by crossing Angus, Simmental, Charolais, and other cattle breeds. Both the commercial crossbred PG1 and terminal crossbred TXX animals were from multiple commercial herds in Alberta. The top beef breeds that were used in commercial crossbred beef production in Alberta included Angus, Charolais, Herefore, Simmental, Limousin, Gelbvieh, while the TXX animals were produced from 2- or 3-way crossbreeding systems involving terminal composite bulls (TX/TXX) and crossbred cows of multiple beef breeds. Animals used in this study were finishing steers and heifers born between 1998 and 2006 for the Elora crossbred, between 2002 and 2015 for Kinsella Composite, between 2004 and 2015 for Angus and Charolais, between 2008 and 2011 for PG1 and TXX populations.

The animals were initially measured for feed intake using the GrowSafe system (GrowSafe Systems Ltd., Airdrie, Alberta, Canada) at their respective feedlot test station under multiple projects, which were described previously [55, 57–59]. After the feedlot tests, animals were slaughtered either at a commercial plant or at the Lacombe Research and Development Centre (LRDC) abattoir when a majority of them reached > 8 mm backfat thickness as predicted from ultrasound measurements. For slaughter, animals were first stunned by captive bolt and then exsanguinated. Collection of carcass data was previously described [53, 55, 59–62]. Briefly, hot carcass weight (HCW) in kg was obtained by summing up the weight of each side of the carcass that was split during dressing, about 45 min post-mortem. Average backfat thickness (AFAT) in mm, rib eye area (REA) in squared centimeters, and carcass marbling score (CMAR) at the grading site between the 12th and 13th ribs was assessed by trained personnel. Carcass marbling score was measured as a continuous variable from 100 (trace marbling or less) to 499 (abundant or more marbling) to reflect the amount of fat deposit interspersed between the muscle fibers (i.e., intramuscular fat) of the longissimus thoracis. Lean meat yield (LMY) was calculated as LMY, % = 57.96 + (0.202 × REA, cm^2^) − (0.027 × HCW, kg) − (0.703 × AFAT, mm) as described by Basarab et al. [57] as an estimate of saleable meat in the carcass. The phenotype data obtained from each data source were examined and phenotypic values beyond 3 standard deviations of the trait value mean were excluded from further analyses.

### SNP data consolidation, imputation, and functional annotation

All animals entering the feedlot tests were genotyped with bovine 50 K SNP panels under multiple projects. SNP data consolidation and imputation was described in the companion paper [23]. Briefly, raw 50 K SNP genotype profile data were obtained from each source and SNP genotypes were then called in each of the four different SNP formats, i.e. forward strand, top strand, design strand, and AB format. The SNP genotype data were then combined by the same SNP format and each SNP was examined to ensure it had only two alleles after merging. In total, 50 K SNP genotypes of 11,448 beef cattle were compiled. A SNP quality check was applied for each data source, where SNPs that had a minor allele frequency less than 5%, or had a missing rate larger than 5%, or were significantly deviated from exact test of Hardy-Weinberg equilibrium (HWE) (*P*-value < 10^− 3^), or on sex chromosomes were filtered out. SNPs removed from one data source were also excluded from all other data sources. In addition, animals with more than a 5% missing rate of total SNP genotypes were deleted. After SNP data editing, 33,321 SNPs were retained for further analyses. Sporadic missing SNP genotypes in the SNP data set (< 0.065%) were then imputed via the population-based algorithm implemented in Beagle 3.3.2 [63]. Population admixture analyses were also conducted for all the 11,448 beef cattle based on the 33,321 SNPs to predict breed composition for each animal, which was described in the companion paper [23].

SNP imputation was conducted using FImpute 2.2 [28] in a two-step procedure: (1) from the 50 K SNPs (i.e. 33,321 SNPs) to the Affymetrix Axiom Genome-Wide BOS 1 Array (Affymetrix, Inc., Santa Clara); (2) from imputed HD to the full whole-genome sequence (WGS) variants in run 5 of the 1000 Bull Genomes Project [22]. Details of SNP imputation and average imputation accuracy for each chromosome were provided in the companion paper [23]. Initially, 38,318,974 imputed WGS variant genotypes were obtained for all the animals. Quality control was then performed on the imputed WGS variant genotypes to ensure better quality of imputed genotype data, where DNA variant genotypes with less than 95% imputation accuracy, or being homozygous, or with a minor allele frequency (MAF) less than 0.005 in either population/breed, or with significant deviations from Hardy–Weinberg exact test at significance levels of *P-*value < 10^− 5^ in either population/breed were excluded from further analyses. The post-imputation quality control resulted in 7,853,211 DNA variant genotypes that contain 30,155 SNPs from the 50 K SNP genotypes on all the animals. The 7,853,211 DNA variants included 7,497,128 SNPs and 356,083 INDELs (termed 7.8 M DNA variants or 7.8 M DNA variant panel or 7.8 M SNP panel in the text). The imputed 30,155 SNPs in the 7.8 M DNA variant panel were replaced by their actual genotypes to facilitate comparison of the 50 K SNP panel and the 7.8 M DNA variants panel.

Functional annotation of SNPs or DNA variants on the 30,155 SNPs and on the 7,853,211 DNA variants was provided in the companion paper [23]. The WGS DNA variants were annotated through run 5 of the 1000 Bull Genomes Project, which included 379 full genome sequences from the Canadian Cattle Genome Project [64]. DNA variants were then assigned to a functional class based on their overlap with gene features described in the Ensembl database (release 81), using an updated version of the NGS-SNP annotation system [65]. These SNPs were grouped into 9 broader functional classes, which consisted of intergenic region variants, downstream gene variants, upstream gene variants, synonymous variants, intron variants, missense variants, 3′ UTR variants, 5′ UTR variants, and other regulatory region variants that includes splice regions in intron variants, disruptive in-frame deletion, and splice region variants, etc. (Additional file 3: Table S1–S3).

### Genome wide association analyses

Animals with carcass data were merged with their imputed genotype data in the 7.8 M DNA variant panel, resulting in a sample size of *n* = 3354 for AFAT to *n* = 3984 for HCW (Table 1). For the GWAS analyses, phenotypic values of the five carcass traits were adjusted for animal birth year, sex type, a combination of feedlot test location and pen, breed composition fraction of each postulated ancestral breed predicted using the 50 K SNP panel and Admixture [66], and animal age at slaughter. The GWAS analyses were performed using a single SNP-based mixed linear model association (MLMA) as implemented in GCTA software [67, 68], and the linear mixed model can be described as follows:

$$ {y}_{ij}=\mu +{b}_j{x}_{ij}+{a}_{ij}+{e}_{ij} $$

where *y*_*ij*_ is the adjusted phenotypic value of the ith animal with the jth SNP (i.e. the ijth animal, bj is the allele substitution effect of SNPj, x_ij_ is the jth SNP genotype of animal i, and it was coded as 0, 1, 2 for genotypes *A*_1_*A*_1_, *A*_1_*A*_2_ and *A*_2_*A*_2_, respectively, a_ij_ is the additive polygenic effect of the *ij*th animal $\sim N\left(0,\boldsymbol{G}{\sigma}_a^2\right)$, and e_ij_ is the random residual effect $\sim N\left(0,\boldsymbol{I}{\sigma}_e^2\right)$. The genomic relationship matrix **G** (GRM) was constructed using GCTA-GRM as implemented in GCTA software and defined in Yang et al. [67, 69], which is essentially the same as the **G** matrix calculated by the second method of VanRaden [70]:

$$ {A}_{jk}=\frac{1}{M}{\sum}_{i=1}^M\frac{\left({x}_{ij}-2{p}_i\right)\left({x}_{ik}-2{p}_i\right)}{2{p}_i\left(1-{p}_i\right)} $$

Where *A*_*jk*_ is off-diagonal element for animal *j* and animal *k* or represents the diagonal element if j = k, with genotype codes of *x*_ij_ = 0, 1, 2 for *A*_1_*A*_1_, *A*_1_*A*_2_, and *A*_2_*A*_2_, respectively. *p*_*j*_ is the allele frequency of *A*_2_ at locus *j* calculated based on SNP genotype data of the population and M is the number of SNPs in the panel. The **G** matrix was constructed using all DNA variants in the 7.8 M DNA variant panel, i.e. mixed linear model with candidate marker included (MLMi) so that the **G** matrix was constructed based on all 30,155 SNPs for the 50 K SNP GWAS and on all the 7,853,211 DNA variants for the 7.8 M SNP panel GWAS.

For each SNP or DNA variant, the allele substitution effect and its *P*-value were estimated using the GCTA package [67, 68]. The phenotypic variance explained by a single SNP was calculated by $\mathrm{Var}\ \left(\%\right)=\frac{2 pq{\beta}^2}{S^2}\ast 100\%$, where *p* and *q* denote the minor frequency and major frequency for the SNP, respectively, ß is the SNP allele substitution effect, and 2*pqβ*^2^ is the additive genetic variance, and *S*^2^ is phenotypic variance. DNA variants (or SNPs) that have a nominal *P*-value < 0.005 were considered as suggestive QTLs as proposed by Benjamin et al. [26], while SNPs with a nominal *P*-value < 10^− 5^ were classified as significant QTLs based on the recommendation of the Wellcome Trust Case Control Consortium [71]. SNPs that have a nominal *P*-value < 10^− 5^ were further examined for the genome-wise false discovery rate (FDR), which was calculated following the Benjamini-Hochberg procedure for each SNP [27]. At each significance threshold when multiple SNPs within a window of 70 kb upstream and downstream are significantly associated with a trait, the SNP with the lowest nominal *P*-value was identified as the lead SNP whereas the remaining SNPs were classified as support SNPs. A 70 kb window was chosen for this study as this was the chromosomal length within which a high LD phase correlation (> 0.77) was maintained in a Canadian multibreed population [54].

Heritability of a trait was estimated using GREML-LDMS [72, 73] for both the 50 K SNP panel and the 7.8 M DNA variant panel. In GREML-LDMS, DNA variants were stratified into four groups by their mean LD scores within a sliding window, representing the first, second, third, and fourth quartiles of the mean LD score distribution. A GRM was subsequently constructed with DNA variants in each group. The GRMs were then fitted simultaneously into the above statistical model without the single DNA variant effect and the variance components were estimated via a restricted maximum likelihood (REML) as implemented in the GCTA package [67, 69, 74, 75]. The genomic heritability of a trait was calculated as a ratio of the total additive genetic variance over the phenotypic variance of the trait.

### Inference of genetic architecture based on GWAS results

Distribution of SNP effects of each carcass trait was generated by plotting squared allele substitution effects of all DNA variants in the 7.8 M DNA variant panel, and by plotting the amount of additive genetic variances explained by single DNA variants in the panel. The average of squared allele substitution effects was obtained for each of the 9 broad functional classes (Table 3) by summing all squared allele substitution effects within the broad functional class divided by the total number of DNA variants within the functional class. The additive genetic variance accounted for by each of the 9 functional classes was estimated by fitting the GRM constructed based on the DNA variants of the functional class and the GRM constructed based on the DNA variants of all other functional classes simultaneously in the statistical model using the GCTA package. The amount of additive genetic variance explained per sequence variant within a functional class was obtained by the additive genetic variance captured by the functional class divided by the number of DNA variants in the class.

### Candidate gene identification and functional enrichment analyses

Lead SNPs with a FDR < 0.10 were selected to search for candidate genes. Subsequently, genes located within 70 kb upstream and downstream of the lead SNP were considered candidate genes associated with the trait based on SNP annotation information from the UMD3.1 bovine genome assembly from the Ensembl genome browser (https://www.ensembl.org/). Ingenuity Pathway Analysis (IPA) (Ingenuity® Systems, Redwood City, CA; https://www.qiagenbioinformatics.com/products/ingenuity-pathway-analysis/) (IPA Spring 2019 release) was used for the functional enrichment analyses of the candidate genes identified via the GWAS. Briefly, for the genes with known human orthologues from Ensembl, their gene IDs were replaced with their human orthologous gene IDs, whereas those without human orthologues their bovine gene IDs were maintained in the gene list. These Ensembl gene IDs were then used as input gene identifiers in IPA and a core analysis was performed on the genes that were mapped to the IPA knowledge base database. With the list of candidate genes and genes mapped to the human orthologues, enhanced molecular processes and gene network were inferred using IPA. Molecular, cellular, and biological processes or functions were significantly enriched if the *P*-value for the overlap comparison test between the input gene list and the IPA knowledge base database for a given biological function was less than 0.05. Additionally, genes and biological processes or sub-functions’ interaction networks within the most significant molecular and cellular function were produced to show possible biological networks for the trait.

## Supplementary information

**Additional file 1: **This file contains Additional figures including **Figure S1.** Distribution of SNP allele substitution effects (left) and additive genetic variances explained by individual SNP (right) based on GWAS of imputed 7.8 M whole genome sequence (WGS) variants for HCW, AFAT, REA, LMY, and AFAT; **Figure S2.** Cellular and molecular processes for HCW, AFAT, REA, LMY, and CMAR; **Figure S3.** Gene network for major gene expression, Carbohydrate/lipid metabolisms and Cell Morphology for HCW, AFAT, REA, LMY, and CMAR; **Figure S4.** Venn diagram showing the overlapped lead significant SNPs (a) and candidate genes (b) among five carcass merit traits based on the imputed 7.8 M DNA variant GWAS.

**Additional file 2: **This file contains information of all suggestive significant SNPs/INDELs at *P*-values < 0.005 based on the 7.8 M WGS variant GWAS for HCW, AFAT, REA, LMY, and CMAR.

**Additional file 3: **This file contains additional Tables including **Table S1.** Functional annotation of 7.8 M WGS variants along with the number of variants in each class, classification of SNP functions, percentage of WGS and 9 functional class assignments; **Table S2.** Functional annotation of 50 K SNP genotypes after quality control along with the number of variants in each class, classification of SNP functions, percentage of WGS and 9 functional class assignments; **Table S3.** SNP functional annotation of all DNA variants (38,318,974) based on DNA variants of the 1000 bulls genome project; **Table S4.** List of lead SNPs that were overlapped with QTLs published in the Cattle QTL database within 1 M bp up or 1 M bp downstream for HCW, AFAT, REA, LMY, and CMAR.

## Data Availability

The datasets supporting the results of this article are included within the article and its additional files. The original genotype and phenotype data sets are available for non-commercial purposes following the execution of a materials transfer agreement. Whole genome sequence data collected for the cattle populations in this study and used in imputation is available from the NCBI SRA database under BioProjects PRJNA176557 and PRJNA256210.

## Abbreviations

ADG: Average daily gain
AFAT: Average backfat thickness
BTA: *Bos taurus* autosome
CMAR: Carcass marbling score
DMI: Dry matter intake
DNA: Deoxyribonucleic acid
FDR: Genome-wide false discovery rate
GRM: Genomic relationship matrix
GWAS: Genome-wide association study
HCW: Hot carcass weight
HWT: Hardy-Weinberg equilibrium test
INDEL: Insertion and deletion
LD: Linkage disequilibrium
LMY: Lean meat yield
MAF: Minor allele frequency
QTL: Quantitative trait loci
REA: Rib eye area
SNP: Single nucleotide polymorphism
WGS: Whole genome sequence
