# Supplementary material for: Genetic architecture of quantitative traits in beef cattle revealed by genome wide association studies of imputed whole genome sequence variants: II: carcass merit traits
Source: BMC Genomics. 2020 Jan 13;21:38. doi: 10.1186/s12864-019-6273-1 (PMC6958779; doi:10.1186/s12864-019-6273-1)
Supplement: Supplementary file 1 — Additional file 1: This file contains Additional figures including Figure S1. Distribution of SNP allele substitution effects (left) and additive genetic variances explained by individual SNP (right) based on GWAS of imputed 7.8 M whole genome sequence (WGS) variants for HCW, AFAT, REA, LMY, and AFAT; Figure S2. Cellular and molecular processes for HCW, AFAT, REA, LMY, and CMAR; Figure S3. Gene network for major gene expression, Carbohydrate/lipid metabolisms and Cell Morphology for HCW, AFAT, REA, LMY, and CMAR; Figure S4. Venn diagram showing the overlapped lead significant SNPs (a) and candidate genes (b) among five carcass merit traits based on the imputed 7.8 M DNA variant GWAS. [file 12864_2019_6273_MOESM1_ESM.pdf]

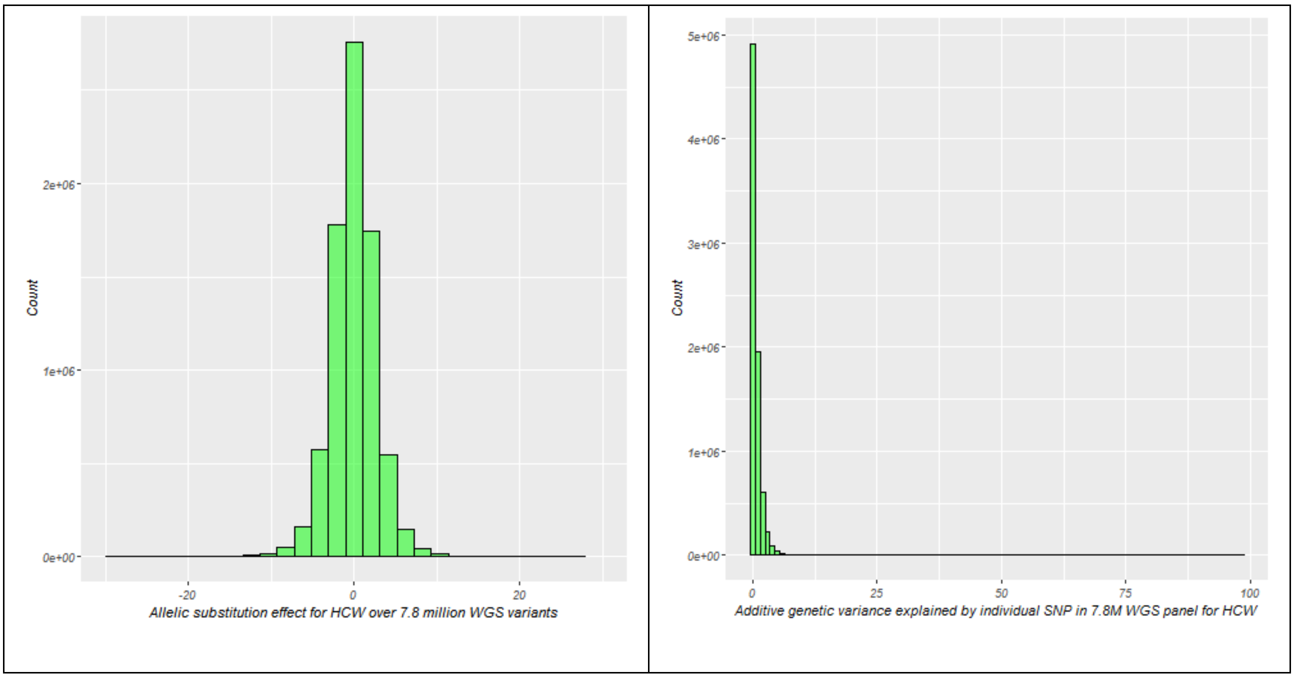


HCW_7.8M GWAS

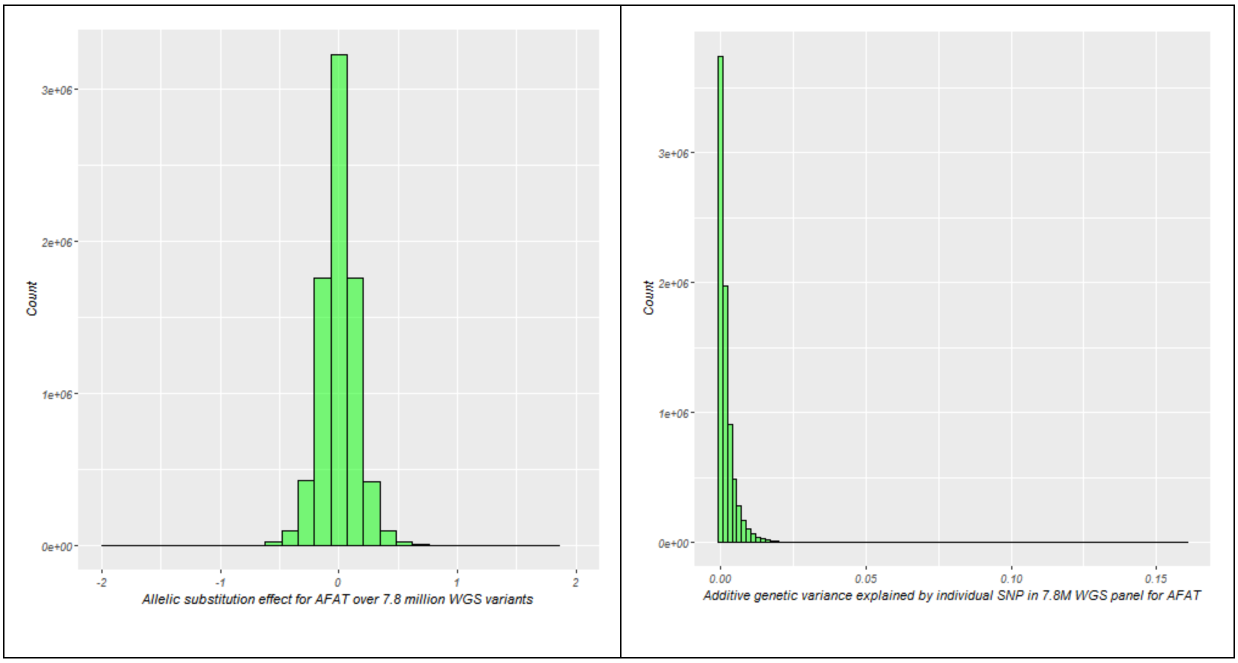


AFAT_7.8M GWAS

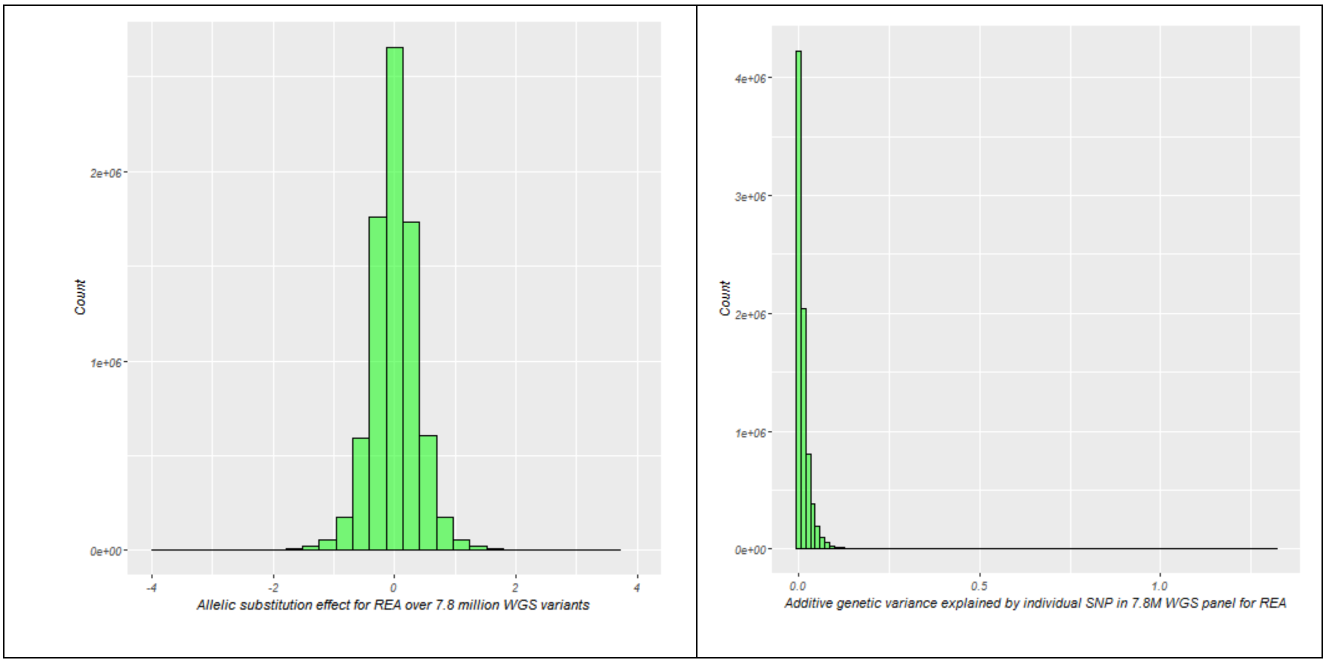


REA_7.8M GWAS

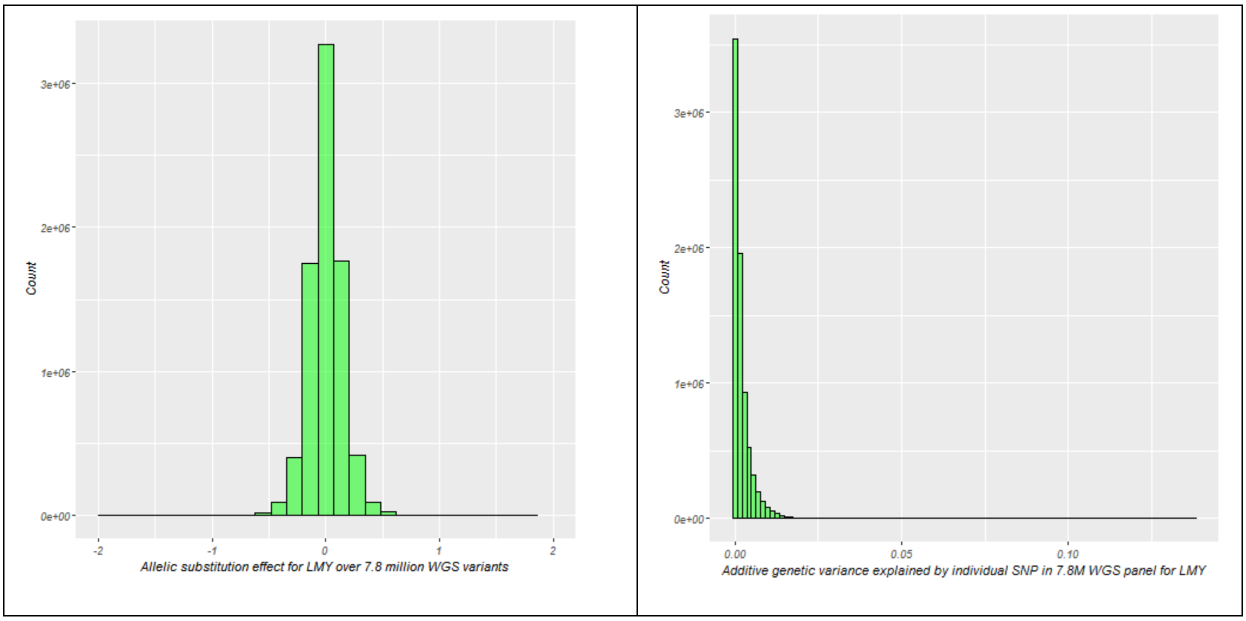


LMY_7.8M GWAS

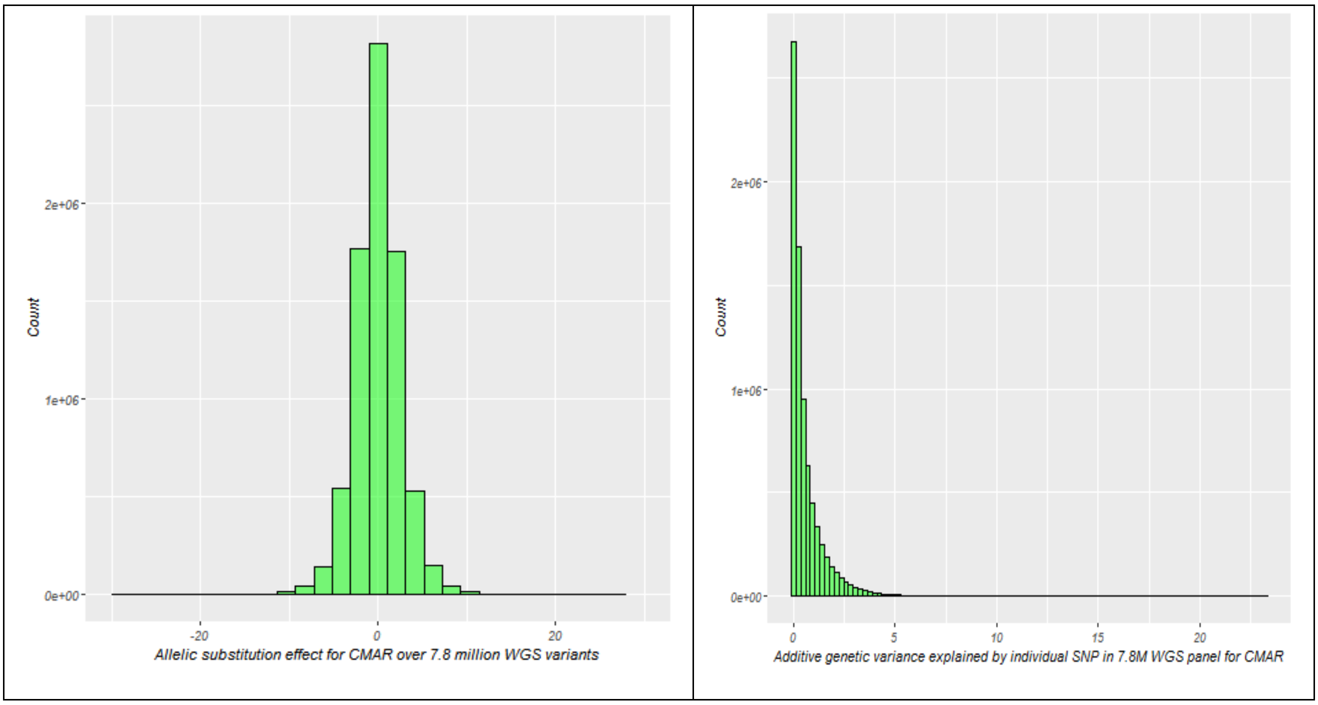


CMAR_7.8M GWAS

**Figure S1.** Distribution of SNP allele substitution effects (left) and additive genetic variances explained by individual SNPs (right) based on GWAS of imputed 7.8M whole genome sequence (WGS) variants for HCW, AFAT, REA, LMY, and CMAR.

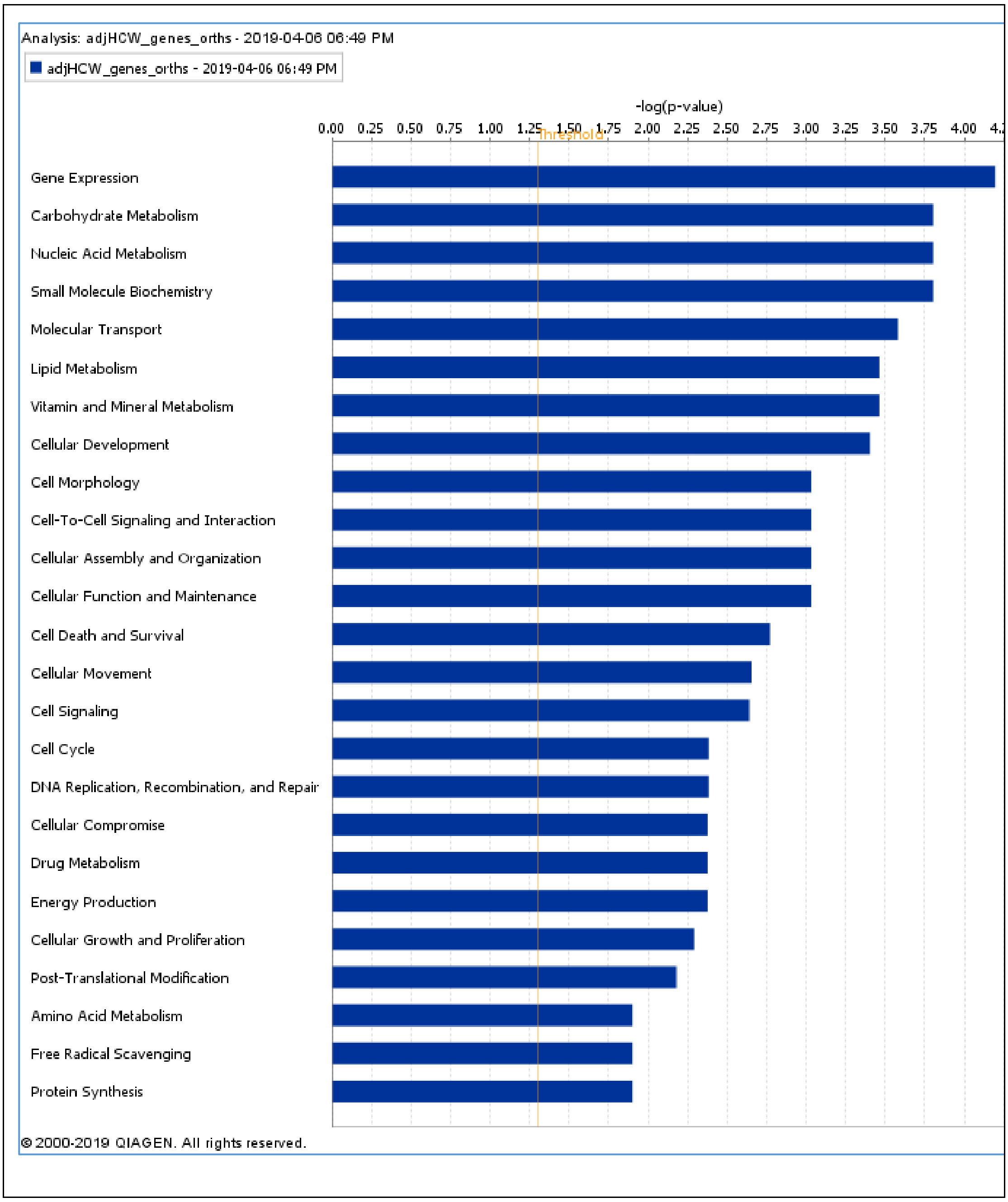

Analysis: adjHCW_genes_orths - 2019-04-06 06:49 PM
adjHCW_genes_orths - 2019-04-06 06:49 PM
-log(p-value)
0.00
0.25
0.50
0.75
1.00
1.25
1.50
1.75
2.00
2.25
2.50
2.75
3.00
3.25
3.50
3.75
4.00
Threshold
Gene Expression
Carbohydrate Metabolism
Nucleic Acid Metabolism
Small Molecule Biochemistry
Molecular Transport
Lipid Metabolism
Vitamin and Mineral Metabolism
Cellular Development
Cell Morphology
Cell-To-Cell Signaling and Interaction
Cellular Assembly and Organization
Cellular Function and Maintenance
Cell Death and Survival
Cellular Movement
Cell Signaling
Cell Cycle
DNA Replication, Recombination, and Repair
Cellular Compromise
Drug Metabolism
Energy Production
Cellular Growth and Proliferation
Post-Translational Modification
Amino Acid Metabolism
Free Radical Scavenging
Protein Synthesis
© 2000-2019 QIAGEN. All rights reserved.

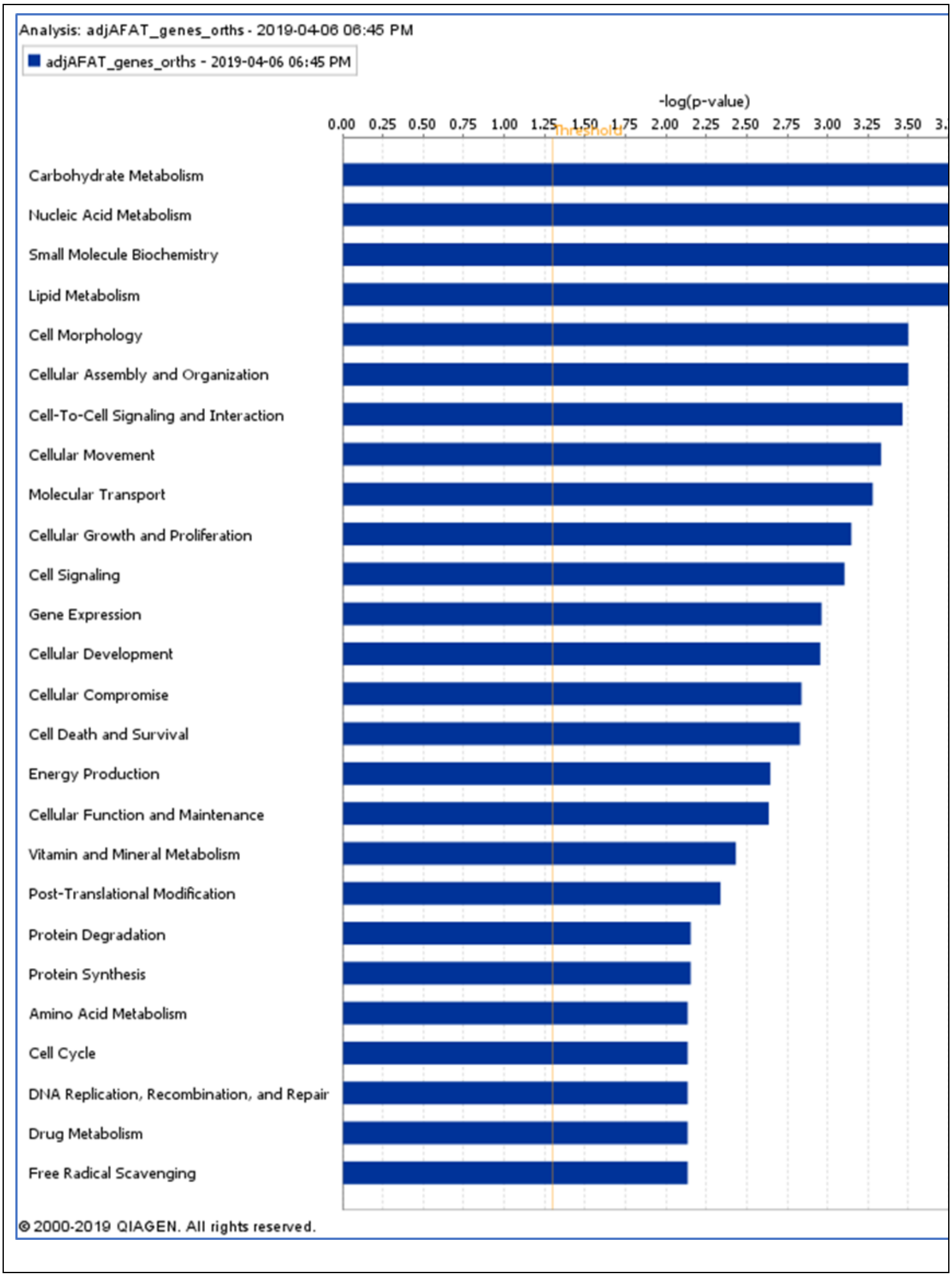
Analysis: adjAFAT_genes_orths - 2019-04-06 06:45 PM
adjAFAT_genes_orths - 2019-04-06 06:45 PM
-log(p-value)
0.00
0.25
0.50
0.75
1.00
1.25
1.50
1.75
2.00
2.25
2.50
2.75
3.00
3.25
3.50
Threshold
Carbohydrate Metabolism
Nucleic Acid Metabolism
Small Molecule Biochemistry
Lipid Metabolism
Cell Morphology
Cellular Assembly and Organization
Cell-To-Cell Signaling and Interaction
Cellular Movement
Molecular Transport
Cellular Growth and Proliferation
Cell Signaling
Gene Expression
Cellular Development
Cellular Compromise
Cell Death and Survival
Energy Production
Cellular Function and Maintenance
Vitamin and Mineral Metabolism
Post-Translational Modification
Protein Degradation
Protein Synthesis
Amino Acid Metabolism
Cell Cycle
DNA Replication, Recombination, and Repair
Drug Metabolism
Free Radical Scavenging
© 2000-2019 QIAGEN. All rights reserved.

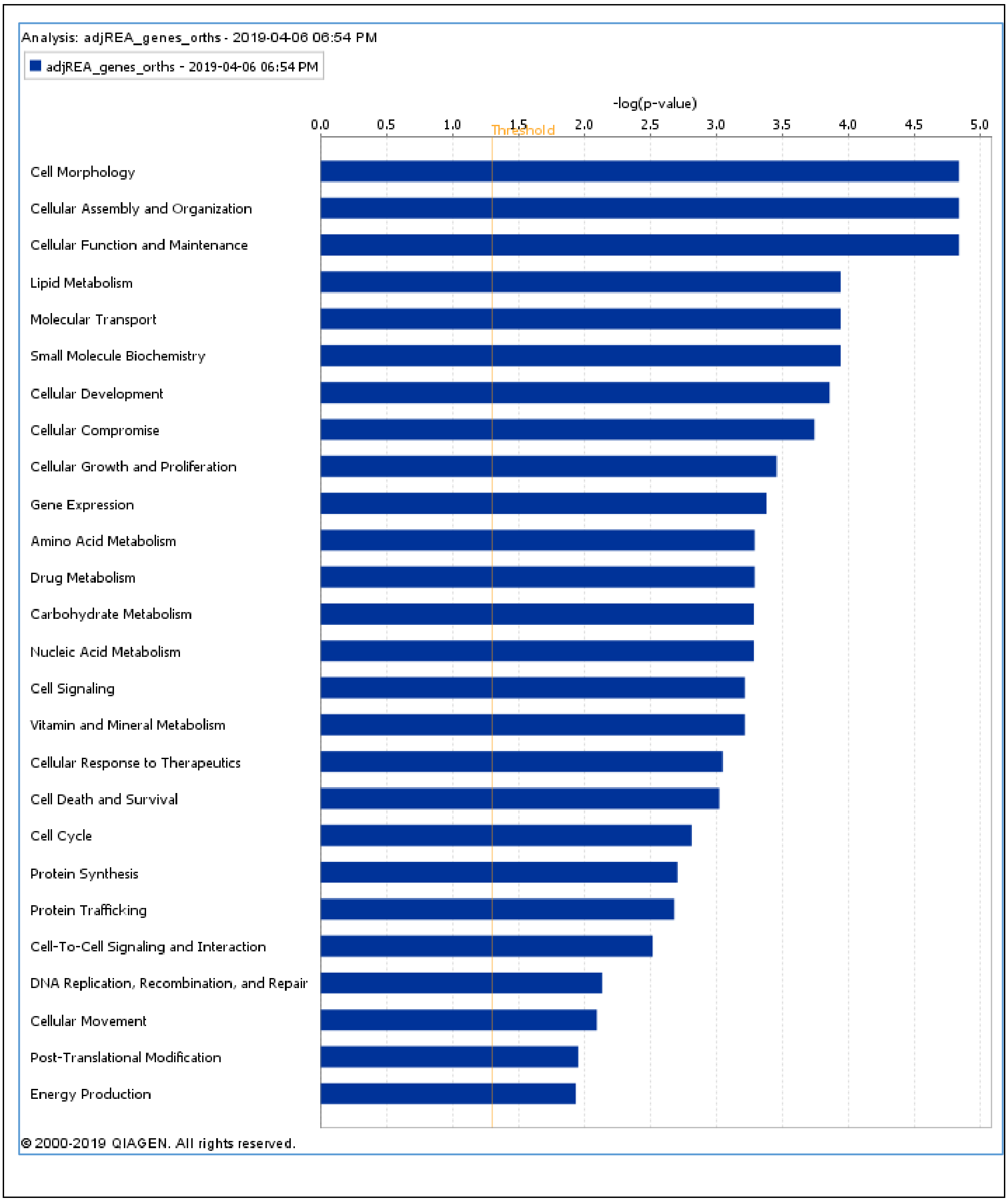

Analysis: adjREA_genes_orths - 2019-04-06 06:54 PM
adjREA_genes_orths - 2019-04-06 06:54 PM
-log(p-value)
0.0
0.5
1.0
1.5
2.0
2.5
3.0
3.5
4.0
4.5
5.0
Threshold
Cell Morphology
Cellular Assembly and Organization
Cellular Function and Maintenance
Lipid Metabolism
Molecular Transport
Small Molecule Biochemistry
Cellular Development
Cellular Compromise
Cellular Growth and Proliferation
Gene Expression
Amino Acid Metabolism
Drug Metabolism
Carbohydrate Metabolism
Nucleic Acid Metabolism
Cell Signaling
Vitamin and Mineral Metabolism
Cellular Response to Therapeutics
Cell Death and Survival
Cell Cycle
Protein Synthesis
Protein Trafficking
Cell-To-Cell Signaling and Interaction
DNA Replication, Recombination, and Repair
Cellular Movement
Post-Translational Modification
Energy Production
© 2000-2019 QIAGEN. All rights reserved.

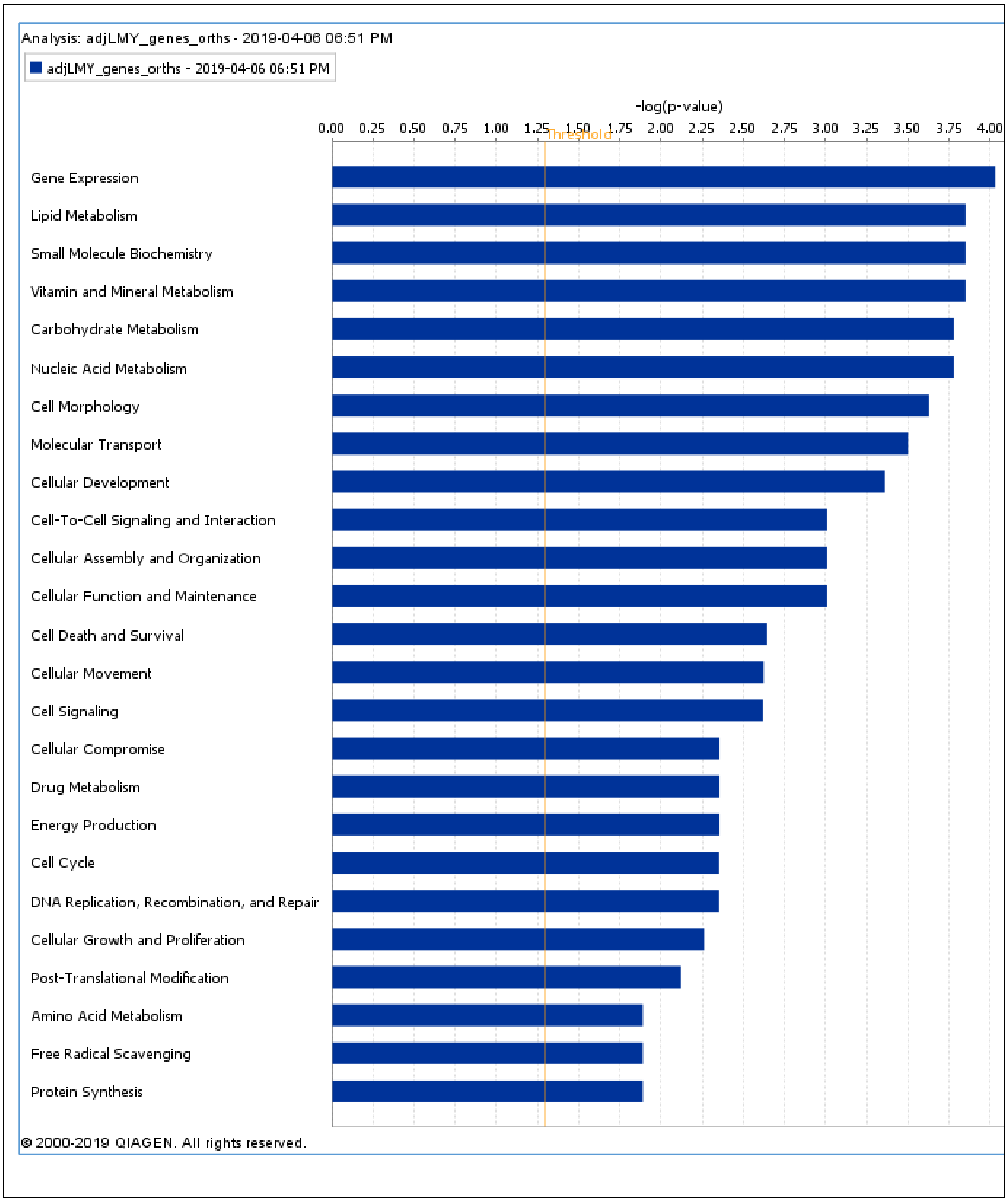

Analysis: adjLMY_genes_orths - 2019-04-06 06:51 PM
adjLMY_genes_orths - 2019-04-06 06:51 PM
-log(p-value)
0.00
0.25
0.50
0.75
1.00
1.25
1.50
1.75
2.00
2.25
2.50
2.75
3.00
3.25
3.50
3.75
4.00
Threshold
Gene Expression
Lipid Metabolism
Small Molecule Biochemistry
Vitamin and Mineral Metabolism
Carbohydrate Metabolism
Nucleic Acid Metabolism
Cell Morphology
Molecular Transport
Cellular Development
Cell-To-Cell Signaling and Interaction
Cellular Assembly and Organization
Cellular Function and Maintenance
Cell Death and Survival
Cellular Movement
Cell Signaling
Cellular Compromise
Drug Metabolism
Energy Production
Cell Cycle
DNA Replication, Recombination, and Repair
Cellular Growth and Proliferation
Post-Translational Modification
Amino Acid Metabolism
Free Radical Scavenging
Protein Synthesis
© 2000-2019 QIAGEN. All rights reserved.

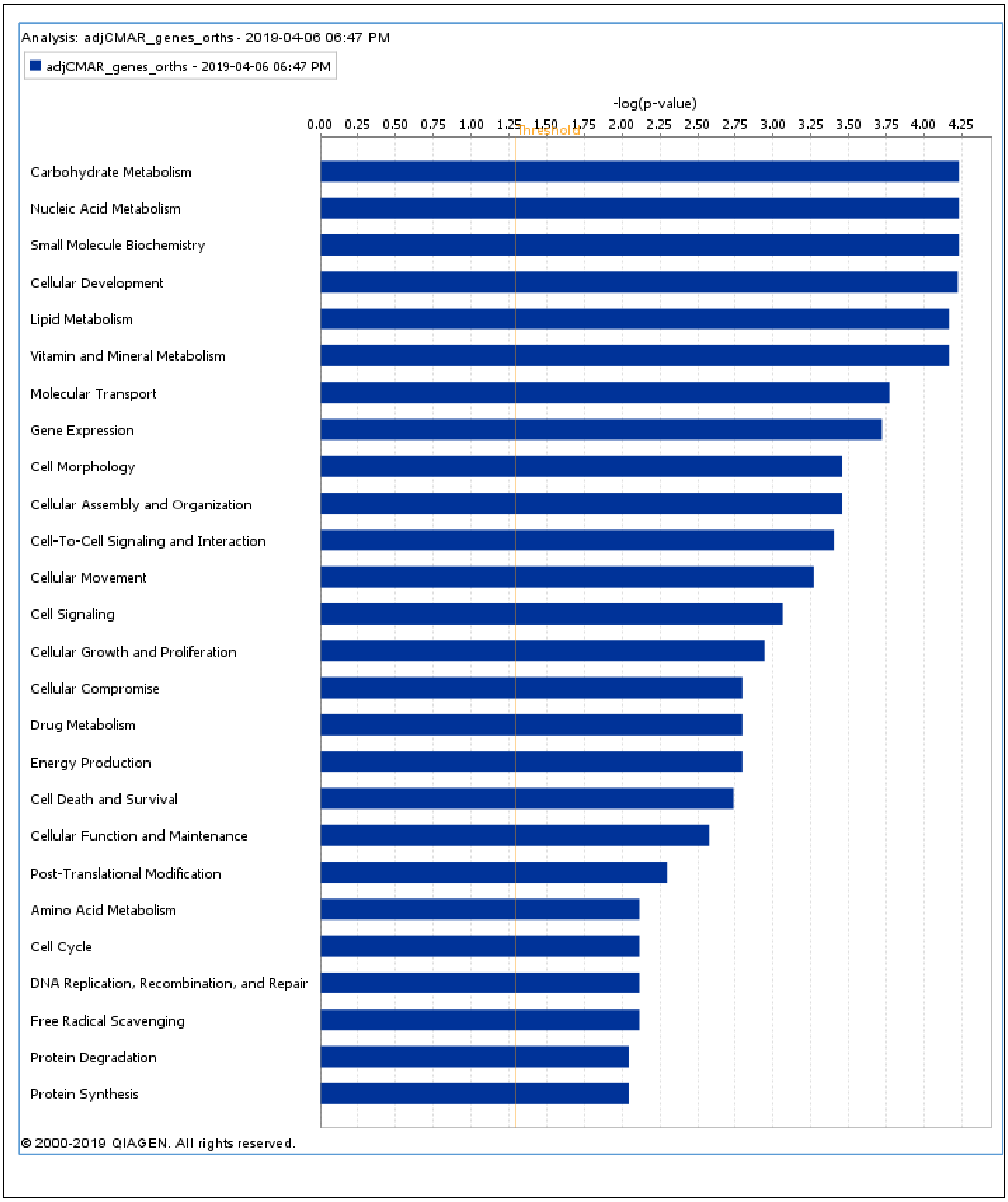


**Figure S2.** Cellular and molecular processes for HCW, AFAT, REA, LMY, and CMAR. The images were generated through the use of Ingenuity Pathway Analysis (IPA) and written permission was granted by QIAGEN Silicon Valley to use and adapt the figure generated by IPA under the terms of the Creative Commons Attribution License (CC BY) 4.0.

HCW GENE EXPRESSION NETWORK

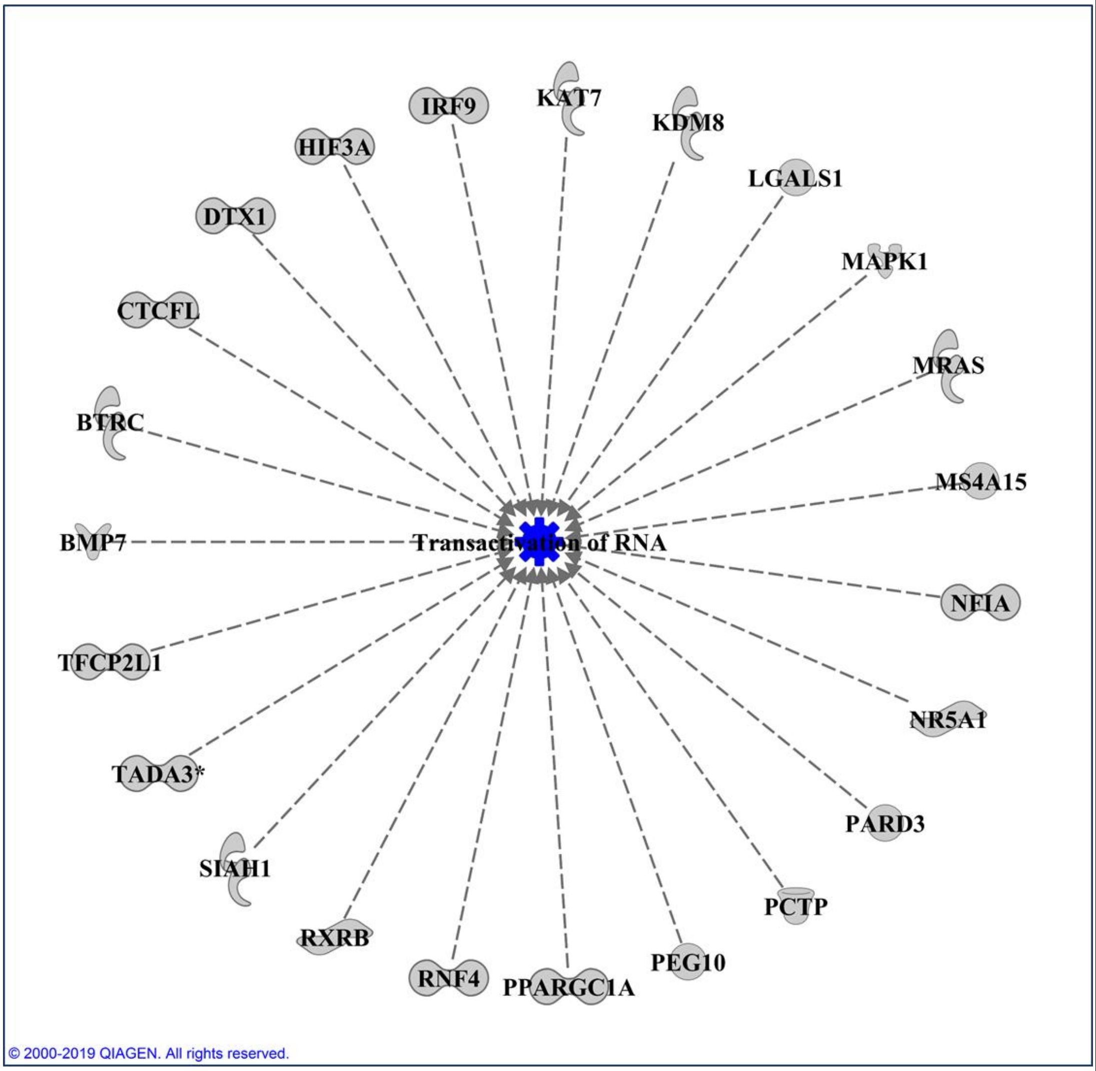


HCW CARBOHYDRATE METABOLISM NETWORK

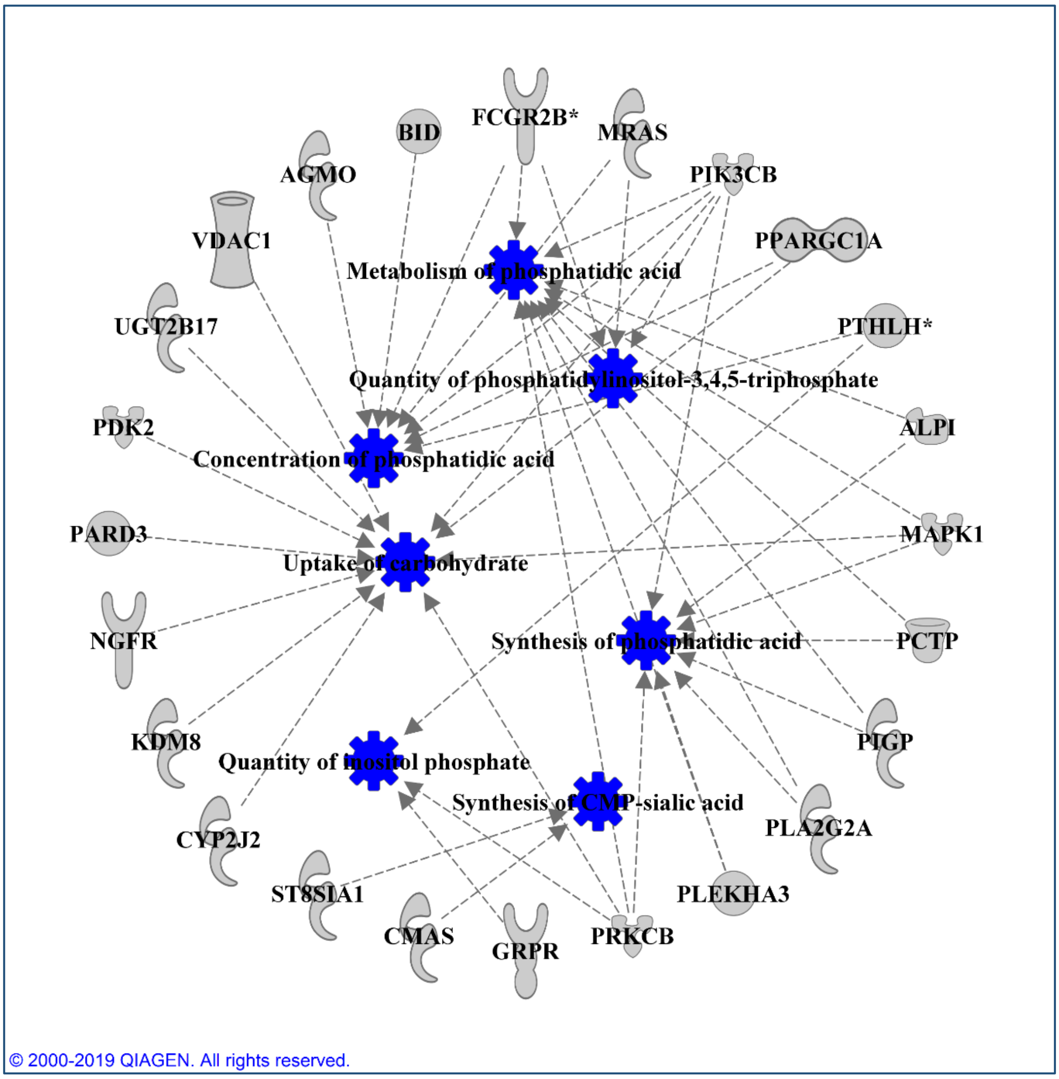


HCW LIPID METABOLISM NETWORK

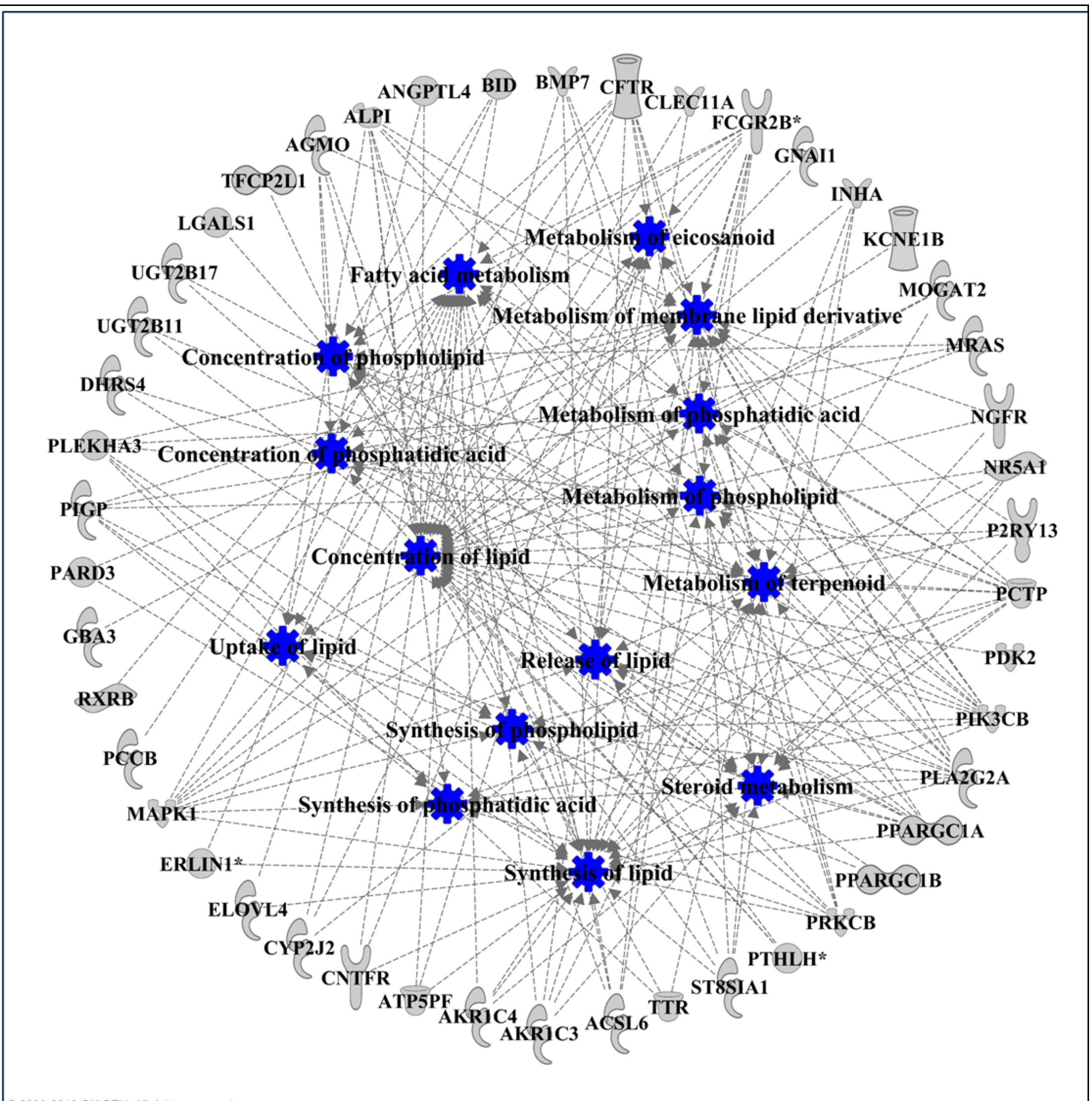

© 2000-2019 QIAGEN. All rights reserved.

AFAT CARBOHYDRATE METABOLISM NETWORK

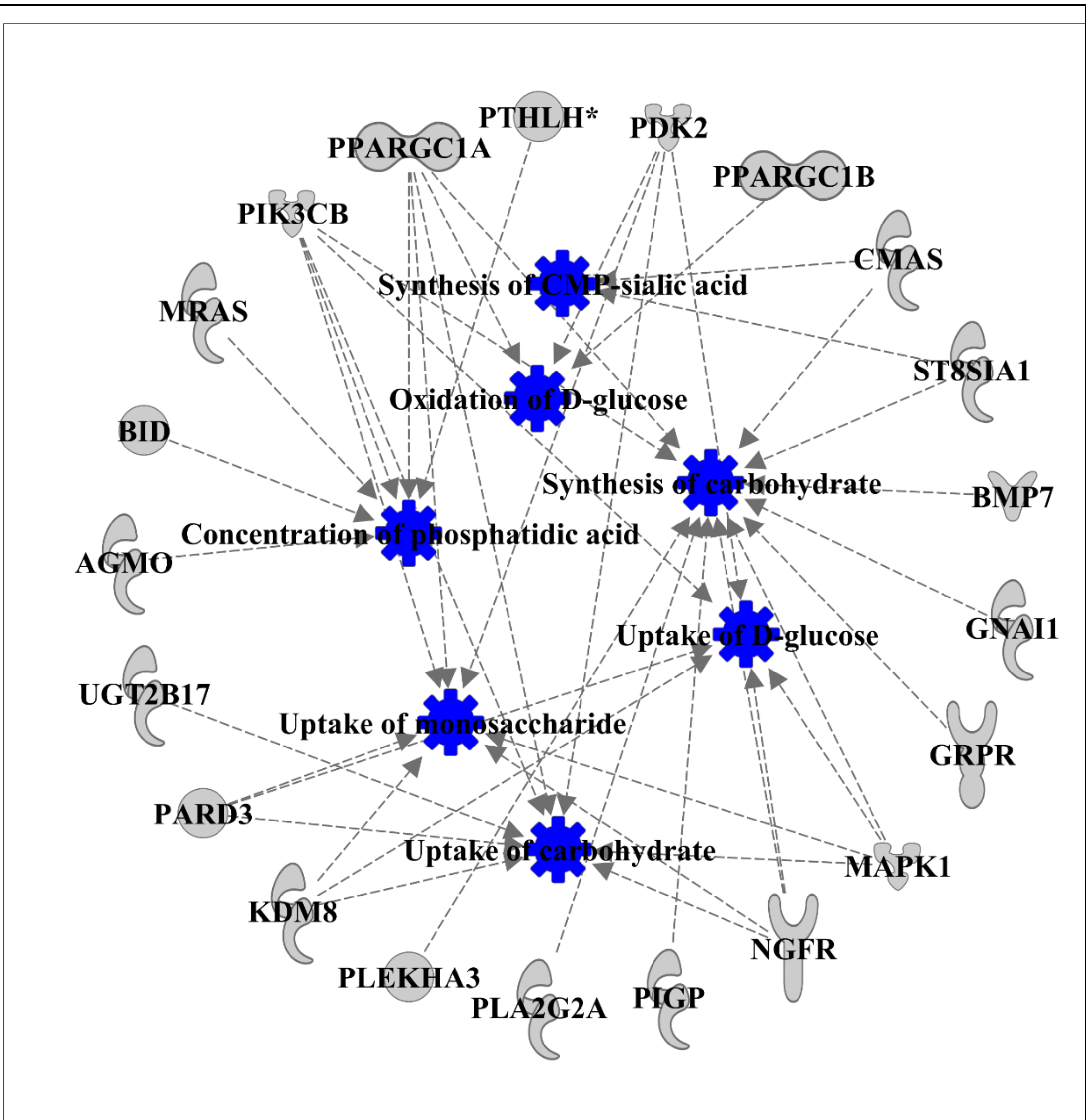

© 2000-2019 QIAGEN. All rights reserved.

AFAT CELL MORPHOLOGY NETWORK

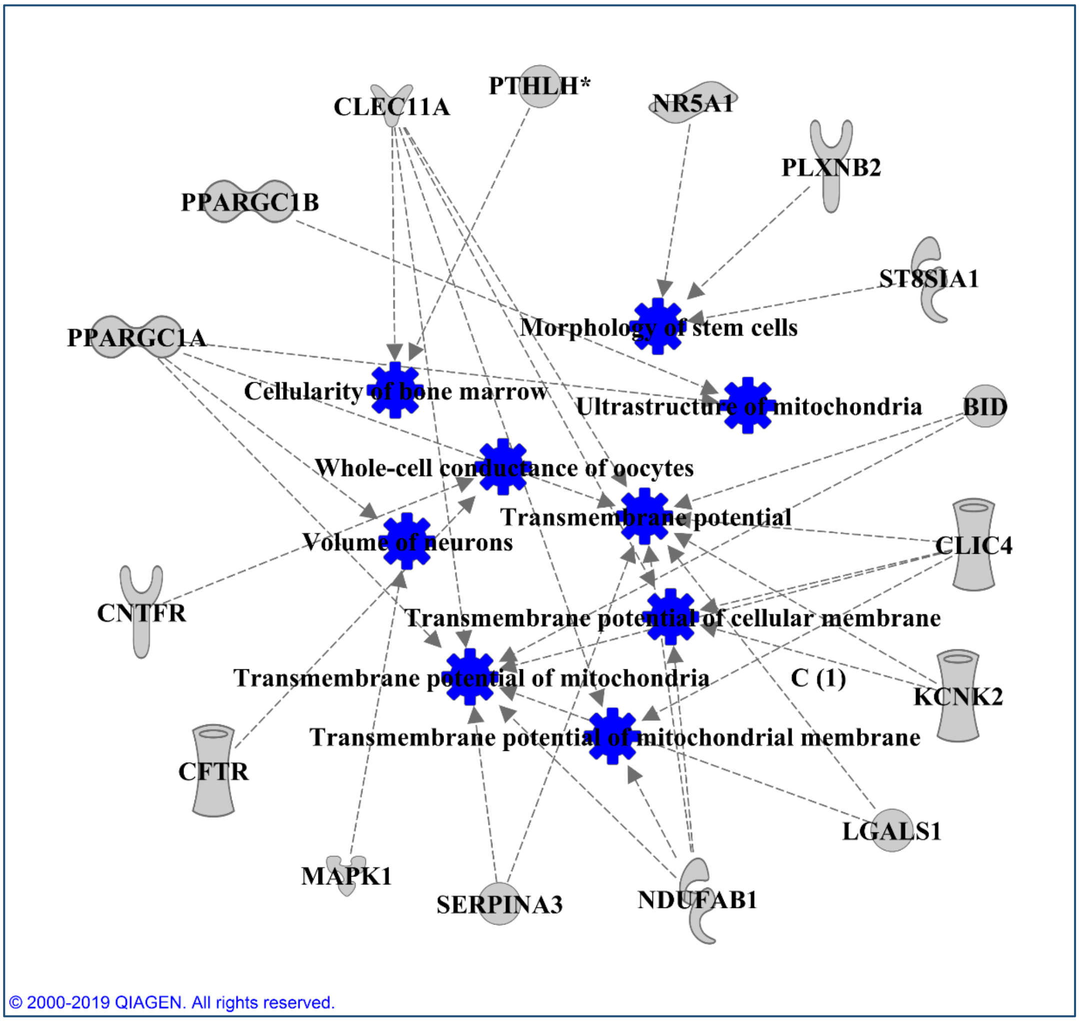


© 2000-2019 QIAGEN. All rights reserved.

AFAT LIPID METABOLISM NETWORK

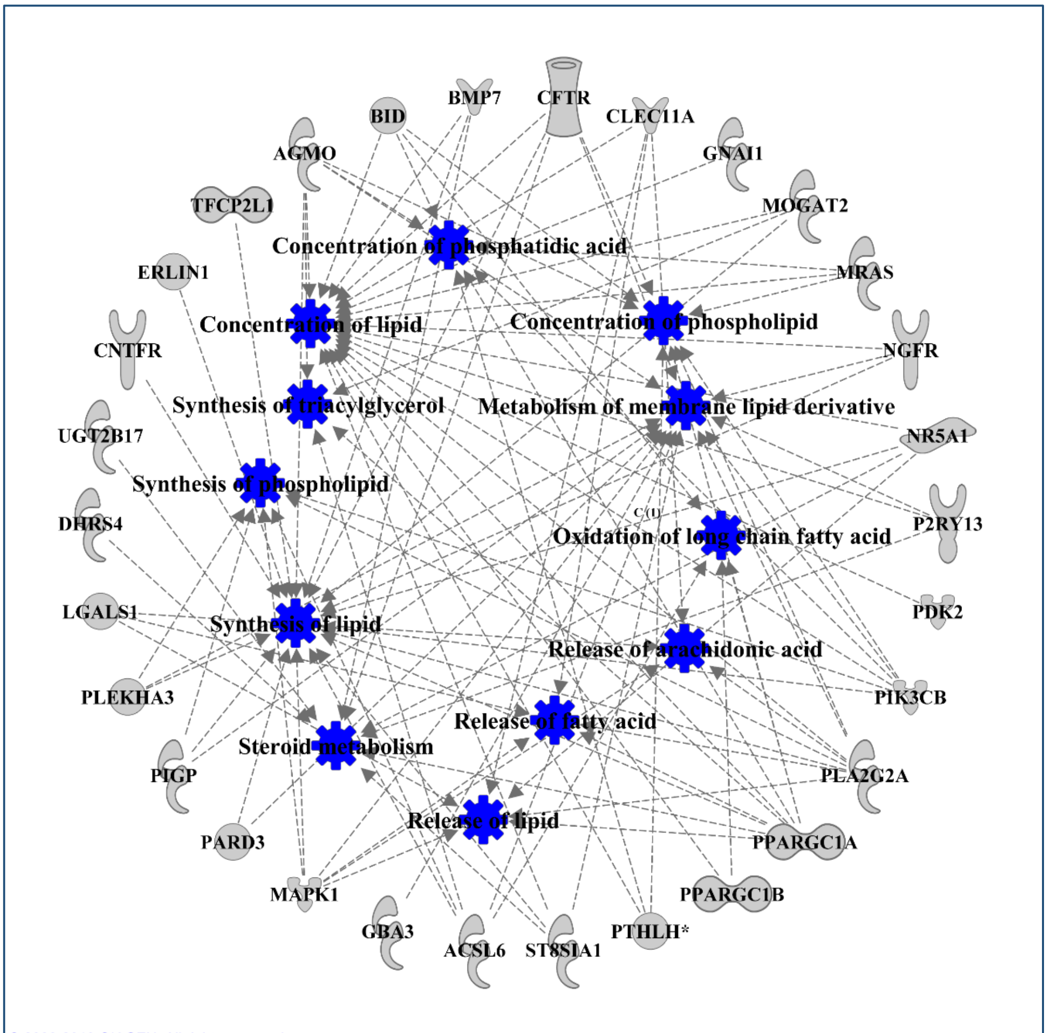

© 2000-2019 QIAGEN. All rights reserved.

REA CELL MORPHOLOGY NETWORK

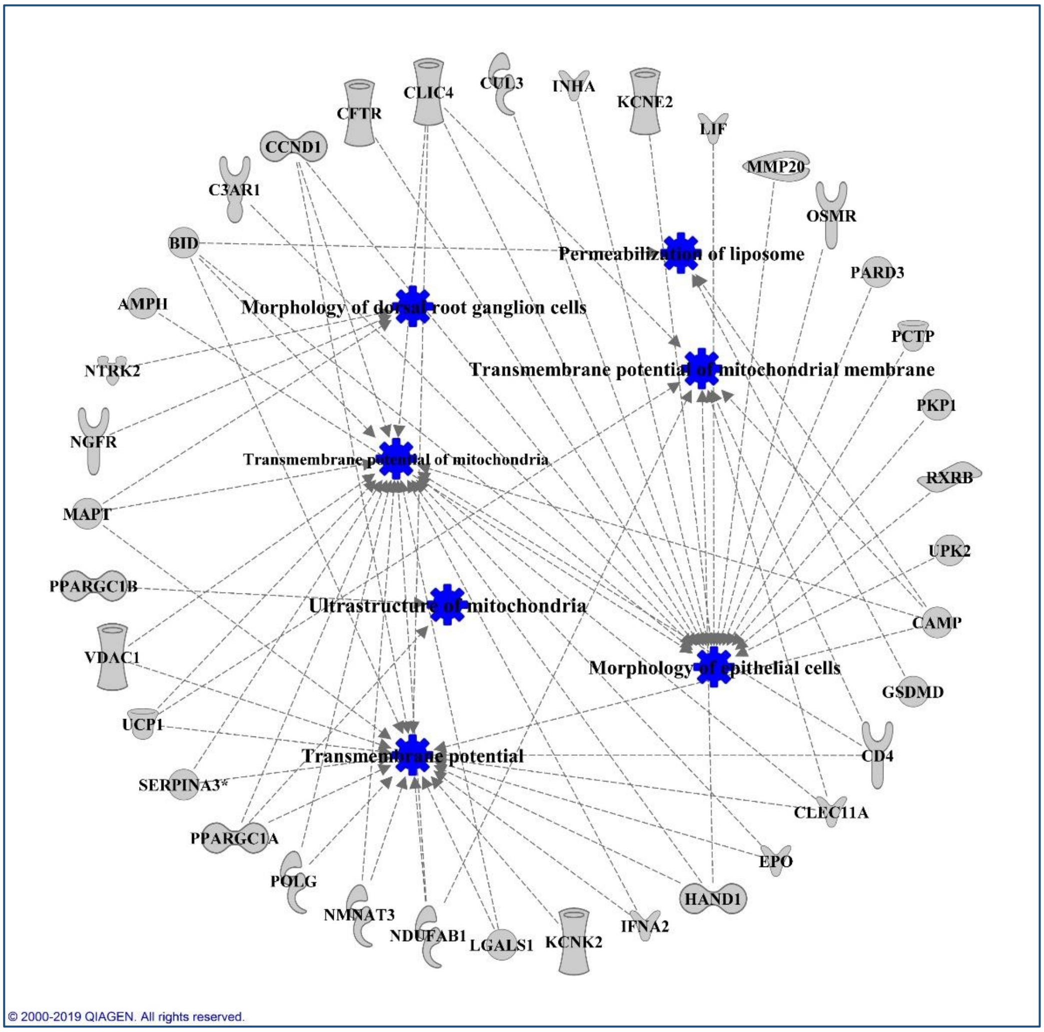

© 2000-2019 QIAGEN. All rights reserved.

REA LIPID METABOLISM NETWORK

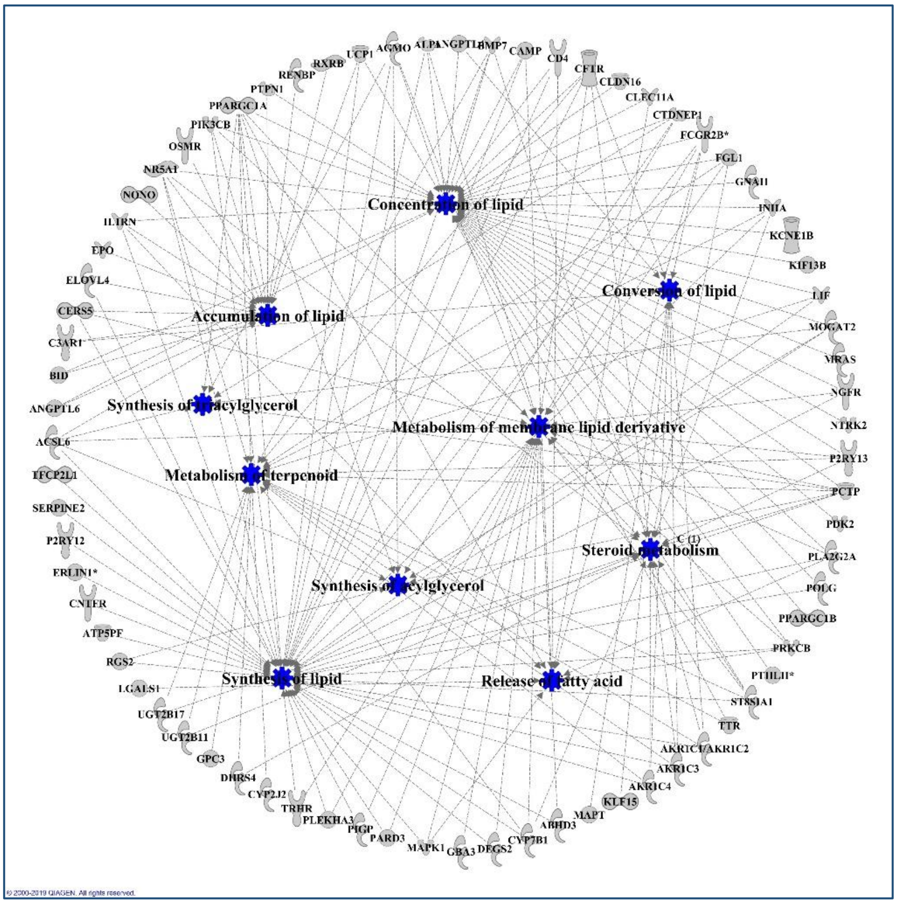


LMY GENE EXPRESSION NETWORK

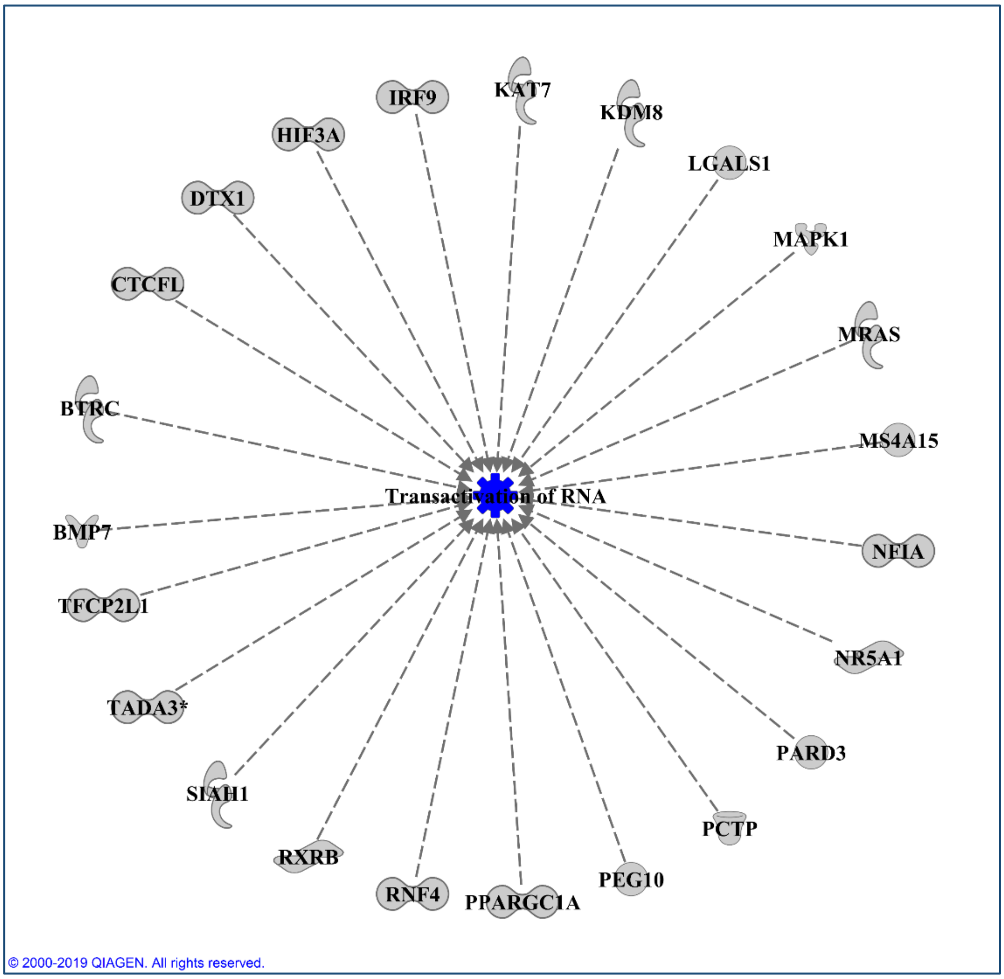

© 2000-2019 QIAGEN. All rights reserved.

LMY LIPID METABOLISM NETWORK

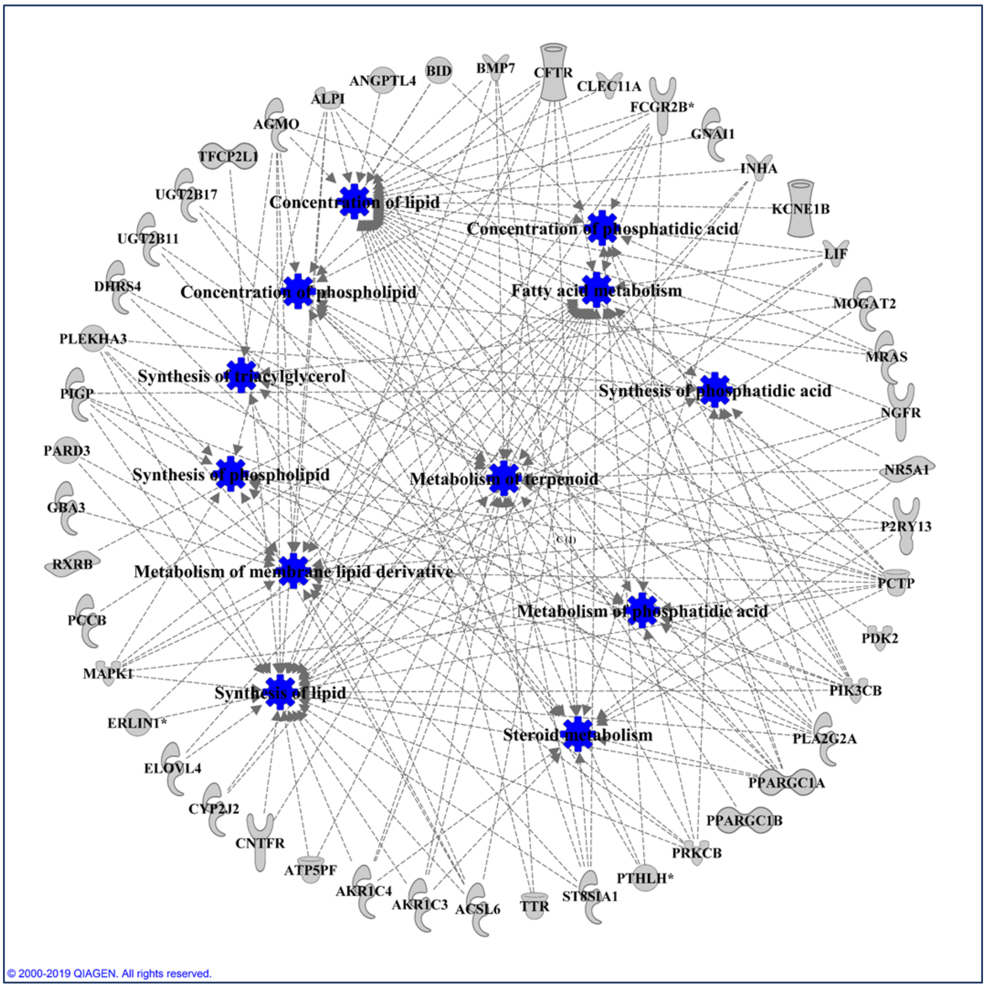

© 2000-2019 QIAGEN. All rights reserved.

LMY CARBOHYDRATE METABOLISM NETWORK

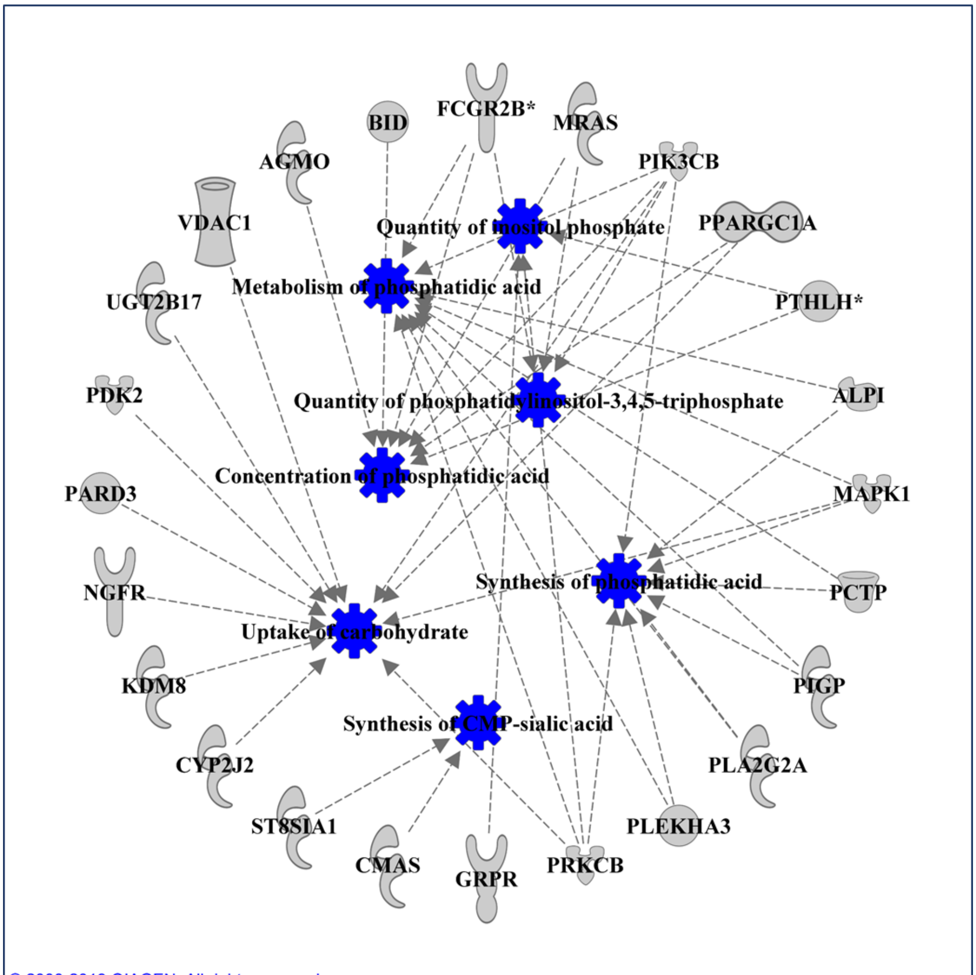

© 2000-2019 QIAGEN. All rights reserved.

CMAR CARBOHYDRATE METABOLISM NETWORK

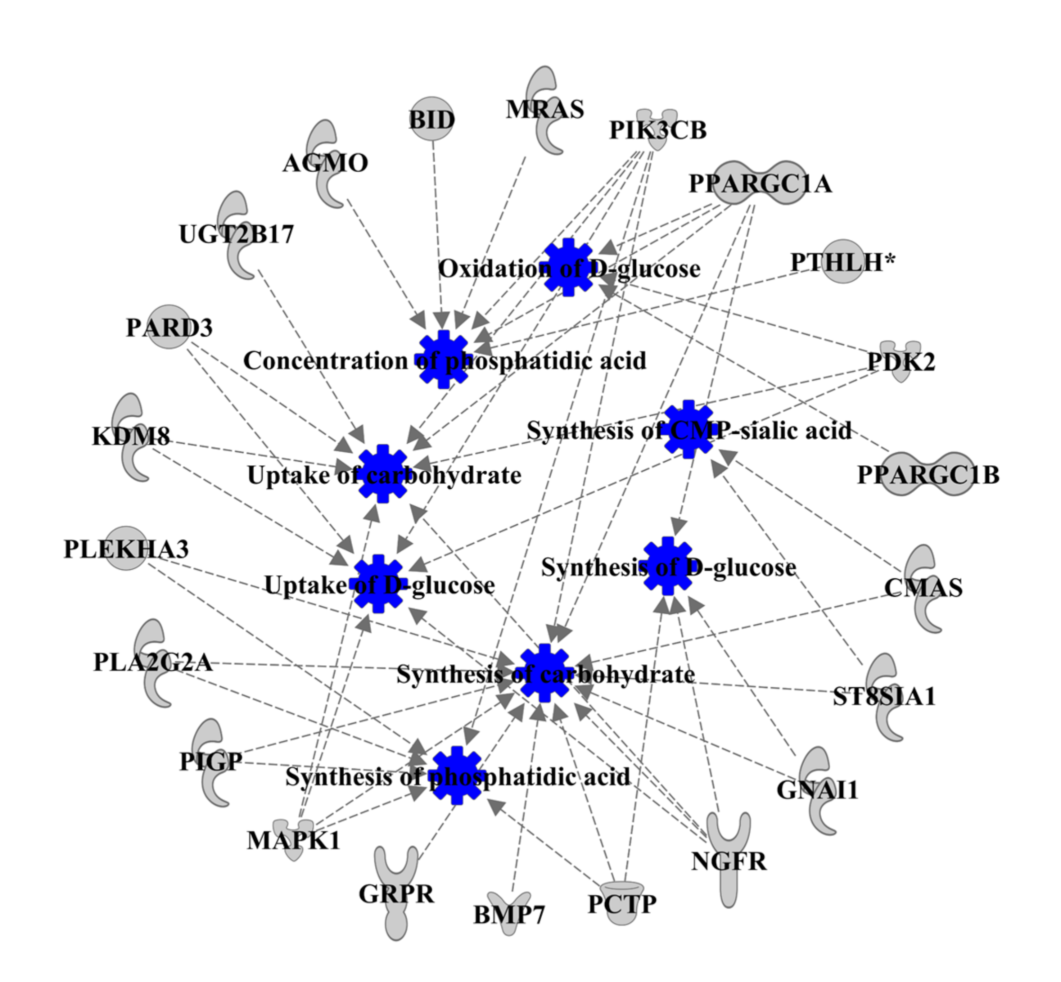


© 2000-2019 QIAGEN. All rights reserved.

CMAR LIPID METABOLISM NETWORK

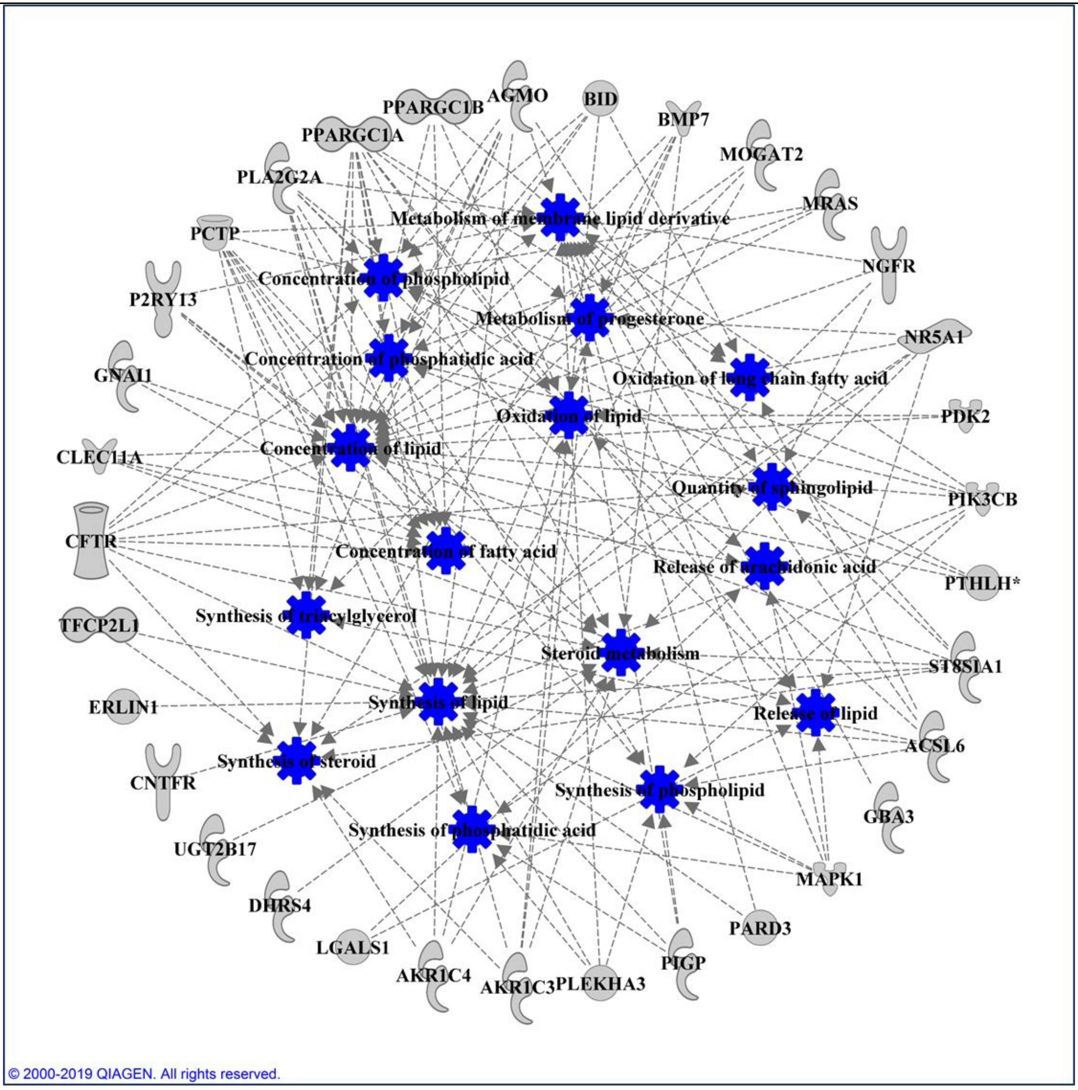

© 2000-2019 QIAGEN. All rights reserved.

CMAR CELLULAR DEVELOPMENT NETWORK

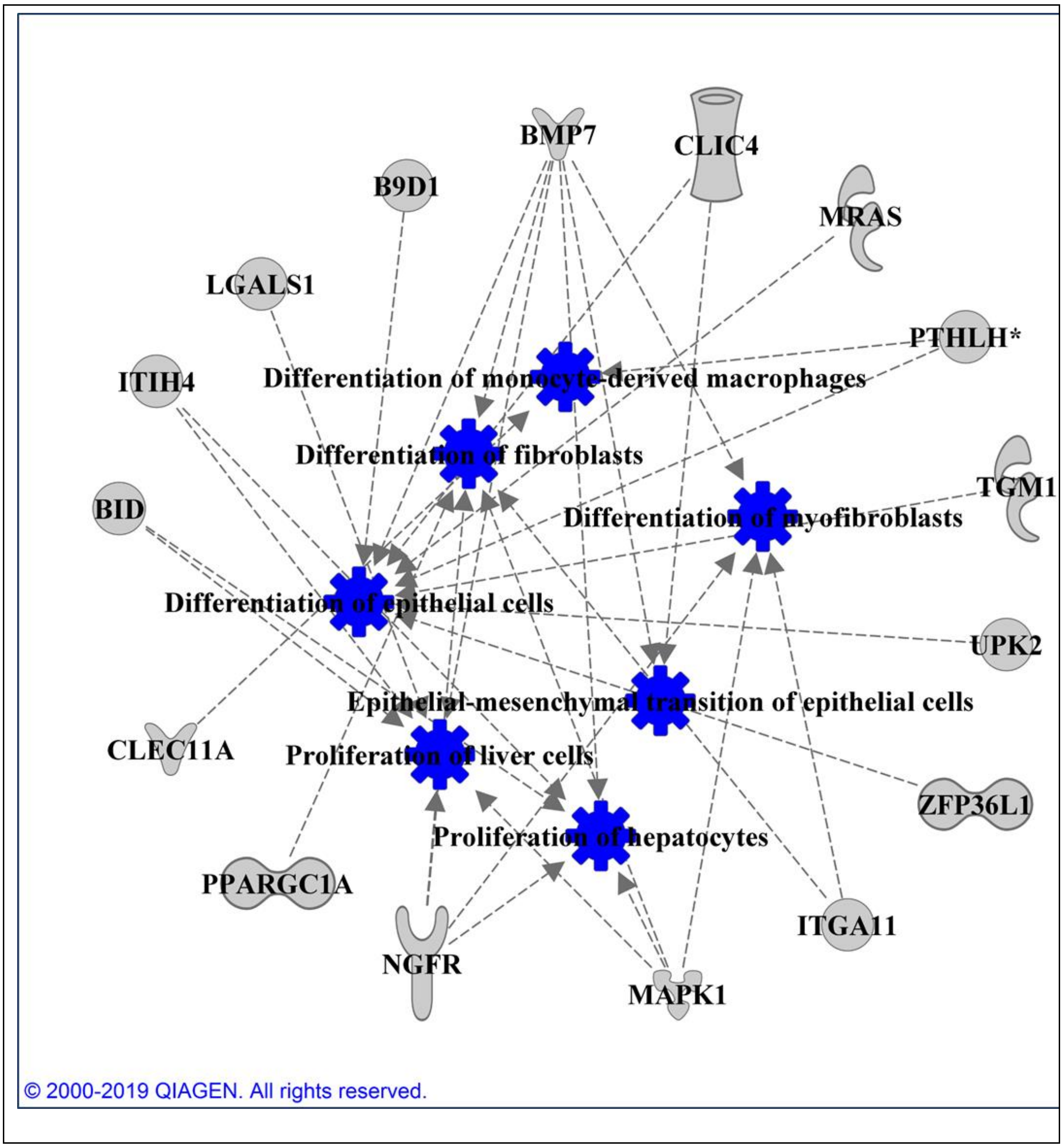


**Figure S3.** Gene network for major gene expression, Carbohydrate/lipid metabolisms and Cell Morphology for HCW, AFAT, REA, LMY, and CMAR. The images were generated through the use of Ingenuity Pathway Analysis (IPA) and written permission was granted by QIAGEN Silicon Valley to use and adapt the figure generated by IPA under the terms of the Creative Commons Attribution License (CC BY) 4.0.

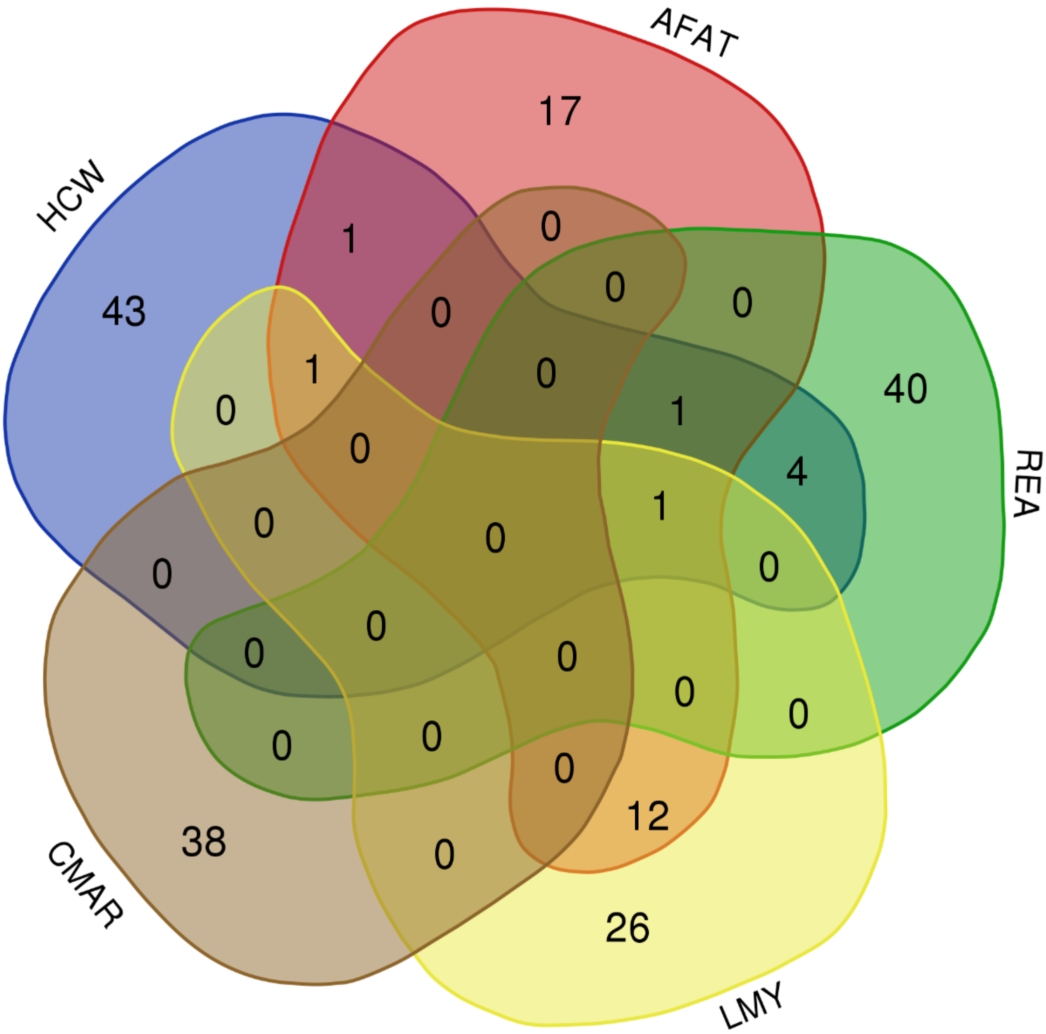
AFAT
HCW
REA
CMAR
LMY
17
1
0
43
0
0
0
1
0
0
1
40
0
0
0
1
4
0
0
0
0
0
0
0
0
0
0
0
0
0
0
12
38
0
26

a

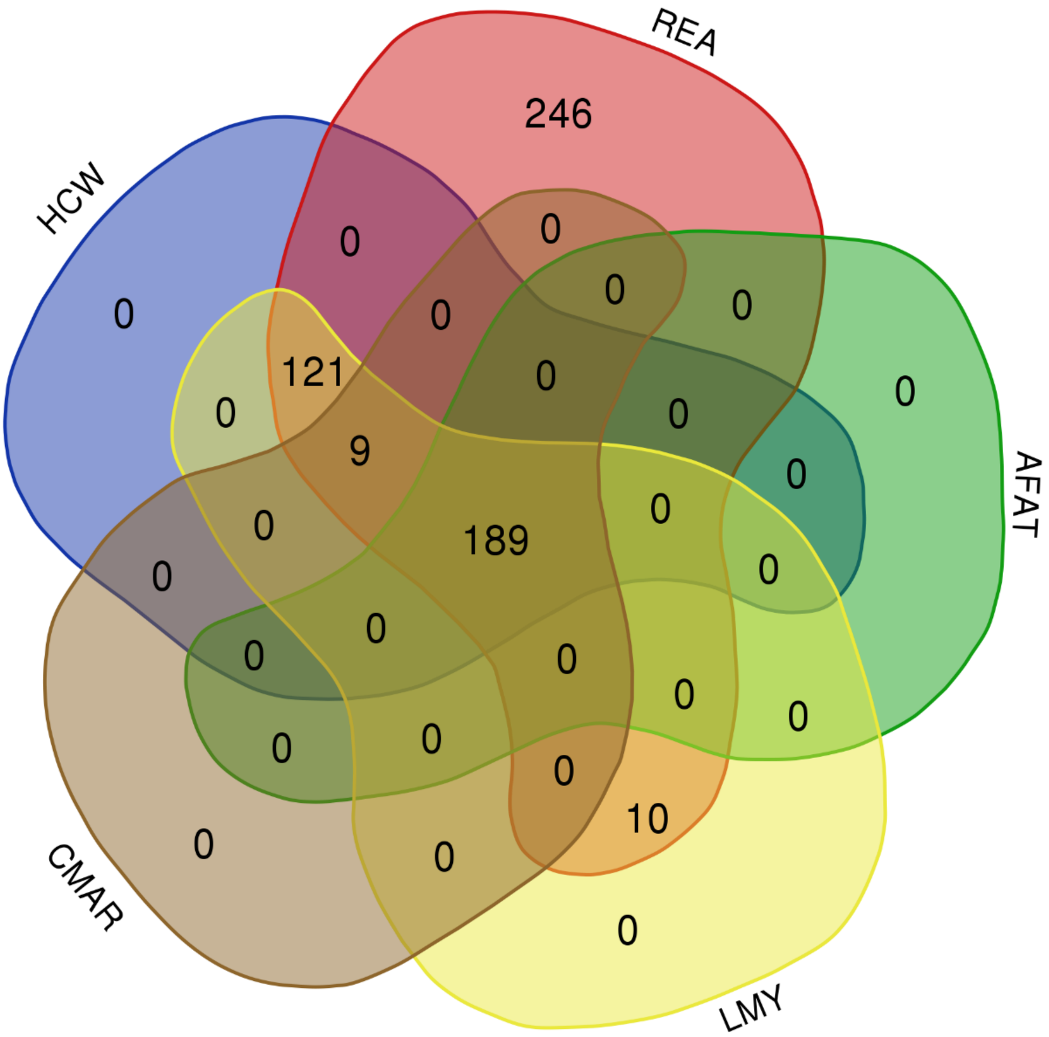


b

**Figure S4.** Venn diagram showing the overlapped lead significant SNPs (a) and candidate genes (b) among five carcass merit traits based on the imputed 7.8M DNA variant GWAS.
