# Supplementary material for: Genetic architecture of quantitative traits in beef cattle revealed by genome wide association studies of imputed whole genome sequence variants: II: carcass merit traits
Source: BMC Genomics. 2020 Jan 13;21:38. doi: 10.1186/s12864-019-6273-1 (PMC6958779; doi:10.1186/s12864-019-6273-1)
Supplement: Supplementary file 3 — Additional file 3: This file contains additional Tables including Table S1. Functional annotation of 7.8 M WGS variants along with the number of variants in each class, classification of SNP functions, percentage of WGS and 9 functional class assignments; Table S2. Functional annotation of 50 K SNP genotypes after quality control along with the number of variants in each class, classification of SNP functions, percentage of WGS and 9 functional class assignments; Table S3. SNP functional annotation of all DNA variants (38,318,974) based on DNA variants of the 1000 bulls genome project; Table S4. List of lead SNPs that were overlapped with QTLs published in the Cattle QTL database within 1 M bp up or 1 M bp downstream for HCW, AFAT, REA, LMY, and CMAR. [file 12864_2019_6273_MOESM3_ESM.pdf]

Table S1. Functional annotation of 7.8M WGS variants along with the number of variants in each class, classification of SNP functions, percentage of WGS and 9 functional class assignments

Functional class annotation	Number of SNPs	Classification of SNP functions	% of WGS	class
intergenic_region	5251680	intergenic_region	66.87%	1
downstream_gene_variant	253163	downstream_gene_variant	3.22%	2
upstream_gene_variant	285798	upstream_gene_variant	3.64%	3
non_coding_exon_variant	1953	synonymous_variant	0.41%	4
synonymous_variant	30066	synonymous_variant		4
intron_variant	1987366	intron_variant	25.31%	5
missense_variant	17654	missense_variant	0.22%	6
3’UTR_variant	15851	3’UTR_variant	0.20%	7
5’UTR_variant	2868	5’UTR_variant	0.04%	8
5’UTR_premature_start_codon_gain_variant	441	5’UTR_variant		8
splice_region_variant&intron_variant	4189	Other_regulatory	0.08%	9
missense_variant&splice_region_variant	470	Other_regulatory		9
splice_region_variant&synonymous_variant	664	Other_regulatory		9
inframe_deletion	98	Other_regulatory		9
frameshift_variant	150	Other_regulatory		9
splice_donor_variant&intron_variant	118	Other_regulatory		9
disruptive_inframe_deletion	52	Other_regulatory		9
splice_region_variant	141	Other_regulatory		9
splice_acceptor_variant&intron_variant	116	Other_regulatory		9
frameshift_variant&splice_donor_variant&splice_region_variant&intron_variant	3	Other_regulatory		9
splice_acceptor_variant&splice_donor_variant&intron_variant	19	Other_regulatory		9
splice_region_variant&non_coding_exon_variant	61	Other_regulatory		9

stop_retained_variant	19	Other_regulatory	9
frameshift_variant&splice_region_variant	13	Other_regulatory	9
stop_gained	161	Other_regulatory	9
splice_acceptor_variant&splice_region_variant&intron_variant	11	Other_regulatory	9
splice_region_variant&stop_retained_variant	3	Other_regulatory	9
stop_lost&splice_region_variant	9	Other_regulatory	9
start_lost	27	Other_regulatory	9
disruptive_inframe_insertion	7	Other_regulatory	9
inframe_insertion	13	Other_regulatory	9
frameshift_variant&stop_lost&splice_region_variant	1	Other_regulatory	9
stop_gained&splice_region_variant	9	Other_regulatory	9
stop_lost	3	Other_regulatory	9
frameshift_variant&splice_acceptor_variant&splice_region_variant&intron_variant	2	Other_regulatory	9
frameshift_variant&stop_gained	1	Other_regulatory	9
splice_donor_variant&splice_region_variant&intron_variant	5	Other_regulatory	9
stop_gained&inframe_insertion	1	Other_regulatory	9
initiator_codon_variant&non_canonical_start_codon	1	Other_regulatory	9
frameshift_variant&start_lost&splice_region_variant	1	Other_regulatory	9
frameshift_variant&stop_lost	1	Other_regulatory	9
frameshift_variant&start_lost	1	Other_regulatory	9
disruptive_inframe_deletion&splice_region_variant	1	Other_regulatory	9
Total	7853211		100.00%

Table S2. Functional annotation of 50K SNP genotypes after quality control along with the number of variants in each class, classification of SNP functions, percentage of WGS and 9 functional class assignments

Functional class annotation	Number of SNPs	Classification of SNP functions	% of WGS	class
intergenic_region	18313	intergenic_region	60.73%	1
downstream_gene_variant	1138	downstream_gene_variant	3.77%	2
upstream_gene_variant	1308	upstream_gene_variant	4.34%	3
non_coding_transcript_exon_variant	15	synonymous_variant	1.75%	4
synonymous_variant	513	synonymous_variant		4
intron_variant	8528	intron_variant	28.28%	5
missense_variant	68	missense_variant	0.23%	6
3’UTR_variant	193	3’UTR_variant	0.64%	7
5’UTR_premature_start_codon_gain_variant	4	5’UTR_variant	0.10%	8
5’UTR_variant	26	5’UTR_variant		8
initiator_codon_variant&non_canonical_start_codon	1	Other_regulatory	0.16%	9
missense_variant&splice_region_variant	3	Other_regulatory		9
splice_region_variant	1	Other_regulatory		9
splice_region_variant&intron_variant	27	Other_regulatory		9
splice_region_variant&non_coding_transcript_exon_variant	2	Other_regulatory		9
splice_region_variant&synonymous_variant	13	Other_regulatory		9
stop_retained_variant	2	Other_regulatory		9
total	30155		100.00%	

Table S3. SNP functional annotation of all DNA variants (38,318,974) based on DNA variants of the 1000 bulls genome project. The proportion was calculated as (numbers of SNPs in a class/ total SNPs/Indel)*100

SNP functional class	number of SNPs	Proportion
[1] "intergenic_region"	25179655	65.7%
[2] "downstream_gene_variant"	1393115	3.6%
[3] "non_coding_exon_variant"	10973	<0.1%
[4] "upstream_gene_variant"	1591843	4.2%
[5] "synonymous_variant"	155200	0.4%
[6] "missense_variant"	143708	0.4%
[7] "splice_region_variant&synonymous_variant"	3439	<0.1%
[8] "intron_variant"	9693772	25.3%
[9] "stop_gained"	3221	<0.1%
[10] "splice_region_variant&intron_variant"	23960	<0.1%
[11] "5’UTR_variant"	16109	<0.1%
[12] "3’UTR_variant"	88639	0.2%
[13] "splice_region_variant"	1038	<0.1%
[14] "splice_donor_variant&intron_variant"	1613	<0.1%
[15] "5’UTR_premature_start_codon_gain_variant"	2698	<0.1%
[16] "splice_acceptor_variant&intron_variant"	1419	<0.1%
[17] "missense_variant&splice_region_variant"	3949	<0.1%
[18] "inframe_deletion"	948	<0.1%
[19] "frameshift_variant"	1285	<0.1%
[20] "inframe_insertion"	131	<0.1%

[21] "splice_region_variant&non_coding_exon_variant"	426	<0.1%
[22] "disruptive_inframe_deletion"	529	<0.1%
[23] "frameshift_variant&stop_gained"	17	<0.1%
[24] "frameshift_variant&splice_region_variant"	133	<0.1%
[25] "stop_gained&splice_region_variant"	152	<0.1%
[26] "start_lost"	168	<0.1%
[27] "splice_acceptor_variant&splice_donor_variant&intron_variant"	180	<0.1%
[28] "disruptive_inframe_insertion"	76	<0.1%
[29] "splice_donor_variant&splice_region_variant&intron_variant"	88	<0.1%
[30] "splice_acceptor_variant&splice_region_variant&intron_variant"	105	<0.1%
[31] "stop_retained_variant"	71	<0.1%
[32] "disruptive_inframe_deletion&splice_region_variant"	11	<0.1%
[33] "frameshift_variant&splice_acceptor_variant&splice_region_variant&intron_variant"	20	<0.1%
[34] "stop_lost&splice_region_variant"	47	<0.1%
[35]"frameshift_variant&splice_donor_variant&splice_region_variant&intron_variant"	28	<0.1%
[36] "stop_lost"	76	<0.1%
[37] "splice_region_variant&downstream_gene_variant"	1	<0.1%
[38] "splice_region_variant&stop_retained_variant"	34	<0.1%
[39] "inframe_deletion&splice_region_variant"	10	<0.1%
[40] "initiator_codon_variant"	19	<0.1%

[41] "splice_donor_variant&disruptive_inframe_deletion&splice_region_variant&intron_variant"	2	<0.1%
[42] "disruptive_inframe_insertion&splice_region_variant"	1	<0.1%
[43] "frameshift_variant&stop_lost"	8	<0.1%
[44] "splice_acceptor_variant&splice_region_variant&intron_variant&non_coding_exon_variant"	1	<0.1%
[45] "splice_acceptor_variant&inframe_deletion&splice_region_variant&intron_variant"	5	<0.1%
[46] "splice_acceptor_variant&5’UTR_variant&intron_variant"	2	<0.1%
[47] "splice_donor_variant&inframe_deletion&splice_region_variant&intron_variant"	5	<0.1%
[48] "inframe_insertion&splice_region_variant"	3	<0.1%
[49] "frameshift_variant&stop_lost&splice_region_variant"	4	<0.1%
[50] "stop_gained&disruptive_inframe_deletion"	1	<0.1%
[51] "start_lost&splice_region_variant"	2	<0.1%
[52] "splice_acceptor_variant&disruptive_inframe_deletion&splice_region_variant&intron_variant"	2	<0.1%
[53] "frameshift_variant&start_lost"	7	<0.1%
[54] "stop_gained&inframe_insertion"	5	<0.1%

[55] "frameshift_variant&splice_acceptor_variant&splice_donor_variant&splice_region_variant&intron_variant"	3	<0.1%
[56] "exon_loss_variant&splice_acceptor_variant&splice_donor_variant&splice_region_variant&intron_variant"	2	<0.1%
[57] "start_lost&inframe_insertion"	1	<0.1%
[58] "start_lost&inframe_deletion"	2	<0.1%
[59] "initiator_codon_variant&non_canonical_start_codon"	2	<0.1%
[60] "splice_donor_variant&3’UTR_variant&intron_variant"	1	<0.1%
[61] "stop_gained&disruptive_inframe_insertion"	1	<0.1%
[62] "splice_acceptor_variant&splice_region_variant&3’UTR_variant&intron_variant"	1	<0.1%
[63] "frameshift_variant&start_lost&splice_region_variant"	1	<0.1%
[64] "stop_gained&disruptive_inframe_deletion&splice_region_variant"	1	<0.1%
[65] "frameshift_variant&stop_gained&splice_region_variant"	2	<0.1%
[66] "splice_donor_variant&5’UTR_variant&intron_variant"	1	<0.1%
[67] "splice_donor_variant&splice_region_variant&3’UTR_variant&intron_variant"	1	<0.1%
[68] "splice_acceptor_variant&splice_region_variant&5’UTR_variant&intron_variant"	1	<0.1%
Total SNP and Indel	38,318,974	

Table S4. List of lead SNPs that were overlapped with QTLs published in the Cattle QTL database within 1M bp up or 1M bp downstream for HCW, AFAT, REA, LMY, CMAR, where the last column shows a list of QTL ids with reference numbers in square brackets. Overlapped QTLs within 70k bp up or 70k bp downstream were highlighted in bold. The references were provided at the end of the file.

Trait	SNP	Chr	bp	No. of Overlap QTLs	Reference (qtl_id[reference])
HCW	rs109658371	6	39213566	19	107737[10], 24250[21], 24620[17], 21177[24], 21183[24], 20103[30], 20106[30], 20109[30], 20115[30], 20118[30], 20121[30], 20124[30], 24253[21], 20127[30], 24254[21], 20130[30], **24623**[17], **24255**[21], 21179[24]
HCW	Chr6:39111019	6	39111019	20	107737[10], 21175[24], 24250[21], 24620[17], 21177[24], 21183[24], 20103[30], 20106[30], 20109[30], 20115[30], 20118[30], 20121[30], 20124[30], 24253[21], 20127[30], 24254[21], **20130**[30], **24623**[17], 24255[21], 21179[24]
HCW	rs109815800	14	25015640	10	107727[10], **122423**[6], **122457**[4], 24632[17], 20862[26], 20863[26], 20864[26], 20865[26], **24634**[17], 21188[24]
HCW	rs110001259	6	39026381	21	107737[10], 25131[18], 21175[24], 24250[21], **24620**[17], 21177[24], 21183[24], 20103[30], 20106[30], 20109[30], 20115[30], 20118[30], 20121[30], 20124[30], 24253[21], **20127**[30], **24254**[21], **20130**[30], 24623[17], 24255[21], 21179[24]
HCW	rs109843602	6	38750035	21	**107737**[10], 21174[24], 25131[18], 21175[24], 24250[21], **24620**[17], 21177[24], **21183**[24], **20103**[30], **20106**[30], **20109**[30], **20115**[30], **20118**[30], **20121**[30], **20124**[30], **24253**[21], 20127[30], 24254[21], 20130[30], 24623[17], 24255[21]
HCW	rs109696064	6	38837730	21	**107737**[10], 25131[18], 21175[24], 24250[21], **24620**[17], 21177[24], **21183**[24], **20103**[30], **20106**[30], **20109**[30], **20115**[30], **20118**[30], **20121**[30], **20124**[30], **24253**[21], **20127**[30], **24254**[21], 20130[30], 24623[17], 24255[21], 21179[24]
HCW	rs110995268	6	38914196	21	**107737**[10], 25131[18], 21175[24], 24250[21], **24620**[17], 21177[24], 21183[24], 20103[30], 20106[30], **20109**[30], **20115**[30], **20118**[30], **20121**[30], **20124**[30], **24253**[21], **20127**[30], **24254**[21], **20130**[30], 24623[17], 24255[21], 21179[24]
HCW	rs378208925	6	39449045	19	107737[10], 24250[21], 24620[17], 21177[24], 21183[24], 20103[30], 20106[30], 20109[30], 20115[30], 20118[30], 20121[30], 20124[30], 24253[21], 20127[30], 24254[21], 20130[30], 24623[17], 24255[21], 21179[24]
HCW	rs109355965	6	38657124	21	**107737**[10], 21174[24], 25131[18], 21175[24], 24250[21], **24620**[17], **21177**[24], **21183**[24], **20103**[30], **20106**[30], **20109**[30], **20115**[30], 20118[30], 20121[30], 20124[30], 24253[21], 20127[30], 24254[21], 20130[30], 24623[17], 24255[21]
HCW	rs109919489	6	39357241	19	107737[10], 24250[21], 24620[17], 21177[24], 21183[24], 20103[30], 20106[30], 20109[30], 20115[30], 20118[30], 20121[30], 20124[30], 24253[21], 20127[30], 24254[21], 20130[30], 24623[17], **24255**[21], 21179[24]

HCW	rs110395441	14	24930535	9	107727[10], **122423**[6], **122457**[4], 24632[17], 20862[26], 20863[26], 20864[26], 20865[26], 24634[17]
HCW	rs382462900	6	38429007	22	107737[10], 21173[24], 21174[24], 25131[18], 21175[24], **24250**[21], **24620**[17], 21177[24], 21183[24], 20103[30], 20106[30], 20109[30], 20115[30], 20118[30], 20121[30], 20124[30], 24253[21], 20127[30], 24254[21], 20130[30], 24623[17], 24255[21]
HCW	rs41934045	20	4563925	2	**24639**[17], 24642[17]
HCW	rs383638885	6	38031945	21	107737[10], 24249[21], 21173[24], 21174[24], **25131**[18], **21175**[24], 24250[21], **24620**[17], 21177[24], 21183[24], 20103[30], 20106[30], 20109[30], 20115[30], 20118[30], 20121[30], 20124[30], 24253[21], 20127[30], 24254[21], 20130[30]
HCW	rs43349810	20	4637730	2	**24639**[17], 24642[17]
HCW	rs109278547	6	39579598	17	107737[10], 24620[17], 21183[24], 20103[30], 20106[30], 20109[30], 20115[30], 20118[30], 20121[30], 20124[30], 24253[21], 20127[30], 24254[21], 20130[30], 24623[17], 24255[21], 21179[24]
HCW	rs209174889	14	25565128	14	107727[10], 122423[6], 122457[4], 24632[17], 20862[26], 20863[26], 20864[26], 20865[26], **24634**[17], 21188[24], 56744[11], 56745[11], 24637[17], 56763[11]
HCW	rs383473904	6	37872592	18	107737[10], 24249[21], 21173[24], **21174**[24], 25131[18], 21175[24], 24250[21], 24620[17], 21177[24], 21183[24], 20103[30], 20106[30], 20109[30], 20115[30], 20118[30], 20121[30], 20124[30], 24253[21]
HCW	rs210353492	6	38107063	21	107737[10], 24249[21], 21173[24], 21174[24], **25131**[18], **21175**[24], 24250[21], **24620**[17], 21177[24], 21183[24], 20103[30], 20106[30], 20109[30], 20115[30], 20118[30], 20121[30], 20124[30], 24253[21], 20127[30], 24254[21], 20130[30],
HCW	rs385289515	6	38509747	21	107737[10], 21174[24], 25131[18], 21175[24], **24250**[21], **24620**[17], **21177**[24], 21183[24], 20103[30], 20106[30], 20109[30], 20115[30], 20118[30], 20121[30], 20124[30], 24253[21], 20127[30], 24254[21], 20130[30], 24623[17], 24255[21]
HCW	rs43349745	20	4762743	2	**24639**[17], 24642[17]
HCW	rs41574252	20	4863507	2	**24639**[17], 24642[17]
HCW	rs210782610	7	93205703	1	**24625**[17]
HCW	rs211486269	14	24508890	11	107727[10], 122423[6], 122457[4], 24631[17], 36546[16], **24632**[17], 20862[26], 20863[26], 20864[26], 20865[26], 24634[17]
HCW	rs42646728	14	24603039	11	107727[10], 122423[6], 122457[4], 24631[17], 36546[16], **24632**[17], 20862[26], 20863[26], 20864[26], 20865[26], 24634[17]
HCW	rs136750548	6	38583783	21	107737[10], 21174[24], 25131[18], 21175[24], **24250**[21], **24620**[17], **21177**[24], **21183**[24], 20103[30], 20106[30], 20109[30], 20115[30], 20118[30], 20121[30], 20124[30], 24253[21], 20127[30], 24254[21], 20130[30], 24623[17], 24255[21]
HCW	rs382496844	6	39678846	17	107737[10], 24620[17], 21183[24], 20103[30], 20106[30], 20109[30], 20115[30], 20118[30], 20121[30], 20124[30], 24253[21], 20127[30], 24254[21], 20130[30], 24623[17], 24255[21], 21179[24]

HCW	rs109155034	7	94219428	1	24625[17]
HCW	rs378603811	6	39960847	4	20130[30], 24623[17], 24255[21], **21179**[24]
HCW	rs209687009	7	93019197	1	**24625**[17]
HCW	rs800258890	6	34709055	4	24618[17], 21170[24], 21171[24], 21172[24]
HCW	Chr6:37959886	6	37959886	20	107737[10], 24249[21], 21173[24], 21174[24], **25131**[18], 21175[24], 24250[21], **24620**[17], 21177[24], 21183[24], 20103[30], 20106[30], 20109[30], 20115[30], 20118[30], 20121[30], 20124[30], 24253[21], 20127[30], 24254[21]
HCW	rs380987034	6	53934503	1	**24624**[17]
HCW	rs378475486	7	92894777	1	**24625**[17]
HCW	rs446018954	6	43386058	1	**20358**[29]
HCW	Chr14:25458973	14	25458973	13	107727[10], 122423[6], **122457**[4], 24632[17], 20862[26], 20863[26], 20864[26], 20865[26], **24634**[17], 21188[24], 56744[11], 56745[11], 56763[11]
HCW	rs109060535	14	26223578	16	107727[10], 122457[4], 20862[26], 20863[26], 20864[26], 20865[26], 24634[17], 21188[24], **56744**[11], **56745**[11], 24637[17], 23266[20], **56763**[11], 56747[11], 23268[20], 56764[11]
HCW	rs209393630	6	42510243	1	20358[29]
HCW	rs42892600	14	25329035	13	**107727**[10], 122423[6], **122457**[4], 24632[17], **20862**[26], **20863**[26], **20864**[26], **20865**[26], **24634**[17], 21188[24], 56744[11], 56745[11], 56763[11]
HCW	rs134134773	6	43976500	1	20358[29]
HCW	rs135023213	14	24739872	11	107727[10], 122423[6], **122457**[4], 24631[17], 36546[16], **24632**[17], 20862[26], 20863[26], 20864[26], 20865[26], 24634[17]
AFAT	rs110995268	6	38914196	12	**24647**[17], **24648**[17], 20102[30], 20105[30], **20108**[30], **20111**[30], **20114**[30], **20117**[30], **20120**[30], **20123**[30], **20126**[30], **20129**[30]
AFAT	rs109843602	6	38750035	12	**24647**[17], **24648**[17], **20102**[30], **20105**[30], **20108**[30], **20111**[30], **20114**[30], **20117**[30], **20120**[30], **20123**[30], 20126[30], 20129[30]
AFAT	rs380838173	6	39120384	12	**24647**[17], 24648[17], 20102[30], 20105[30], 20108[30], 20111[30], 20114[30], 20117[30], 20120[30], 20123[30], 20126[30], **20129**[30]
AFAT	rs109029403	6	39008672	12	**24647**[17], **24648**[17], 20102[30], 20105[30], 20108[30], 20111[30], 20114[30], 20117[30], 20120[30], 20123[30], **20126**[30], **20129**[30]

AFAT	rs109696064	6	38837730	12	**24647**[17], **24648**[17], **20102**[30], **20105**[30], **20108**[30], **20111**[30], **20114**[30], **20117**[30], **20120**[30], **20123**[30], **20126**[30], 20129[30]
AFAT	rs109355965	6	38657124	12	**24647**[17], **24648**[17], **20102**[30], **20105**[30], **20108**[30], **20111**[30], **20114**[30], 20117[30], 20120[30], 20123[30], 20126[30], 20129[30]
AFAT	rs109927583	6	39211262	12	**24647**[17], 24648[17], 20102[30], 20105[30], 20108[30], 20111[30], 20114[30], 20117[30], 20120[30], 20123[30], 20126[30], 20129[30]
AFAT	rs109515116	6	39445899	12	**24647**[17], 24648[17], 20102[30], 20105[30], 20108[30], 20111[30], 20114[30], 20117[30], 20120[30], 20123[30], 20126[30], 20129[30]
AFAT	rs110747048	6	39353897	12	**24647**[17], 24648[17], 20102[30], 20105[30], 20108[30], 20111[30], 20114[30], 20117[30], 20120[30], 20123[30], 20126[30], 20129[30]
AFAT	rs381910687	16	24333881	2	122446[4], 56154[12]
AFAT	rs379496842	14	25350856	13	126466[3], 126467[3], **126468**[3], 57476[13], 36553[16], 20854[26], **20855**[26], **20856**[26], **20857**[26], **20858**[26], **20859**[26], **20860**[26], **20861**[26]
AFAT	rs209930593	13	63970531	1	**122445**[4]
AFAT	rs137160543	11	92616956	1	20284[29]
AFAT	rs134958846	14	24894463	13	126466[3], **126467**[3], 126468[3], 57476[13], **36553**[16], 20854[26], 20855[26], 20856[26], 20857[26], 20858[26], 20859[26], 20860[26], 20861[26]
AFAT	Chr16:24532207	16	24532207	2	122446[4], 56154[12]
AFAT	rs109638708	17	62670312	1	**24654**[17]
AFAT	rs211327835	13	64067399	1	**122445**[4]
AFAT	Chr13:64539970	13	64539970	1	**122445**[4]
AFAT	rs382683307	6	39693004	12	**24647**[17], 24648[17], 20102[30], 20105[30], 20108[30], 20111[30], 20114[30], 20117[30], 20120[30], 20123[30], 20126[30], 20129[30]
AFAT	rs133531965	17	62789778	1	**24654**[17]
REA	rs109658371	6	39213566	11	24697[17], 20104[30], 20107[30], 20110[30], 20113[30], 20116[30], 20119[30], 20122[30], 20125[30], 20128[30], 20131[30]
REA	rs378858709	6	39020582	11	**24697**[17], 20104[30], 20107[30], 20110[30], 20113[30], 20116[30], 20119[30], 20122[30], 20125[30], **20128**[30], **20131**[30]

REA	rs109843602	6	38750035	11	**24697**[17], **20104**[30], **20107**[30], **20110**[30], **20113**[30], **20116**[30], **20119**[30], **20122**[30], **20125**[30], 20128[30], 20131[30]
REA	rs109256415	6	38911548	11	**24697**[17], 20104[30], **20107**[30], **20110**[30], **20113**[30], **20116**[30], **20119**[30], **20122**[30], **20125**[30], **20128**[30], **20131**[30]
REA	rs109331793	6	39117869	11	24697[17], 20104[30], 20107[30], 20110[30], 20113[30], 20116[30], 20119[30], 20122[30], 20125[30], 20128[30], **20131**[30]
REA	rs109696064	6	38837730	11	**24697**[17], **20104**[30], **20107**[30], **20110**[30], **20113**[30], **20116**[30], **20119**[30], **20122**[30], **20125**[30], **20128**[30], 20131[30]
REA	rs110766531	6	38670165	11	**24697**[17], **20104**[30], **20107**[30], **20110**[30], **20113**[30], **20116**[30], **20119**[30], **20122**[30], 20125[30], 20128[30], 20131[30]
REA	rs210984648	6	39414353	11	24697[17], 20104[30], 20107[30], 20110[30], 20113[30], 20116[30], 20119[30], 20122[30], 20125[30], 20128[30], 20131[30]
REA	rs379796058	6	38035891	11	**24697**[17], 20104[30], 20107[30], 20110[30], 20113[30], 20116[30], 20119[30], 20122[30], 20125[30], 20128[30], 20131[30]
REA	rs385289515	6	38509747	11	**24697**[17], 20104[30], 20107[30], 20110[30], 20113[30], 20116[30], 20119[30], 20122[30], 20125[30], 20128[30], 20131[30]
REA	rs110254070	6	38127508	11	**24697**[17], 20104[30], 20107[30], 20110[30], 20113[30], 20116[30], 20119[30], 20122[30], 20125[30], 20128[30], 20131[30]
REA	rs109645115	6	39580784	11	24697[17], 20104[30], 20107[30], 20110[30], 20113[30], 20116[30], 20119[30], 20122[30], 20125[30], 20128[30], 20131[30]
REA	Chr6:39494659	6	39494659	11	24697[17], 20104[30], 20107[30], 20110[30], 20113[30], 20116[30], 20119[30], 20122[30], 20125[30], 20128[30], 20131[30]
REA	rs135364450	6	38589385	11	**24697**[17], 20104[30], 20107[30], 20110[30], 20113[30], 20116[30], 20119[30], 20122[30], 20125[30], 20128[30], 20131[30]
REA	rs110328189	7	94024137	1	**24699**[17]
REA	rs109901274	7	93244933	1	**24699**[17]
REA	rs382462900	6	38429007	11	**24697**[17], 20104[30], 20107[30], 20110[30], 20113[30], 20116[30], 20119[30], 20122[30], 20125[30], 20128[30], 20131[30]
REA	rs110114429	7	93148272	1	**24699**[17]
REA	rs381550243	6	37942057	10	**24697**[17], 20104[30], 20107[30], 20110[30], 20113[30], 20116[30], 20119[30], 20122[30], 20125[30], 20128[30]
REA	rs380628392	6	39651414	11	24697[17], 20104[30], 20107[30], 20110[30], 20113[30], 20116[30], 20119[30], 20122[30], 20125[30], 20128[30], 20131[30]
REA	rs378955293	6	37871185	9	24697[17], 20104[30], 20107[30], 20110[30], 20113[30], 20116[30], 20119[30], 20122[30], 20125[30]
REA	rs135551190	14	24977053	2	**122424**[6], 20866[26]
REA	rs478618528	2	5731782	1	24689[17]
REA	rs110874471	2	6210115	1	**24689**[17]
REA	rs42447757	6	39342689	11	24697[17], 20104[30], 20107[30], 20110[30], 20113[30], 20116[30], 20119[30], 20122[30], 20125[30], 20128[30], 20131[30]
REA	rs110109692	7	93562806	1	**24699**[17]
REA	rs109534801	6	39985654	1	20131[30]

REA	Chr9:98315390	9	98315390	1	20342[29]
REA	rs209687009	7	93019197	1	**24699**[17]
REA	rs110233015	2	6672584	1	**24689**[17]
REA	rs385433684	7	93431326	1	**24699**[17]
LMY	rs41594006	4	28702952	1	36852[15]
LMY	rs134653419	3	60675816	1	**37224**[15]
LMY	rs383507504	1	58167076	3	36851[15], 37015[15], 37064[15]
LMY	rs446447694	4	28218153	1	36852[15]
LMY	rs110144484	13	64005097	2	36809[15], 36805[15]
LMY	rs381333982	4	28338381	1	36852[15]
LMY	rs109722048	7	94363721	1	36732[15]
LMY	rs209255508	20	36664583	10	37022[15], 36894[15], 36839[15], 36877[15], 36876[15], 36929[15], 36713[15], 36904[15], 37207[15], 36982[15]
LMY	rs443713949	6	55877885	8	36806[15], 36705[15], 36717[15], 36719[15], 36714[15], 36722[15], 36964[15], 37067[15]
LMY	Chr20:36592776	20	36592776	10	37022[15], 36894[15], 36839[15], 36877[15], 36876[15], 36929[15], 36713[15], 36904[15], 37207[15], 36982[15]
LMY	rs41704822	13	64131777	2	36809[15], 36805[15]
LMY	rs211128418	20	36504244	10	37022[15], **36894**[15], 36839[15], 36877[15], 36876[15], 36929[15], 36713[15], 36904[15], 37207[15], 36982[15]
LMY	rs379496842	14	25350856	1	37196[15]
LMY	rs470267387	4	28143718	1	36852[15]
LMY	rs210604912	20	37309100	12	36894[15], **36839**[15], **36877**[15], **36876**[15], **36929**[15], **36713**[15], **36904**[15], 37207[15], 36982[15], 37136[15], 37228[15], 36739[15]
CMAR	rs208356852	5	1.12E+08	1	20303[29]
CMAR	rs464116897	1	33921239	1	20295[29]

List of references for QTLs in CattleQTL DB for five carcass merit traits

Reference number	Title	Description	Details	ShortDetails	Identifiers
1	The genetic and biological basis of feed efficiency in mid-lactation Holstein dairy cows.	Hardie LC, VandeHaar MJ, Tempelman RJ, Weigel KA, Armentano LE, Wiggans GR, Veerkamp RF, de Haas Y, Coffey MP, Connor EE, Hanigan MD, Staples C, Wang Z, Dekkers JCM, Spurlock DM.	J Dairy Sci. 2017 Nov;100(11):9061-9075. doi: 10.3168/jds.2017-12604. Epub 2017 Aug 23.	J Dairy Sci. 2017	PMID:28843688
2	Genome-wide association study for feed efficiency and growth traits in U.S. beef cattle.	Seabury CM, Oldeschulte DL, Saatchi M, Beever JE, Decker JE, Halley YA, Bhattarai EK, Molaei M, Freetly HC, Hansen SL, Yampara-Iquise H, Johnson KA, Kerley MS, Kim J, Loy DD, Marques E, Neibergs HL, Schnabel RD, Shike DW, Spangler ML, Weaber RL, Garrick DJ, et al.	BMC Genomics. 2017 May 18;18(1):386. doi: 10.1186/s12864-017-3754-y.	BMC Genomics. 2017	PMID:28521758
3	Genome-Wide Association Study for Carcass Traits in an Experimental Nelore Cattle Population.	Medeiros de Oliveira Silva R, Bonvino Stafuzza N, de Oliveira Fragomeni B, Miguel Ferreira de Camargo G, Matos Ceacero T, Noely Dos Santos GonÃ§alves Cyrillo J, Baldi F, Augusti Boligon A, Zerlotti Mercadante ME, Lino Lourenco D, Misztal I, GalvÃ£o de Albuquerque L.	PLoS One. 2017 Jan 24;12(1):e0169860. doi: 10.1371/journal.pone.0169860. eCollection 2017.	PLoS One. 2017	PMID:28118362

4	Genome scan for postmortem carcass traits in Nellore cattle.	JÃºnior GA, Costa RB, de Camargo GM, Carvalheiro R, Rosa GJ, Baldi F, Garcia DA, Gordo DG, Espigolan R, Takada L, MagalhÃ£es AF, Bresolin T, Feitosa FL, Chardulo LA, de Oliveira HN, de Albuquerque LG.	J Anim Sci. 2016 Oct;94(10):4087-4095. doi: 10.2527/jas.2016-0632.	J Anim Sci. 2016	PMID:27898882
5	Multi-strategy genome-wide association studies identify the DCAF16-NCAPG region as a susceptibility locus for average daily gain in cattle.	Zhang W, Li J, Guo Y, Zhang L, Xu L, Gao X, Zhu B, Gao H, Ni H, Chen Y.	Sci Rep. 2016 Nov 28;6:38073. doi: 10.1038/srep38073.	Sci Rep. 2016	PMID:27892541
6	Genetic association of PLAG1, SCD, CYP7B1 and FASN SNPs and their effects on carcass weight, intramuscular	Kim HJ, Sharma A, Lee SH, Lee DH, Lim DJ, Cho YM, Yang BS, Lee SH.	Anim Genet. 2017 Apr;48(2):251-252. doi: 10.1111/age.12523. Epub 2016 Nov 22. No abstract available.	Anim Genet. 2017	PMID:27878829

	fat and fatty acid composition in Hanwoo steers (Korean cattle).				
7	Genomic Regions Associated with Feed Efficiency Indicator Traits in an Experimental Nellore Cattle Population.	Olivieri BF, Mercadante ME, Cyrillo JN, Branco RH, Bonilha SF, de Albuquerque LG, Silva RM, Baldi F.	PLoS One. 2016 Oct 19;11(10):e0164390. doi: 10.1371/journal.pone.0164390. eCollection 2016. Erratum in: PLoS One. 2017 Feb 6;12 (2):e0171845.	PLoS One. 2016	PMID:27760167
8	Genome wide association study on beef production traits in Marchigiana cattle breed.	Sorbolini S, Bongiorni S, Cellesi M, Gaspa G, Dimauro C, Valentini A, Macciotta NP.	J Anim Breed Genet. 2017 Feb;134(1):43-48. doi: 10.1111/jbg.12227. Epub 2016 Jun 22.	J Anim Breed Genet. 2017	PMID:27329851
9	Genome-wide association studies of	Mao X, Sahana G, De Koning DJ, Guldbrandtsen B.	J Anim Sci. 2016 Apr;94(4):1426-37. doi: 10.2527/jas.2015-9838.	J Anim Sci. 2016	PMID:27136002

	growth traits in three dairy cattle breeds using whole-genome sequence data.				
10	Genome-wide association study for carcass traits, fatty acid composition, chemical composition, sugar, and the effects of related candidate genes in Japanese Black cattle.	Sasago N, Abe T, Sakuma H, Kojima T, Uemoto Y.	Anim Sci J. 2017 Jan;88(1):33-44. doi: 10.1111/asj.12595. Epub 2016 Apr 25.	Anim Sci J. 2017	PMID:27112906
11	Multiple Linkage Disequilibrium Mapping Methods to	Li Y, Kim JJ.	Asian-Australas J Anim Sci. 2015 Jul;28(7):926-35. doi: 10.5713/ajas.15.0077.	Asian-Australas J Anim Sci. 2015	PMID:26104396

	Validate Additive Quantitative Trait Loci in Korean Native Cattle (Hanwoo).				
12	A genomewide association mapping study using ultrasound-scanned information identifies potential genomic regions and candidate genes affecting carcass traits in Nellore cattle.	Santana MH, Ventura RV, Utsunomiya YT, Neves HH, Alexandre PA, Oliveira Junior GA, Gomes RC, Bonin MN, Coutinho LL, Garcia JF, Silva SL, Fukumasu H, Leme PR, Ferraz JB.	J Anim Breed Genet. 2015 Dec;132(6):420-7. doi: 10.1111/jbg.12167. Epub 2015 May 27.	J Anim Breed Genet. 2015	PMID:26016521
13	Additive and epistatic genome-wide association for	Ali AA, Khatkar MS, Kadarmideen HN, Thomson PC.	J Anim Breed Genet. 2015 Apr;132(2):187-97. doi: 10.1111/jbg.12147. Epub 2015 Mar 6.	J Anim Breed Genet. 2015	PMID:25754883

	growth and ultrasound scan measures of carcass-related traits in Brahman cattle.				
14	QTLs associated with dry matter intake, metabolic mid-test weight, growth and feed efficiency have little overlap across 4 beef cattle studies.	Saatchi M, Beever JE, Decker JE, Faulkner DB, Freetly HC, Hansen SL, Yampara-Iquise H, Johnson KA, Kachman SD, Kerley MS, Kim J, Loy DD, Marques E, Neibergs HL, Pollak EJ, Schnabel RD, Seabury CM, Shike DW, Snelling WM, Spangler ML, Weaber RL, Garrick DJ, et al.	BMC Genomics. 2014 Nov 20;15:1004. doi: 10.1186/1471-2164-15-1004.	BMC Genomics. 2014	PMID:25410110
15	Whole genome association study identifies regions of the bovine genome and biological pathways	Doran AG, Berry DP, Creevey CJ.	BMC Genomics. 2014 Oct 1;15:837. doi: 10.1186/1471-2164-15-837.	BMC Genomics. 2014	PMID:25273628

	involved in carcass trait performance in Holstein-Friesian cattle.				
16	Validation of genetic polymorphisms on BTA14 associated with carcass trait in a commercial Hanwoo population.	Sharma A, Dang CG, Kim KS, Kim JJ, Lee HK, Kim HC, Yeon SH, Kang HS, Lee SH.	Anim Genet. 2014 Dec;45(6):863-7. doi: 10.1111/age.12204. Epub 2014 Aug 28.	Anim Genet. 2014	PMID:25164077
17	Large-effect pleiotropic or closely linked QTL segregate within and across ten US cattle breeds.	Saatchi M, Schnabel RD, Taylor JF, Garrick DJ.	BMC Genomics. 2014 Jun 6;15:442. doi: 10.1186/1471-2164-15-442.	BMC Genomics. 2014	PMID:24906442
18	Single nucleotide polymorphisms for feed efficiency and	Abo-Ismail MK, Vander Voort G, Squires JJ, Swanson KC, Mandell IB, Liao X, Stothard P, Moore S, Plastow G, Miller SP.	BMC Genet. 2014 Jan 30;15:14. doi: 10.1186/1471-2156-15-14.	BMC Genet. 2014	PMID:24476087

	performance in crossbred beef cattle.				
19	Bivariate genome-wide association analysis of the growth and intake components of feed efficiency.	SerÃ£o NV, GonzÃ¡lez-PeÃ±a D, Beever JE, Bollero GA, Southey BR, Faulkner DB, Rodriguez-Zas SL.	PLoS One. 2013 Oct 29;8(10):e78530. doi: 10.1371/journal.pone.0078530. eCollection 2013.	PLoS One. 2013	PMID:24205251
20	Genome-wide association study identifies major loci for carcass weight on BTA14 in Hanwoo (Korean cattle).	Lee SH, Choi BH, Lim D, Gondro C, Cho YM, Dang CG, Sharma A, Jang GW, Lee KT, Yoon D, Lee HK, Yeon SH, Yang BS, Kang HS, Hong SK.	PLoS One. 2013 Oct 7;8(10):e74677. doi: 10.1371/journal.pone.0074677. eCollection 2013.	PLoS One. 2013	PMID:24116007
21	Genome-wide association analyses for carcass quality in crossbred beef cattle.	Lu D, Sargolzaei M, Kelly M, Vander Voort G, Wang Z, Mandell I, Moore S, Plastow G, Miller SP.	BMC Genet. 2013 Sep 11;14:80. doi: 10.1186/1471-2156-14-80.	BMC Genet. 2013	PMID:24024930

22	Genome-wide association analyses for growth and feed efficiency traits in beef cattle.	Lu D, Miller S, Sargolzaei M, Kelly M, Vander Voort G, Caldwell T, Wang Z, Plastow G, Moore S.	J Anim Sci. 2013 Aug;91(8):3612-33. doi: 10.2527/jas.2012-5716. Epub 2013 Jul 12.	J Anim Sci. 2013	PMID:23851991
23	Bayesian genome-wide association analysis of growth and yearling ultrasound measures of carcass traits in Brangus heifers.	Peters SO, Kizilkaya K, Garrick DJ, Fernando RL, Reecy JM, Weaber RL, Silver GA, Thomas MG.	J Anim Sci. 2012 Oct;90(10):3398-409. doi: 10.2527/jas.2012-4507. Erratum in: J Anim Sci. 2013 Mar;91(3):1522.	J Anim Sci. 2012	PMID:23038745
24	Genome-wide association study identified three major QTL for carcass weight including the PLAG1-	Nishimura S, Watanabe T, Mizoshita K, Tatsuda K, Fujita T, Watanabe N, Sugimoto Y, Takasuga A.	BMC Genet. 2012 May 20;13:40. doi: 10.1186/1471-2156-13-40.	BMC Genet. 2012	PMID:22607022

	CHCHD7 QTN for stature in Japanese Black cattle.				
25	Genetic variation in PLAG1 associates with early life body weight and peripubertal weight and growth in Bos taurus.	Littlejohn M, Grala T, Sanders K, Walker C, Waghorn G, Macdonald K, Coppieters W, Georges M, Spelman R, Hillerton E, Davis S, Snell R.	Anim Genet. 2012 Oct;43(5):591-4. doi: 10.1111/j.1365-2052.2011.02293.x. Epub 2011 Dec 7.	Anim Genet. 2012	PMID:22497486
26	Genetic markers on BTA14 predictive for residual feed intake in beef steers and their effects on carcass and meat quality traits.	Lindholm-Perry AK, Kuehn LA, Snelling WM, Smith TP, Ferrell CL, Jenkins TG, King DA, Shackelford SD, Wheeler TL, Freetly HC.	Anim Genet. 2012 Oct;43(5):599-603. doi: 10.1111/j.1365-2052.2011.02307.x. Epub 2012 Feb 9.	Anim Genet. 2012	PMID:22497335

27	Genome-wide association analysis for feed efficiency in Angus cattle.	Rolf MM, Taylor JF, Schnabel RD, McKay SD, McClure MC, Northcutt SL, Kerley MS, Weaber RL.	Anim Genet. 2012 Aug;43(4):367-74. doi: 10.1111/j.1365-2052.2011.02273.x. Epub 2011 Oct 24.	Anim Genet. 2012	PMID:22497295
28	Evaluation of Bovine chemerin (RARRES2) Gene Variation on Beef Cattle Production Traits.	Lindholm-Perry AK, Kuehn LA, Rempel LA, Smith TP, Cushman RA, McDaneld TG, Wheeler TL, Shackelford SD, King DA, Freetly HC.	Front Genet. 2012 Mar 29;3:39. doi: 10.3389/fgene.2012.00039. eCollection 2012.	Front Genet. 2012	PMID:22479267
29	Whole-genome QTL scan for ultrasound and carcass merit traits in beef cattle using Bayesian shrinkage method.	Nalaila SM, Stothard P, Moore SS, Li C, Wang Z.	J Anim Breed Genet. 2012 Apr;129(2):107-19. doi: 10.1111/j.1439-0388.2011.00954.x. Epub 2011 Sep 8.	J Anim Breed Genet. 2012	PMID:22394233
30	Association, effects and validation of	Lindholm-Perry AK, Sexten AK, Kuehn LA, Smith TP, King DA, Shackelford	BMC Genet. 2011 Dec 14;12:103. doi: 10.1186/1471-2156-12-103.	BMC Genet. 2011	PMID:22168586

	polymorphisms within the NCAPG - LCORL locus located on BTA6 with feed intake, gain, meat and carcass traits in beef cattle.	SD, Wheeler TL, Ferrell CL, Jenkins TG, Snelling WM, Freetly HC.			
